# Comparative Analysis of Metabolome and Transcriptome in Different Tissue Sites of *Aquilaria sinensis* (Lour.) Gilg

**DOI:** 10.3390/molecules29051075

**Published:** 2024-02-29

**Authors:** Anjun Wang, Juan Liu, Luqi Huang

**Affiliations:** 1 School of Pharmacy, Jiangsu University, Zhenjiang 212013, China; wanganjun1216@163.com; 2 State Key Laboratory for Quality Ensurance and Sustainable Use of Dao-di Herbs, National Resource Center for Chinese Materia Medica, Chinese Academy of Chinese Medical Sciences, Beijing 100700, China

**Keywords:** *Aquilaria sinensis*, transcriptome, metabolome, sesquiterpene

## Abstract

The resinous stem of *Aquilaria sinensis* (Lour.) Gilg is the sole legally authorized source of agarwood in China. However, whether other tissue parts can be potential substitutes for agarwood requires further investigation. In this study, we conducted metabolic analysis and transcriptome sequencing of six distinct tissues (root, stem, leaf, seed, husk, and callus) of *A. sinensis* to investigate the variations in metabolite distribution characteristics and transcriptome data across different tissues. A total of 331 differential metabolites were identified by chromatography–mass spectrometry (GC-MS), of which 22.96% were terpenoids. The differentially expressed genes (DEGs) in RNA sequencing were enriched in sesquiterpene synthesis via the mevalonate pathway. The present study establishes a solid foundation for exploring potential alternatives to agarwood.

## 1. Introduction

*Aquilaria sinensis* (Lour.) Gilg, belonging to the genus *Aquilaria* (Thymelaeaceae), exhibits a wide distribution across various Asian countries such as China, Vietnam, and Laos, among others [1]. It holds a prominent position as the sole primary source of domestic medicinal agarwood in the Chinese Pharmacopoeia, possessing significant medicinal and economic value. The aromatic resin known as agarwood is secreted in the wounds of the stems, and it is activated by plant defense responses when subjected to injury, insect pests, or microbe-attacked [2,3].The use of agarwood in traditional Chinese medicine encompasses pain relief, digestion promotion, antiemetic effects, and sedation [4]. Contemporary pharmacological research has revealed that agarwood essential oil possesses antioxidant, neuroprotective, antibacterial, and sedative properties [5]. Moreover, agarwood finds extensive application in the perfumery industry as well as in incense and sculpting collections [6]. Due to its extensive market demand and protracted formation process, wild agarwood resources have been exploited to the point of extinction, leading to a severe depletion of natural agarwood sources. To ensure the protection and sustainable development of wild agarwood resources, all *Aquilaria* spp. have been listed in Appendix II of the Convention on International Trade in Endangered Species of Wild Fauna and Flora (CITES, http://checklist.cites.org, accessed on 1 January 2024). Whether other tissue parts and tissue culture materials could be used as potential substitutes for agarwood in the future has been the novel focus of research.

Currently, the stem of *A. sinensis* serves as the primary source of agarwood. It is worth exploring whether similar components of agarwood exist in other tissue parts and callus materials. The study revealed significant differences in the structure of agarwood isolates compared to various non-resinous tissues. Sesquiterpenes and 2-(2-phenylethyl) chromone derivatives are identified as the principal and distinctive chemical constituents of agarwood [7,8,9]. The sesquiterpenes in agarwood can be classified into various groups based on their skeletal structures, including agarofurans, agarospiranes, cadinanes, guaianes, eudesmanes, zizaanes, eremophilanes, prezizaanes, humulanes, and others [10]. In addition, agarwood also contains various secondary metabolites, including aromatic hydrocarbons, triterpenoids, steroids, and alkaloids [11]. To date, over 200 compounds have been isolated from various parts of *A. sinensis*, including leaves, twigs, barks, and roots. These compounds mainly consist of sesquiterpenoids, 2-(2-phenethyl)chromonone, diterpenoids, triterpenoids, steroids, and aromatic agents [12]. Flavonoids were widely distributed in stems and leaves [13,14,15]. Benzophenones were primarily found in leaves, flowers, and pericarp, with a lesser distribution in stems [16,17,18]. Xanthones exhibited a specific distribution mainly in leaves and fruits [19], while lignans were predominantly present in stems [20,21]. Additionally, phenolic compounds were mainly distributed in leaves, stems, and bark [22,23]. Nitrogen-containing compounds and nucleosides commonly occur in leaves and petioles [21,24]. A small amount of 2-2-phenethyl chromonone was detected in leaves, stems, and cell suspension cultures [23,24,25]. The distribution of diterpenoids was primarily observed in seeds, stems, and leaves [26,27,28], whereas triterpenoids and steroids were found in leaves, fruits, peels, petioles, and stems [7,11,13,21,22,23,24,26,29,30]. However, these reports solely focused on the variances in compound types and chemical structures, failing to comprehensively compare the distribution characteristics and content disparities of metabolites across different parts, particularly terpenoid metabolites.

Plant terpenoids are a class of natural products characterized by the presence of two or more isoprene units on their fundamental carbon scaffolds. They can be categorized into various subclasses, such as monoterpenes, sesquiterpenes, diterpenes, and triterpenes, based on the number of isoprene units they contain [31]. The synthesis of terpenoids can be classified into two major pathways: the mevalonate (MVA) pathway and the 2C-methyl-d-erythritol-4-phosphate (MEP) pathway. The former is capable of producing sesquiterpenes and triterpenes in the cytoplasm of almost all eukaryotes and archaea, while the latter is restricted to synthesizing monoterpenes, diterpenes, and triterpenes in plastids found in eubacteria, algae, higher plants, and protists [32,33,34,35,36,37]. In the MVA pathway, acetyl-CoA is catalyzed by a series of enzymes to form the precursor isopentenyl diphosphate (IPP), with 3-hydroxy-3-methylglutaryl-CoA reductase (HMGR) acting as the rate-limiting enzyme in this pathway [38]. In the MEP pathway, the glycolytic products glyceraldehyde-3-phosphate and pyruvate undergo several enzymatic reactions to produce dimethyl allyl diphosphate (DMAPP). The rate-limiting enzymes in this pathway are 1-deoxy-D-xylulose-5-phosphate synthase (DXS) and 1-deoxy-D-xylulose-5-phosphate reducing isomerase (DXR) [39]. Reversible conversion of isopentenyl diphosphate (IPP) and dimethylallyl diphosphate (DMAPP) can be achieved through the action of isopentenyl diphosphate isomerase (IDI) [31]. These two substrates are catalyzed by farnesyl diphosphate synthase (FPS) to produce farnesyl diphosphate (FPP) [40], and by geranylgeranyl pyrophosphate synthase (GPPS) to produce geranyl pyrophosphate (GPP) and geranylgeranyl pyrophosphate (GGPP) [41]. Among them, FPP serves as the direct precursor for the biosynthesis of sesquiterpenes and triterpenes [42], GPP acts as the direct precursor for monoterpene synthesis, and GGPP functions as the direct precursor for the biosynthesis of diterpenes, carotenoids, and gibberellins [43]. Ultimately, these precursors undergo transformation into various types of sesquiterpenes, mediated by terpene synthases [44].

The majority of sesquiterpene compounds found in agarwood undergo catalysis by sesquiterpene cyclases to generate FPP. Consequently, the genes responsible for encoding sesquiterpene cyclases, particularly guaiacene synthase (GSs), which is widely distributed in agarwood, and their associated reaction mechanisms have garnered significant attention. The treatment of *A. crassna* with methyl jasmonate (MJ) was found by Kumeta and Ito [45] to promote the expression of the *δ*-guaiac synthase gene, leading to the induction of *α*-guaiene, *α*-humulene, and *δ*-guaiene production. Additionally, a series of studies [4,34,45,46,47,48,49] have demonstrated that physical or chemical trauma, such as mechanical injury, exposure to marijuana (MJ), or treatment with formic acid (FA), can induce the expression of FPS-related genes. This suggests a clear association between tissue injury and sesquiterpenoid synthesis at the level of gene expression.

Plant metabolomics is a crucial analytical tool that has been extensively utilized to assess the dynamic alterations of metabolites across various tissues, species, and developmental stages [50]. RNA-seq-based molecular bioprospecting approaches enable the identification of regulatory genes and account for variations in the expression levels of biosynthetic genes in relation to metabolite content [51]. A combined analysis of transcriptome data and metabolic profiles facilitates the identification of functional genes and the elucidation of primary and secondary metabolic pathways in plants [52,53]. The integration of transcriptomics and metabolomics studies enables the exploration of the correlation between metabolites and genes, thereby elucidating the molecular regulatory mechanisms underlying metabolite dynamics [54,55]. In this study, the omics approach was employed to integrate volatile organic compounds (VOCs) analysis based on GC-MS and transcriptome analysis based on RNA-seq. The aim was to screen and analyze differential metabolites and differentially expressed genes using Gene Ontology (GO) and Kyoto Encyclopedia of Genes and Genomes (KEGG) database enrichment analysis. The present study investigated the distribution of content differences and transcriptional profiles of sesquiterpenes and other volatile components in various tissue parts of *A. sinensis*. Additionally, it explored the compositional similarities between different tissue parts of *A. sinensis* and agarwood, thereby establishing a foundation for further exploration into the compositional differences and related molecular regulatory networks between resinous and non-resinous parts of *A. sinensis*, as well as the search for potential substitutes.

## 2. Results

### 2.1. Phenotypic Analysis of Aquilaria sinensis

**Figure 1:**
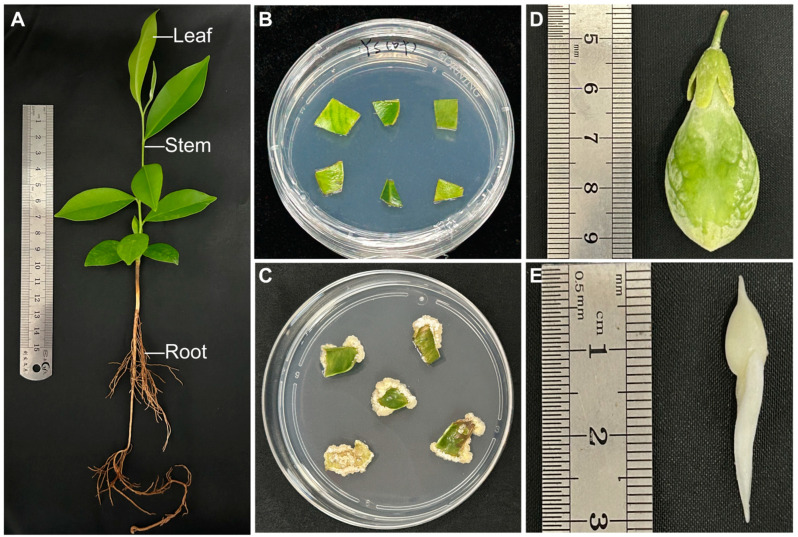
Morphological characteristics observation of *Aquilaria sinensis*. (**A**) Seedling; (**B**) leaf before callus induction; (**C**) leaf callus; (**D**) husk; (**E**) seed.

In order to analyze the metabolome and transcriptome of *Aquilaria sinensis* in different tissue parts, we initially induced the callus of leaves (Figure 1B). Following a 30-day induction period, the callus exhibited robust growth, appearing healthy, white, and fragile (Figure 1C). The seedlings of *Aquilaria sinensis* (Figure 1A) height measures between 13 and 15 cm, exhibiting a well-developed root system, robust health without any signs of pests or diseases, and displaying excellent growth. The stem surface is sparsely puberulent. The leaf is subleathery and elliptic, with an apical apex and a broadly cuneate base, which measured 3–9 cm in length. Furthermore, the upper surface of the leaves is bright, while both surfaces are glabrous. The husk is an oval sphere, measuring 3–4 cm in size, exhibiting a green coloration and featuring a dense covering of yellow hairs on its surface (Figure 1D). The seed is ovoid in shape, measuring approximately 1 cm in length. And the seed possesses a basal appendage that is about 1.5 cm long; in addition, the apex is adorned with short cusps (Figure 1E).

### 2.2. Metabolomi Analysis of Distinct Tissue Sites in Aquilaria sinensis

**Figure 2:**
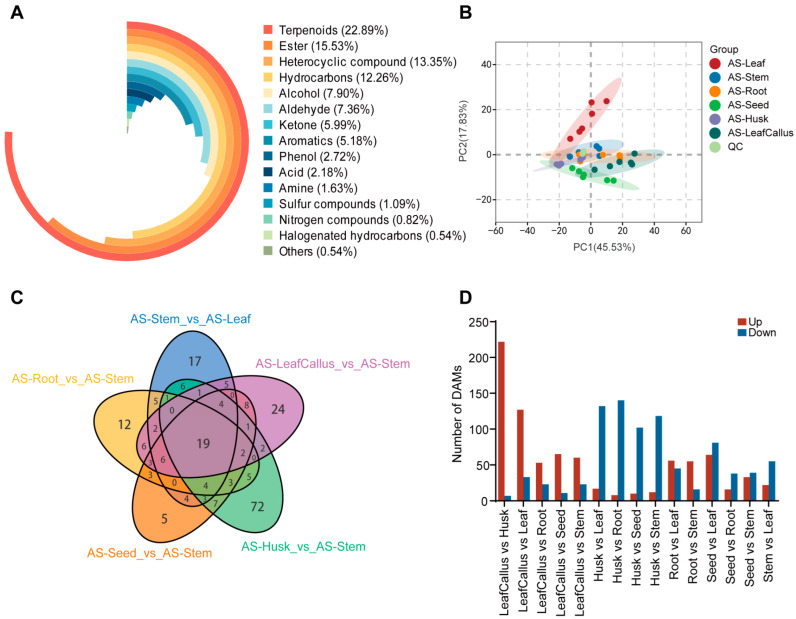
Analysis of volatile components in different tissue parts of *Aquilaria sinensis*. (**A**) The composition of volatile compounds in the class. (**B**) The principal component analysis (PCA) score plots of *A. sinensis* between six different tissue parts and quality control (QC) samples. (**C**) Venn diagram illustrating the distribution of various VOCs across different tissue components of *A. sinensis*. Each circle in the graph represents a comparison group. The numbers in the overlaps between the circles represent the number of common metabolites among the comparison groups. The number in the circle without any overlaps represents the number of distinct metabolites among the comparison groups. (**D**) Statistics of differential accumulation metabolites (DAMs) with different comparisons.

In order to further investigate the variations in metabolites across different tissue components, a total of 367 volatile compounds were analyzed using gas chromatography–mass spectrometry (GC-MS) in six distinct tissue parts. These metabolites encompassed various classes, including 8 acids, 29 alcohols, 27 aldehydes, 6 amines, 19 aromatics, 57 esters, 2 halogenated hydrocarbons, 49 heterocyclic compounds, 45 hydrocarbons, 22 ketones, 3 nitrogen compounds, 10 phenols, 4 sulfur compounds, 84 terpenoids, and 2 others. Among them, terpenoids species accounted for the majority, representing 22.89% of the total. Esters, heterocyclic compounds, and hydrocarbons were the next, accounting for 15.53%, 13.35%, and 12.26%, respectively. Alcohols, aldehydes, ketones, aromatics, and phenols comprised only 7.9%, 7.36%, 5.99%, 5.18%, and 2.72%, respectively. The remaining compound species made up a relatively small proportion (Figure 2A). The ketone cashmeran displayed its peak levels in roots, seeds, husks, and leaf callus. Additionally, the ketone 4-hexen-3-one exhibited the highest concentration in leaves, while the phenolic compound coniferyl alcohol showed the highest abundance in stems.

Quality control (QC) samples were incorporated into every 10 monitored analysis samples during the analysis process to ensure the reproducibility and reliability of data across different tissue sites. The overlap plot of total ion current (TIC) and various quality control samples demonstrated a high degree of concurrence in the total ion current curve for metabolite detection, with consistent retention time and peak intensity. This indicates excellent signal stability of the mass spectrometer when detecting the same sample at different time points (Figure S1).

The principal component analysis (PCA) of the samples, including the quality control samples, can preliminarily determine the overall difference in metabolites between sample groups and assess the extent of variation within each group. The PCA results demonstrate the discernible separation pattern among metabolic groups and provide insights into potential variations within each group, thereby serving as an effective approach for comparing different sample cohorts in the field of metabolic research [56]. The principal component analysis revealed that PC1 and PC2 accounted for 45.53% and 17.83% of the total variance contribution rate, respectively, effectively distinguishing between different tissue samples (Figure 2B). The sample group within each tissue site exhibited minimal variation, while a distinct pattern of differentiation was observed between different tissue sites. The patterns of metabolite accumulation exhibited the highest similarity between roots and stems, while showing the greatest dissimilarity in leaves compared to other tissue sites.

The identification of differential metabolites involved the application of orthogonal partial least squares discriminant analysis (OPLS-DA) to extract components from both the independent variable X and the dependent variable Y, followed by the computation of their correlation [57]. In this study, the OPLS-DA method was employed to identify the variables accountable for the disparities observed across six distinct tissue sites (Figure S2). The results demonstrated that the Q2 values of all control groups exceeded 0.9, indicating the stability of the model and the reliability of the data. In addition, OPLS-DA score plots showed that the six tissue sites were well separated in pairs (Figure S2). 

### 2.3. Identification and Classification of Differentially Accumulation Metabolites (DAMs) 

Based on VIP ≥ 1 and fold change ≥ 2 or ≤0.5, the analysis revealed a total of 331 differential metabolites exhibiting significant changes (Table S1). Most of the differential volatile metabolites were identified between the husks and leaf callus, with a total of 229 types. This was followed by combinations of leaves and leaf callus (160 types), leaves and husks (149 types), roots and husks (148 types), as well as leaves and seeds (145 types). Only 54 different volatile components were found between roots and seeds, while 76 different volatile components were identified between leaf callus and roots, as well as leaf callus and seeds. After intersecting the control groups in a Venn diagram, only 19 metabolites were found to be common among them. Overall, volatile components exhibited distinct spatial distribution patterns across various tissue sites (Figure 2C). The callus exhibited a greater number of differential metabolites in leaves and husks while showing fewer differences with roots, stems, and seeds (Figure 2D). The metabolic pattern of leaves exhibited significant changes after callus formation, resembling the metabolic patterns observed in roots, stems, and seeds.

**Figure 3:**
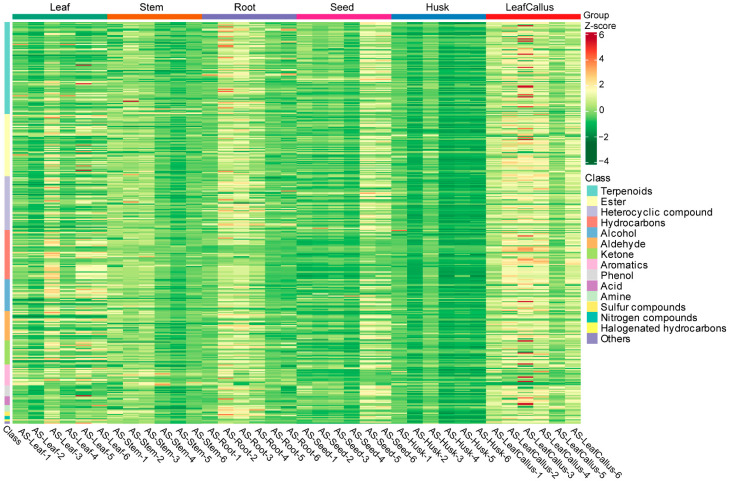
The heat map depicts the clustering of differential metabolites. Each sample was represented by a column, and each metabolite was represented by a row. Each column corresponds to the abundance of a specific metabolite, indicated by a distinct color. The up-regulated and down-regulated metabolites were indicated by different shades of red and green, respectively.

Additionally, the heatmap clearly depicted their classification into six distinct categories (Figure 3), thereby indicating variations in metabolite content across six different tissue sites. Among them, terpenoids, ketones, and other volatile metabolites generally accumulate at high levels in leaf callus. The accumulation of various metabolites in the husk was minimal. These findings suggest that there are variations in tissue metabolite accumulation patterns among different types of *A. sinensis*.

The top 20 differential metabolites were identified in distinct groups (Figure S3). In the comparison between husk and leaf, the heterocyclic compound 2,2′-Isopropylidenebis(5-methylfuran) was found to be significantly up-regulated, while the ester compound Methyl 3-methyl-2-butenoate was significantly down-regulated. The ketone compound 4-Hexen-3-one was consistently up-regulated as a highly significant differential metabolite, whereas the heterocyclic compound Isopropyl methoxy pyrazine and the alcohol compound trans-2-Nonen-1-ol were both significantly down-regulated. The ketone compound cyclopentanone exhibited a highly significant up-regulation in husk vs. stem, whereas the terpenoid compound cis-*α*-bergamotene showed a highly significant down-regulation. The significantly down-regulated differential metabolites in leaf callus vs. husk/leaf/stem, root vs. leaf/stem, and seed vs. leaf/stem were exclusively ketone compound 4-Hexen-3-one. Conversely, the highly significantly up-regulated differential metabolites, except for leaf callus/root vs. leaf, comprised heterocyclic compound 2,2′-Isopropylidenebis(5-methylfuran), ketone compound Cyclopentanone in root/seed vs. stem comparison, and the remaining compounds were ketone compound 3-Acetylphenol and alcohols 6,10-dimethylundeca-5,9-dien-2-ol along with heterocyclic compounds such as 5-Ethenyl-4-methylthiazole. In the comparison between leaf callus and root, 6,10-dimethylundeca-5,9-dien-2-ol exhibited the most significant up-regulation among metabolites, whereas Isopropyl methoxy pyrazine showed the most significant down-regulation. In the comparison between leaf callus and seed, an aromatic compound [(S)-1-((S)-1-Isopropyl-but-3-enyloxy)-ethyl]-benzene was highly up-regulated as a metabolite of great significance, while trans-2-Nonen-1-ol displayed a highly significant down-regulation. Among seed and root comparisons, 1-ethenyl-cyclohexanol emerged as the most significantly up-regulated metabolite, whereas (+)-β-Cedrene demonstrated the most significant down-regulation. Furthermore, in the stem vs. leaf comparison analysis, the results indicated that 2,2′-isopropylidenebis(5-methylfuran) experienced substantial up-regulation while octane, 5-ethyl-2-methyl-, underwent considerable down-regulation. In addition, the leaf callus exhibited a higher abundance of up-regulated metabolites and a lower abundance of down-regulated metabolites compared to other tissue sites.

**Figure 4:**
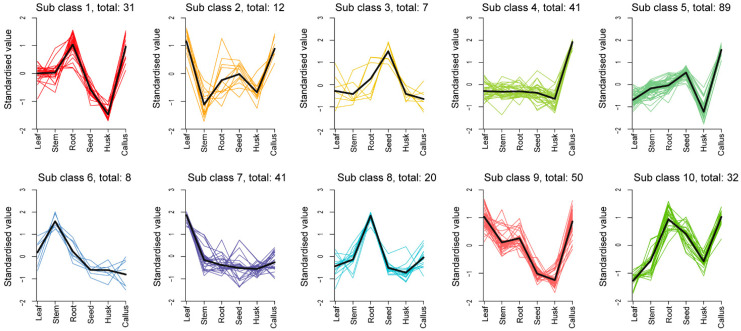
K-means cluster diagram of differential metabolites. The abscissa represents the sample group names. The ordinate represents the normalized relative contents of metabolites. The subclass represents the class number of metabolites exhibiting the same trend of change.

To identify the prevailing patterns in the distribution of differential metabolites across various tissue sites, we conducted K-means cluster analysis (Figure 4, Table S2). The findings revealed that these trends could be categorized into 10 distinct subclasses. Specifically, subclass 1 to subclass 10 encompassed clusters of 31, 12, 7, 41, 89, 8, 41, 20, and 50 metabolites, respectively. The most volatile components (89), primarily terpenoids, including 11 sesquiterpenes, were found in subclass 5. The data indicated that terpenoids were more commonly detected in leaf callus, followed by seeds, and less frequently observed in the husk. In addition, the predominant constituents in subclasses 1, 2, and 3 were esters, terpenoids, and esters, heterocyclic compounds, respectively. Hydrocarbons emerged as the major compounds in subclasses 4, 6, and 9. Esters dominated subclass 7, whereas terpenoids prevailed in subclasses 8 and 10. The accumulation of subclasses 1, 2, 4, 5, 9, and 10 was significantly observed in callus tissue. Subclass 3 exhibited significant accumulation in seeds. Subclass 6 showed significant accumulation in stems, while subclasses 1, 8, and 10 were found to accumulate significantly in roots. Leaves demonstrated a significant accumulation of subclasses 2, 7, and 9.

### 2.4. Enrichment Analysis of the KEGG Metabolic Pathway for DAMs

The KEGG database is a comprehensive resource that provides annotations on the biosynthetic pathways and associated gene functions of metabolites in plants. The differential metabolites from each tissue site were enriched and categorized into distinct metabolic pathways, with the primary pathways associated with the differential metabolites between groups being represented by bubble plots (Figure S4). Based on the enrichment outcomes of these differential metabolic pathways, the “Sesquiterpenoid and triterpenoid biosynthesis” pathway exhibited significant enrichment in leaf callus vs. leaf, root vs. leaf, root vs. stem, and stem vs. leaf controls (*p*-value < 0.05). Furthermore, the enrichment analysis of leaf callus vs. root comparison revealed a *p*-value < 0.05 for “Metabolic pathways”. Pathway analysis of differential metabolites in seed vs. leaf also demonstrated a *p*-value < 0.05 in the enrichment analysis; these three pathways were “Biosynthesis of secondary metabolites”, “Phenylpropanoid biosynthesis”, “Biosynthesis of various plant secondary metabolites”.

### 2.5. Analysis of Volatile Organic Compounds Related to Sesquiterpenes in A. sinensis

**Figure 5:**
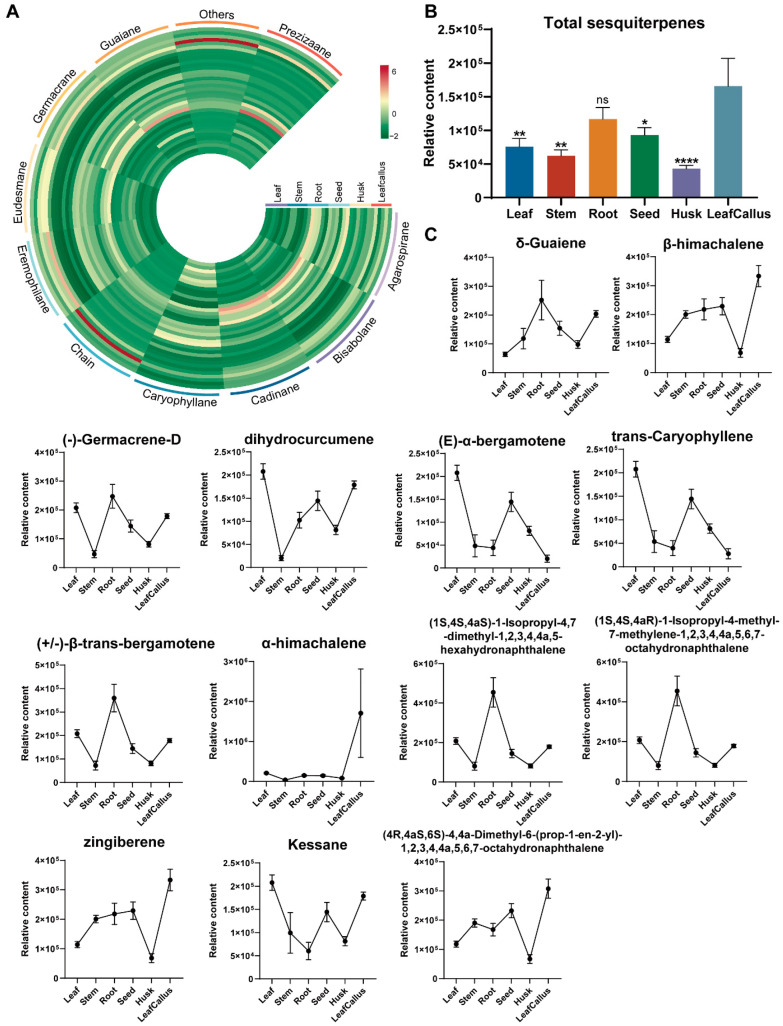
Analysis of sesquiterpenes in various tissue parts of *A. sinensis*. (**A**) Distribution of differentially accumulated sesquiterpenes. (**B**) The relative contents of total sesquiterpenes. ns, *p* > 0.05; *, *p* < 0.05; **, *p* < 0.01; ****, *p* < 0.0001, *n* = 6. (**C**) The relative contents of a subset of sesquiterpenes. Data are expressed as mean ± standard error of the mean (SEM); *n* = 6.

The primary constituents of agarwood are predominantly categorized as sesquiterpenes and 2-(2-phenethyl) chromones. The present study involved a comprehensive analysis of sesquiterpene composition in various tissue components of *A. sinensis*, revealing the detection of 43 distinct sesquiterpenes, which collectively accounted for approximately 51% of the overall terpenes. These sesquiterpenoids were composed of a variety of types, including one eremophilane, one prezizaane, two caryophyllanes, two eudesmanes, two germacranes, three agarospiranes, five guaianes, eight bisabolanes, nine cadinanes, and three chain forms, as well as seven other types of sesquiterpenes (Figure 5A). The accumulation of total sesquiterpenes was significantly higher in the callus, followed by the root, seed, leaf, and stem. The husk exhibited the lowest level of accumulation (Figure 5B). The germacranes exhibited significant accumulation in all leaves, stems, and seeds, while the agarospiranes showed substantial accumulation in both the husk and leaf callus.

The most abundant sesquiterpenes in stem were zingiberene and β-himachalene. The highest relative contents of sesquiterpenes in the husk and seed were *δ*-guaiene and (4R,4aS,6S)-4,4a-dimethyl-6-(prop-1-en-2-yl)-1,2,3,4,4a,5,6,7-octahy-dronaphthalene, respectively. The accumulation of *α*-himachalene, ©-*α*-bergamotene, kessane, dihydrocurcumene, (-)-germacrene-D, (+/−)-β-trans-bergamotene, trans-caryophyllene, (1S,4S,4aS)-1-isopropyl-4,7-dimethyl-1,2,3,4,4a,5-hex-ahyd-ronaphthalene, (1S,4S,4aR)-1-isopropyl-4-methyl-7-methylen-e-1,2,3,4,4a,5,6,7-octahydronap-hthalene was additionally found to be significantly higher in leaf, while the last two sesquiterpenes exhibited the highest levels in root, the first sesquiterpene was found to be most abundant in leaf callus (Figure 5C and Figure S5).

### 2.6. Transcriptome Analysis of Distinct Tissue Sites in Aquilaria sinensis 

To investigate the molecular mechanisms underlying variations in volatile metabolites across six tissue sites, including the root, stem, leaf, husk, seed, and leaf callus of *A. sinensis*, we conducted a transcriptome analysis of these specific tissue sites. After constructing a total of six RNA-seq libraries from tissue site samples (with each sample having three biological replicates), we obtained a dataset totaling 115.15 Gb after removing low-quality reads. Clean reads from each sample were mapped to the reference genome, with a success rate ranging from 82.6% to 91.37%. The Q30 value exceeded 92.98%, while the GC content surpassed 44.34%, indicating an exceptional quality of the RNA-seq results. The expression of a total of 28,850 genes was detected in the samples.

**Figure 6:**
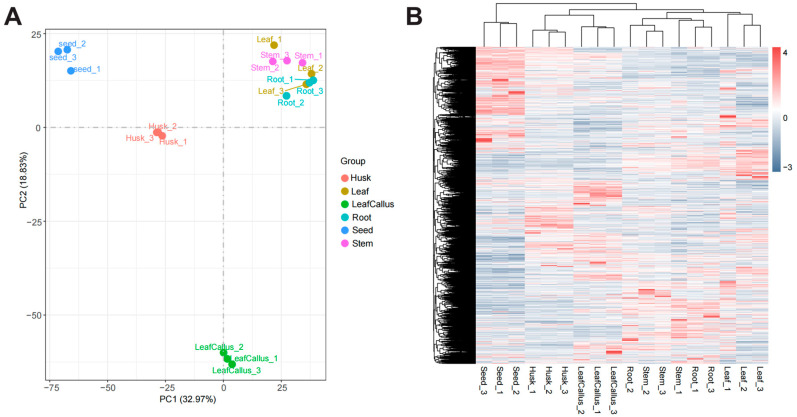
Analysis of differentially expressed genes. (**A**) PCA analysis of transcriptome data in different tissue parts of *Aquilaria sinensis*. (**B**) Heatmap based on hierarchical clustering analysis of differentially expressed genes (DEGs) in various tissue sites.

The principal component analysis diagram (Figure 6A) reveals a clear demarcation between different tissue components, with biological replicates within each sample exhibiting close clustering. This observation suggests strong inter-sample relationships and ensures the reliability of the data. The expression patterns of genes in the leaf, stem, and root exhibited similarities.

### 2.7. Identification and Classification of Differentially Expressed Genes (DEGs)

By selecting a remarkable differentially expressed genes (DEGs) threshold of choice, defined as |log2(fold change)| ≥ 1 and *p* ≤ 0.05, the fragments per kilobase of transcripts per million mapped fragments (FPKM) value was calculated for each individual gene. A total of 113,310 significant DEGs were observed in the 15 control groups. The highest number of DEGs was observed in seed and leaf callus, with a total of 11,005 genes identified. Among them, 5026 genes were up-regulated, while 5979 genes were down-regulated. On the other hand, stem and root callus exhibited the lowest number of differentially expressed genes, with only 808 identified. Out of these, 652 genes were up-regulated, while only 156 were down-regulated (Figure S6).

In order to further investigate the expression patterns of differentially expressed genes in various tissue sites and comprehend the variability in gene expression across diverse samples, we conducted hierarchical clustering analysis utilizing the Z-Score clustering method (Figure 6B). The clustering results demonstrated a high degree of similarity in gene expression patterns among biological replicates within each sample while revealing some variations in gene expression patterns across different tissue sites. The leaf callus and husk are classified into the same cluster, indicating a potential similarity in their gene expression patterns.

### 2.8. Functional Classification and Enrichment Analysis of DEGs 

**Figure 7:**
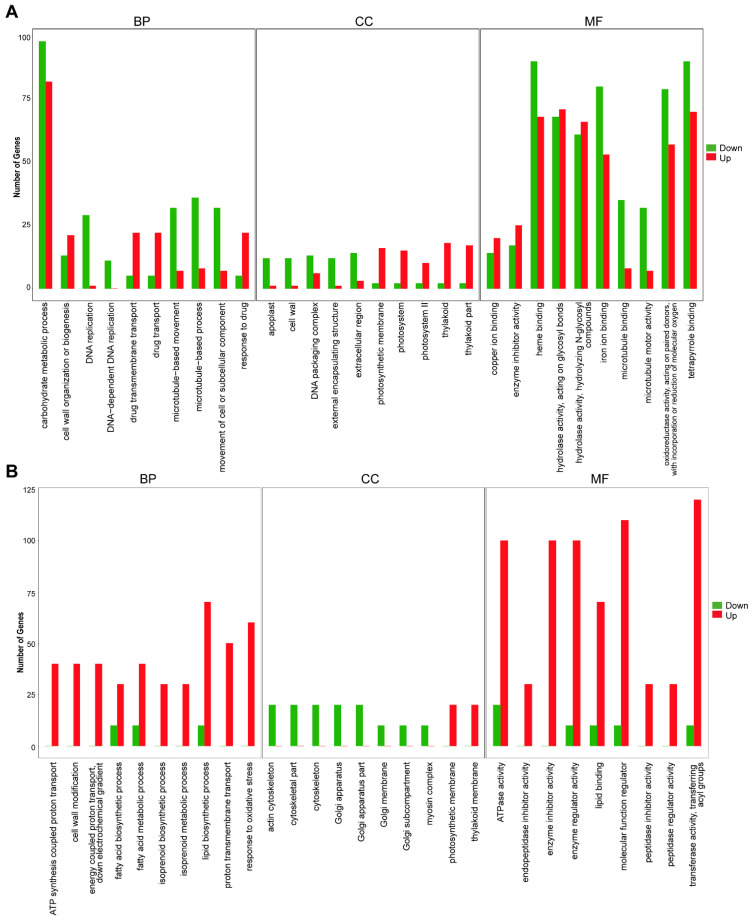
Analysis of differentially expressed genes. (**A**) Enriched GO terms of DEGs between husk and leaf callus. (**B**) Enriched GO terms of DEGs between stem and root.

To analyze the functions of differentially expressed genes and predict the functional classification of non-differentially expressed genes, we conducted GO enrichment analysis. We categorized gene sequences into three groups: biological process, cellular component, and molecular function. The husk and leaf callus libraries exhibited significant differences in “carbohydrate metabolic process”, “heme binding”, “hydrolase activity, acting on glycosyl bonds”, “iron ion binding”, “oxidoreductase activity, acting on paired donors with incorporation or reduction of molecular oxygen”, and “tetrapyrrole binding”. Most of these pathways primarily consist of down-regulated DEGs. The significant differences between the stem and root libraries are primarily concentrated in “lipid biosynthetic process”, “ATPase activity”, “enzyme inhibitor activity”, “enzyme regulator activity”, “molecular function regulato”, “transferase activity, transferring acyl groups”. Most of these pathways consist mainly of up-regulated DEGs (Figure 7 and Figure S7).

To conduct an analysis of the gene functional pathways associated with differentially expressed genes across various tissue sites, we performed a KEGG enrichment analysis. We selected the top 20 most significant pathways for visualization using scatter plots (Figure S8). The results of the KEGG enrichment analysis revealed a significant enrichment of differentially expressed genes in the metabolic pathways “ABC transporters”, “Starch and sucrose metabolism”, “Plant hormone signal transduction”, “Biosynthesis of various plant secondary metabolites”, “Flavonoid biosynthesis”, and “MAPK signaling pathway—plant”. The differentially expressed genes in the comparison group between the callus and the rest of the tissues were mainly concentrated in pathways involved in “ABC transporters”, “Biosynthesis of various plant secondary metabolites”, “Plant-pathogen interaction” and “Starch and sucrose metabolism” as well as other metabolic pathways. Notably, the “Diterpenoid biosynthesis” pathway was enriched exclusively in the control group of husk and leaf callus, while the “Pantothenate and CoA biosynthesis” pathway showed significant enrichment solely in the control group of root and leaf callus. Additionally, “Carbon metabolism” exhibited significant enrichment only in the control group of leaf and root.

### 2.9. Analysis of Sesquiterpene Biosynthetic Genes in A. sinensis

**Figure 8:**
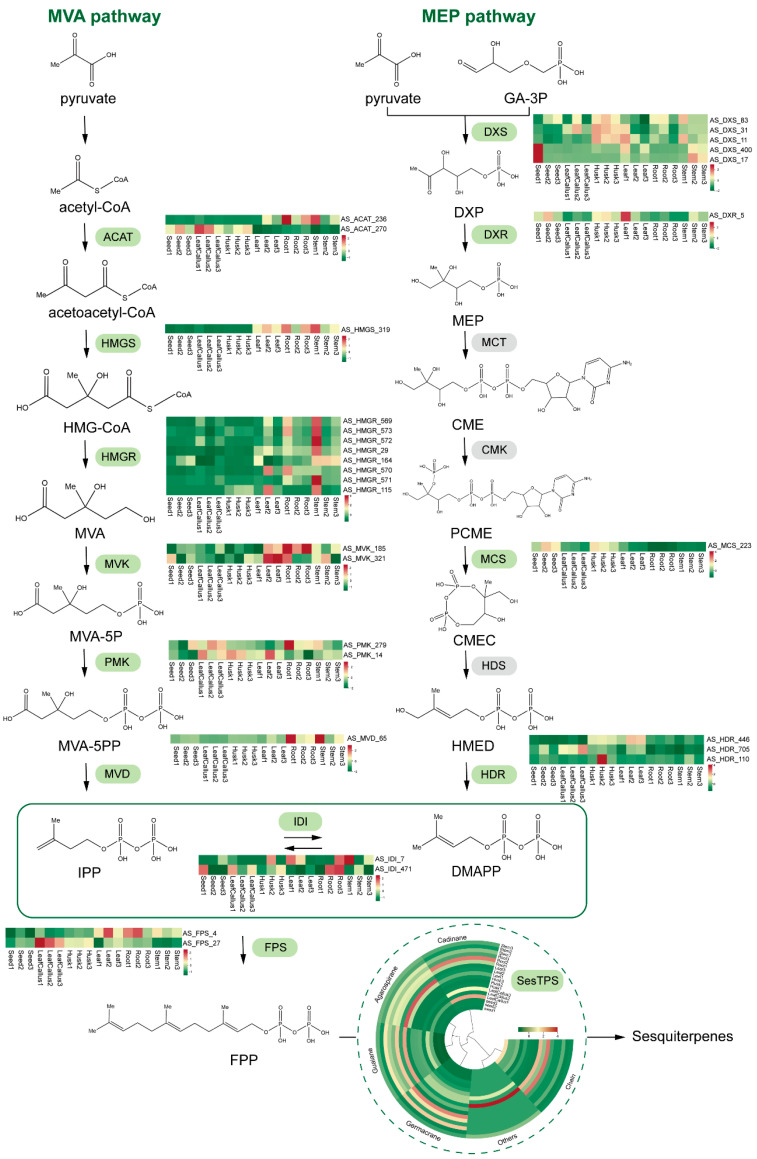
Schematic representation of DEG pathways involved in the sesquiterpene synthesis pathway. The color scale from green to red represents the expression level of DEGs from low to high. AACT: acetyl-CoA C-acetyltransferase; HMGS: 3-hydroxy-3-methylglutaryl CoA synthase; HMGR: 3-hydroxy-3-methylglutaryl coenzyme A reductase; MVK: mevalonate kinase; PMK: phosphomevalonate kinase; MVD: mevalonate diphosphate decarboxylase; DXS: 1-deoxy-D-xylulose 5-phosphate synthase; DXR: 1-deoxy-D-xylulose 5-phosphate reductoisomerase; MCT: mesaconate CoA-transferase; CMK: cytidylate kinase; MCS: 2-C-methyl-D-erythritol-2,4-cyclodiphosphate synthase; HDS: 4-hydroxy-3-methyl but-2-(E)-enyl diphosphate synthase; HDR: 4-hydroxy-3-methyl but-2-(E)-enyl diphosphate reductase; IDI: isopentenyl diphosphate isomerase; FPS: farnesyl-pyrophosphate synthetase; FPP: farnesyl pyrophosphate synthase; SesTPS: sesquiterpene synthase.

The gene expression profiles of the MVA and MEP pathways involved in terpenoid backbone biosynthesis were analyzed to characterize the sesquiterpene biosynthetic pathway across different tissue sites (Figure 8). A total of 28 unigenes associated with the biosynthetic pathway of the sesquiterpene precursor FPP were identified. Among them, 16 unigenes were identified as being associated with 6 enzymes in the MVA pathway, while 10 unigenes were identified as being associated with 4 enzymes in the MEP pathway responsible for synthesizing the key terpene precursors IPP and DMAPP. In addition, the annotation identified two unigenes as IDI, responsible for the reversible conversion of isomers IPP and DMAPP. Two unigenes were annotated as FPS, involved in the synthesis of FPP, a crucial precursor for sesquiterpenes. Furthermore, 46 unigenes were annotated as TPS, contributing to the synthesis of terpenoids with diverse skeletal structures. A total of 28 TPS were annotated related to sesquiterpene synthesis, comprising 10 gemmarane synthase genes, 8 guaiane synthase genes, 4 chain synthase genes, 3 cadinane synthase genes, 1 agarospirane synthase gene, and 2 others. The gene with the highest expression level was AS_ACAT_236 (ACAT) in leaf callus, seeds, and roots; AS_HMGS_319 (HMGS) in stems and leaves; and AS_HDR_446 (HDR) in husks. The expression patterns of TPS in roots and stems exhibited significant similarity. Within the callus, 60% of TPS contained sesquiterpene synthase genes, while 24% harbored monoterpene synthase genes and 16% possessed diterpene synthase genes.

### 2.10. Correlation Analysis of Transcriptome and Metabolome Expression Data

The enrichment analysis of differentially expressed genes (DEGs) and differential accumulation metabolites (DAMs) between each control group revealed significant enrichments in the “Biosynthesis of various plant secondary metabolites”, “Phenylpropanoid biosynthesis”, ”Glycolysis/Gluconeogenesis”, as well as amino acid metabolic pathways including “Arginine and proline metabolism”, “Tyrosine metabolism”, and “Cysteine and methionine metabolism”.

**Figure 9:**
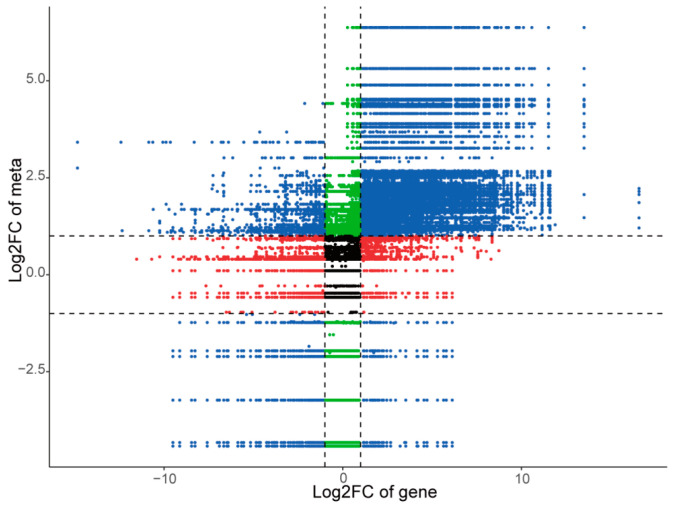
Nine quadrant diagram showing the correlation of differentially expressed genes and differentially accumulated metabolites between husk and leaf callus libraries.

To further investigate the regulatory network of differentially expressed genes, we conducted a correlation analysis between differential metabolites and differentially expressed genes in each comparison group. We then identified the correlations that met the criteria of an absolute value of the Pearson correlation coefficient greater than 0.8 and a *p*-value < 0.05. The fold differences of genes and metabolites corresponding to these correlations were visualized using a nine-quadrant plot (Figure 9 and Figure S9). Taking husk and leaf callus control groups as examples, our results demonstrated that genes and metabolites in quadrants 3 and 7 exhibited a positive correlation, indicating that gene expression changes positively regulated the abundance of metabolites. Specifically, there were 2125 differential genes positively regulating 267 differential metabolites. In quadrants 1 and 9, we observed a negative correlation between genes and metabolites, suggesting that gene expression changes negatively regulated the abundance of metabolites. In this case, there were 52 differential genes negatively regulating 192 differential metabolites. Among these findings, terpenoids were found to be regulated by a total of 1274 genes (320 negatively correlated, 955 positively correlated). Additionally, delta-guaiacene was found to regulate 36 genes (11 negatively correlated, 25 positively correlated). Genes located in quadrants 2, 4, 6, and 8 were not simultaneously up-regulated or down-regulated with their corresponding metabolite counterparts. In quadrant 5, no significant difference was observed in both gene expression and metabolite abundance. The findings suggest the existence of an intricate regulatory mechanism linking variations in metabolite accumulation and gene expression abundance across different tissue sites of *A. sinensis*. 

## 3. Discussion

The present study employed GC-MS-based metabolomics to detect and analyze the variations in volatile compound content among different plant parts (root, stem, leaf, seed, husk, and callus) of *A. sinensis*. A total of 367 metabolites were detected, with 84 terpenoids identified as the predominant compounds. In addition, a comprehensive analysis yielded a total of 331 differentially accumulated metabolites (DAMs) from 15 control groups. The primary volatile metabolites in stem, seed, husk, and leaf callus were terpenoids and ketones. In leaves, ketones and esters dominate as the main volatile metabolites. Meanwhile, roots exhibited terpenoids and heterocycles as their principal volatile metabolites. In this study, the majority of the differential metabolites, particularly terpenoids, exhibited higher expression levels in the callus, whereas chromones displayed lower expression levels in all non-resin parts compared to other sections. The application of salicylic acid (SA) and methyl jasmonate (MJ) has been employed to stimulate the production of aromatic compounds in callus and suspension cells of agarwood species. The results demonstrated that both SA and MJ significantly stimulated the biosynthesis of sesquiterpenoids, while they did not directly induce the production of chromones. Further investigation unveiled that sesquiterpenoids are synthesized in living cells, whereas chromones are likely derived from dying cellular debris [58]. Sesquiterpenes, which belong to the class of terpenoids, are considered crucial defensive secondary metabolites synthesized by agarwood in response to both biotic and abiotic stresses. Under mechanical stress, plants synthesize key molecules such as hydrogen peroxide (H_2_O_2_), jasmonic acid (JA), and salicylic acid (SA) [59], which trigger the biosynthesis of sesquiterpenes in agarwood, subsequently regulating defense responses against various abiotic and biotic stimuli [60,61,62,63]. A total of 43 sesquiterpenoids were isolated from six tissue sites of *A. sinensis*, which were categorized into 11 distinct types based on their backbone structures, predominantly comprising bisabolanes (8) and cadinanes (9) types. The accumulation of gemmaranes was found to be significant in leaves, stems, and seeds. On the other hand, agarospirane showed significant accumulation, specifically in the husk and leaf callus.

Additionally, RNA-seq was employed to conduct transcriptome sequencing across various tissues for further analysis of the metabolic regulatory network associated with variations in volatile component content among different tissues, as well as for identification of potential key genes involved in this process. The differential expression analysis showed that the number of differentially expressed genes identified in each control group surpassed 808. The annotation identified a total of 58 unigenes involved in the biosynthesis of sesquiterpenes. The main biosynthetic pathway of sesquiterpenes is the MVA pathway. ACAT is predominantly expressed in leaf callus, seed, and root tissues, while HMGS shows predominant expression in stem and leaf tissues. The gene HDR, involved in the MEP pathway for monoterpene, diterpene, and triterpene biosynthesis, exhibits high expression levels in the husk tissue. The observed outcome demonstrated a positive correlation with the accumulation of sesquiterpenes in the metabolome.

The efficacy of sesquiterpenes in conferring resistance against biotic and abiotic stresses has been extensively investigated, leading to numerous studies aimed at enhancing sesquiterpene production. In addition to inducing physical or chemical trauma, studies have also been conducted to enhance sesquiterpene formation by modulating the activity of rate-limiting enzymes in the biosynthetic pathway. The sesquiterpene synthetase 1 (ASS1) is a prototypical wound-induced enzyme that facilitates the synthesis of various sesquiterpene compounds, including *δ*-guaiene, *α*-guaiene, and *α*-humulene, in vitro [62]. Its promoter harbors a core sequence that responds to signals associated with tissue injury, while its key transcription factor, AsWRKY44, exerts inhibitory effects on ASS1 promoter activity [63]. Recent studies have revealed that RING3, a C3HC4-type E3 ubiquitin ligase, plays a crucial role in the degradation of AsWRKY44. This process effectively alleviates the inhibitory effect of AsWRKY44 on ASS1 and activates the expression of ASS1. Consequently, it positively regulates sesquiterpene biosynthesis in response to wound stimulation [62].

The metabolic pattern of the callus exhibited similarities to those of roots, stems, and seeds, whereas its transcriptional pattern showed the highest resemblance to husks, followed by roots and stems. This illustration implies that significant alterations in leaf metabolic and transcriptional profiles transpire subsequent to callus formation. In addition, both metabolic and transcriptional patterns displayed similarities between roots and stems. The pathway analysis revealed that the differentially expressed genes (DEGs) and differential accumulation metabolites (DAMs) were predominantly co-enriched in key metabolic pathways, including “Biosynthesis of various plant secondary metabolites”, “Phenylpropanoid biosynthesis”, and “Glycolysis/Gluconeogenesis”. Among them, the differential metabolites between leaf callus and other tissues were also enriched in the “Biosynthesis of cofactors”, and “Sesquiterpenoid and triterpenoid biosynthesis”. Additionally, the differentially expressed genes showed enrichment in “ABC transporters” and “MAPK signaling pathway-plant”. The ATP Binding Cassette-Type Transporters play an essential role in the transportation process of endogenous secondary metabolites [64], terpenoids [65], and plant defense against fungal pathogens [66]. Reversible protein phosphorylation plays a crucial regulatory role in wound signaling pathways. The MAPK signaling cascade has been demonstrated to participate in abiotic stress-induced signaling pathways across various species, including Arabidopsis [67]. The results of this study demonstrate the activation of defense signaling pathways during wound signaling and callus formation, facilitating an effective response to external stress through differential accumulation of sesquiterpenes and other secondary metabolites, as well as modulation of gene expression related to metabolism. It is plausible that there might be a mechanistic similarity with agarwood formation.

In addition, it has been reported [4] that there is a potential correlation between the formation of agarwood and the metabolism of starch and sugar. The utilization of sugar is essential for the promotion of plant growth and development. The presence of soluble sugars serves as an osmotic regulator, enabling the modulation of internal and external permeability in plant cells. This facilitates the maintenance of a relative equilibrium with the surrounding environment and enhances plant resilience to stress [68]. Healthy wood exhibited a substantial presence of starch particles, whereas damaged wood displayed degradation of starch particles accompanied by an increase in agarwood resin content. The differentially expressed genes involved in the “Starch and sucrose metabolism” pathway exhibited significant up-regulation in roots, leaves, and stems compared to calluses, suggesting a decrease in starch content following callus formation. This finding implies that calluses and alaceae may share similar regulatory patterns within this metabolic pathway.

The metabolite and transcriptome data in this study provide a comprehensive analysis of metabolic and transcriptional expression patterns across different tissues of *A. sinensis*, thereby establishing the fundamental basis for elucidating the correlation between volatile metabolites and genes in distinct tissue compartments of *A. sinensis*, as well as facilitating the exploration of potential substitutes for agarwood.

## 4. Materials and Methods

### 4.1. Plant Materials

The plant materials of *A. sinensis* originated from the mother tree of *A. sinensis* in Guangzhou Intangible Cultural Heritage Protection Park and were collected during the fruit period in June 2023. The fresh, young leaves were subjected to surface sterilization by immersing them in 75% ethanol for 30 s, followed by treatment with a 2% sodium hypochlorite solution for 6 min. Afterward, the leaves were rinsed five times with sterile water and placed onto Murashige-Skoog (MS) medium supplemented with 1.0 mg/L 6-Benzylaminopurine (6-BA) and 0.5 mg/L 1-naphthlcetic acid (NAA). The fresh leaf callus material was then harvested after a two-month incubation period at a temperature of 25 °C in darkness.

The collected materials were categorized into six groups based on different tissue sites: root, stem, leaf, seed, fruit shell, and leaf callus. After being frozen in liquid nitrogen immediately, all samples were stored at −80 °C until use. For GC-MS metabolomics analysis, six biological replicates were established for each group, and the experimental design involved the utilization of three plants per replicate group, with an equal proportion of different tissues from each plant being combined within each replicate group. For transcriptome analysis, three biological replicates were implemented for each group, meticulously blended to ensure homogeneous distribution. The microscopic observations of those microsections were conducted by an Olympus BX51 microscope (Olympus corporation, Tokyo, Japan).

### 4.2. Volatile Metabolomic Analysis of Sample Preparation and Extraction

Volatile metabolome analysis was performed by a dynamic headspace sampling method. All samples were freeze-dried in a vacuum for 48 h. Subsequently, the freeze-dried samples were pulverized in liquid nitrogen and vigorously mixed, and approximately 0.2 g of each sample was weighed into a headspace bottle. Then, 0.2 g of NaCl powder and 20 µL (10 µg/mL) 3-Hexanone-2,2,4,4-d4 (CAS No. 24588-54-3) were added. Sample extraction was performed using automatic headspace solid-phase microextraction (HS-SPME). After agitation at 60 °C for 5 min, a DVB/CWR/PDMS extraction head with a diameter of 120 µm was inserted into the headspace bottle and allowed to extract for 15 min at elevated temperature before being resolved at 250 °C for an additional 5 min. Finally, the extracted compounds were separated and identified by GC-MS analysis. Prior to sampling, the extraction head was conditioned at a temperature of 250 °C for 5 min in a Fiber Conditioning Station [69,70].

### 4.3. Detection of Volatile Organic Compounds (VOCs)

The sample extracts were analyzed using a gas chromatography–mass spectrometry system (GC-MS/MS, 8890-7000D, Agilent, Santa Clara, CA, USA) equipped with a 30 m × 0.25 mm ×0.25 μm DB-5MS (5% phenyl-polymethylsiloxane) GC capillary column (Agilent). High purity helium was used as the carrier gas with a constant flow rate of 1.2 mL/min. The injector temperature was maintained at 250 °C without a shunt, and the solvent delay time was set to 3.5 min. The programmed temperature started at 40 °C for an initial hold time of 3.5 min, then increased to 100 °C at a rate of 10 °C/min, followed by an increase to 180 °C at a rate of 7 °C/min, and finally raised to 280 °C at a rate of 25 °C/min for a duration of 5 min.

Mass spectra were recorded in electron impact (EI) ionization mode at 70 eV. The quadrupole mass detector, ion source, and transfer line temperatures were set, respectively, at 150, 230, and 280 °C. The MS was selected, and the ion monitoring (SIM) mode was used for the identification and quantification of analytes. To ensure the reproducibility of the analytical process, a quality control sample (prepared by blending samples) was incorporated into every 10 test samples during instrumental analysis. Volatile compounds were identified by comparing their mass spectra with the MetWare database (http://www.metware.cn/, accessed on 15 November 2023) and linear retention index [71]. The mass spectrum file of the sample was accessed using MassHunter quantitative software B.08.00 for integration and correction. The relative content of metabolites was analyzed using an internal standard semi-quantitative method [72]. 3-Hexanone-2,2,4,4-d4 (CAS No. 24588-54-3) was employed as the isotope internal standard. The calculation of relative content was performed according to the following formula:

$$X_{i}=\frac{V_{s}\times C_{s}}{V}\times \frac{I_{i}}{I_{s}}\times 10^{-3}$$

where *X_i_* is the content of compound *i* in the sample to be tested (µg/mL); vs. is the volume of internal standard (µL); *C_s_* is the concentration of internal standard (µg/mL); *V* is the volume of the sample to be tested (mL); *I_i_* and *I_s_* are the peak areas of the compound and the internal standard in the sample to be tested, respectively. The k-nearest neighbors (KNN) algorithm was employed to impute missing values in quantitative data, followed by the calculation of coefficient of variation (CV) values for quality control (QC) samples. Substances exhibiting CV values below 0.5 were retained. Unsupervised PCA (principal component analysis) was performed by the statistics function prcomp within R (www.r-project.org, accessed on 1 December 2023). For two-group analysis, differential metabolites were determined by VIP (VIP > 1) and absolute Log2FC (|Log2FC| ≥ 1.0). VIP values were extracted from the OPLS-DA result, which also contains score plots, and generated using the R package MetaboAnalystR 4.0 [73].

### 4.4. RNA Extraction and Transcriptome Sequencing

The RNA was extracted from the *A. sinensis* samples using the TRIzol Kit (Thermo Fisher Scientific, Waltham, MA, USA) in accordance with the manufacturer’s instructions. Subsequently, the quality of the extracted RNA was assessed utilizing a NanoDrop 2000 spectrophotometer (Thermo Fisher Scientific, Waltham, MA, USA). First-strand cDNA was synthesized using a random hexamer primer and M-MuLV Reverse Transcriptase(RNase H-). Second-strand cDNA synthesis was subsequently performed using DNA Polymerase I and RNase H. Remaining overhangs were converted into blunt ends via exonuclease/polymerase activities. After adenylation of the 3’ ends of DNA fragments, adaptors with hairpin loop structure were ligated to prepare for hybridization. In order to select cDNA fragments of preferentially 370~420 bp in length, the library fragments were purified with the AMPure XP system. Then PCR was performed with Phusion High-Fidelity DNA polymerase, Universal PCR primers, and index (X) primers. At last, PCR products were purified (AMPure XP system), and library quality was assessed on the Agilent Bioanalyzer 2100 system. Finally, the sequencing was conducted on the Illumina NovaSeq 6000. Gene expression was quantified by FPKM [74] using featurests (1.5.0-p3). Differential analysis of the genes was carried out using edgeR [75] with a negative binomial generalized log-linear model. For GO and KEGG annotations, custom datasets were constructed and used for enrichment analysis by clusterProfiler [76].

### 4.5. Transcriptome and Metabolome Conjoint Analysis

The Pearson correlation test was employed to investigate the association between differentially expressed genes (DEGs) and differentially abundant metabolites (DAMs) based on transcriptome and metabolome data. Only correlations with a Pearson correlation coefficient (PCC) value ≥ 0.7 and *p* ≤ 0.05 were considered significant. In R, DEGs and DAMs with a PCC ≥ 0.8 in each group were visualized using a nine-quadrant plot created with “ggplot2” and “getopt”. Additionally, DEGs and DAMs were mapped to the KEGG pathway database to obtain information about their shared pathways.

## 5. Conclusions

Agarwood, a resinous and fragrant wood, is highly valued for its use in medicine, perfumes, and incense across the world. The resinous stem of *Aquilaria sinensis* (Lour.) Gilg is the sole legally authorized source of agarwood in China. However, whether other tissue parts can be potential substitutes for agarwood requires further investigation. To address this, we conducted metabolic analysis and transcriptome sequencing of six distinct tissues (root, stem, leaf, seed, husk, and callus) of *A. sinensis* to investigate the variations in metabolite distribution characteristics and transcriptome data across different tissues. The volatile compound content was analyzed using chromatography–mass spectrometry (GC-MS), resulting in the identification of 331 differential metabolites through metabolomics analysis. Terpenoids constituted the majority of total metabolites (22.89%), with sesquiterpenes accounting for 51% of the identified terpenoids, predominantly distributed within the callus. The husk and leaf callus exhibited the highest degree of differential volatility, with a total of 229 distinct metabolite species identified. The RNA sequencing analysis revealed a significant enrichment of differentially expressed genes (DEGs) in the sesquiterpene synthesis pathway within the mevalonate pathway. The proportion of sesquiterpene synthase genes (60%) was significantly higher compared to other terpenoid synthase genes in callus. The transcriptional metabolic pattern demonstrated similarity between roots and stems. The present study provides comprehensive insights into the metabolome and transcriptome of *A. sinensis*, thereby establishing a solid foundation for future investigations on the biosynthesis and regulatory mechanisms underlying sesquiterpene production in *A. sinensis,* as well as potential alternatives to agarwood.

## Data Availability

Data are contained within the article and Supplementary Materials.

## Supplementary Materials

The following supporting information can be downloaded at https://www.mdpi.com/article/10.3390/molecules29051075/s1. Figure S1: The total ion current (TIC) of QC samples involves superimposing the ion flow diagram depicted in the figure; Figure S2: Orthogonal partial least squares-discriminant analysis (OPLS–DA) scores and OPLS-DA S-plot models between six different tissue sites; Figure S3: Bar Chart of DAMs between each comparison group (Top 20); Figure S4: KEGG enrichment of DAMs between each comparison group; Figure S5: Relative content of sesquiterpenes in six different tissue sites except for sesquiterpenes shown in Figure 5C; Figure S6: Volcano plot of differentially expressed genes among six different tissue sites; Figure S7: Enriched GO terms of DEGs between different control groups; Figure S8: Enriched Kyoto Encyclopedia of Genes and Genomes (KEGG) pathway of DEGs between different control groups; Figure S9: Nine quadrant diagram between different control groups; Table S1: Summary of 331 differential metabolites identified by GC-MS; Table S2: Ten subclasses of different volatile compounds in K-means cluster diagram.
