# Supplementary material for: Comparative Analysis of Metabolome and Transcriptome in Different Tissue Sites of Aquilaria sinensis (Lour.) Gilg
Source: Molecules. 2024 Feb 29;29(5):1075. doi: 10.3390/molecules29051075 (PMC10935121; doi:10.3390/molecules29051075)
Supplement: Supplementary file 1 [file molecules-29-01075-s001.zip › Supplementary Figure S1.pdf]

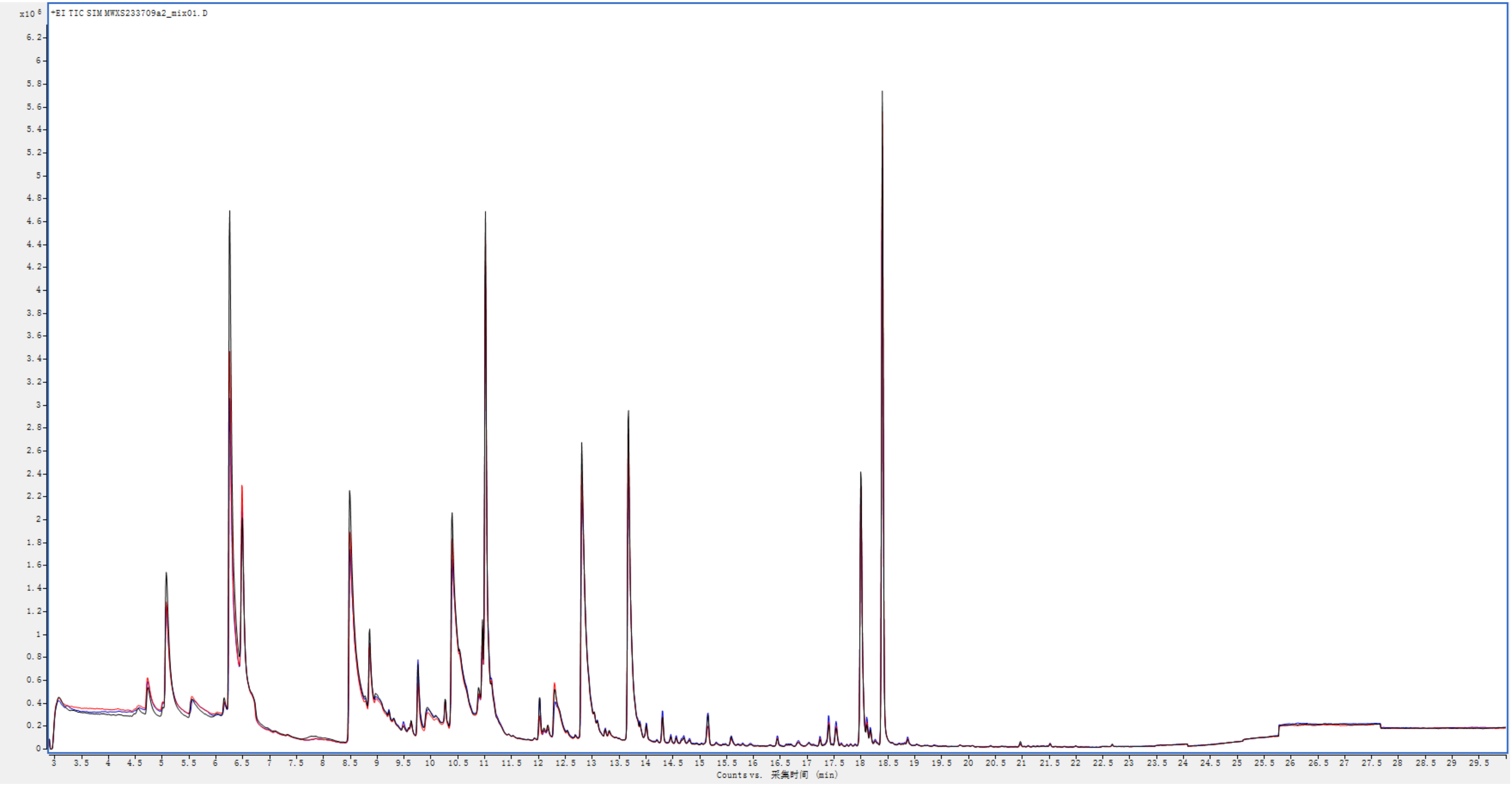


Supplementary Figure S1. The total ion current (TIC) of QC samples involves superimposing the ion flow diagram depicted in the figure.
