# Supplementary material for: Comparative Analysis of Metabolome and Transcriptome in Different Tissue Sites of Aquilaria sinensis (Lour.) Gilg
Source: Molecules. 2024 Feb 29;29(5):1075. doi: 10.3390/molecules29051075 (PMC10935121; doi:10.3390/molecules29051075)
Supplement: Supplementary file 1 [file molecules-29-01075-s001.zip › Supplementary Figure S2.pdf]

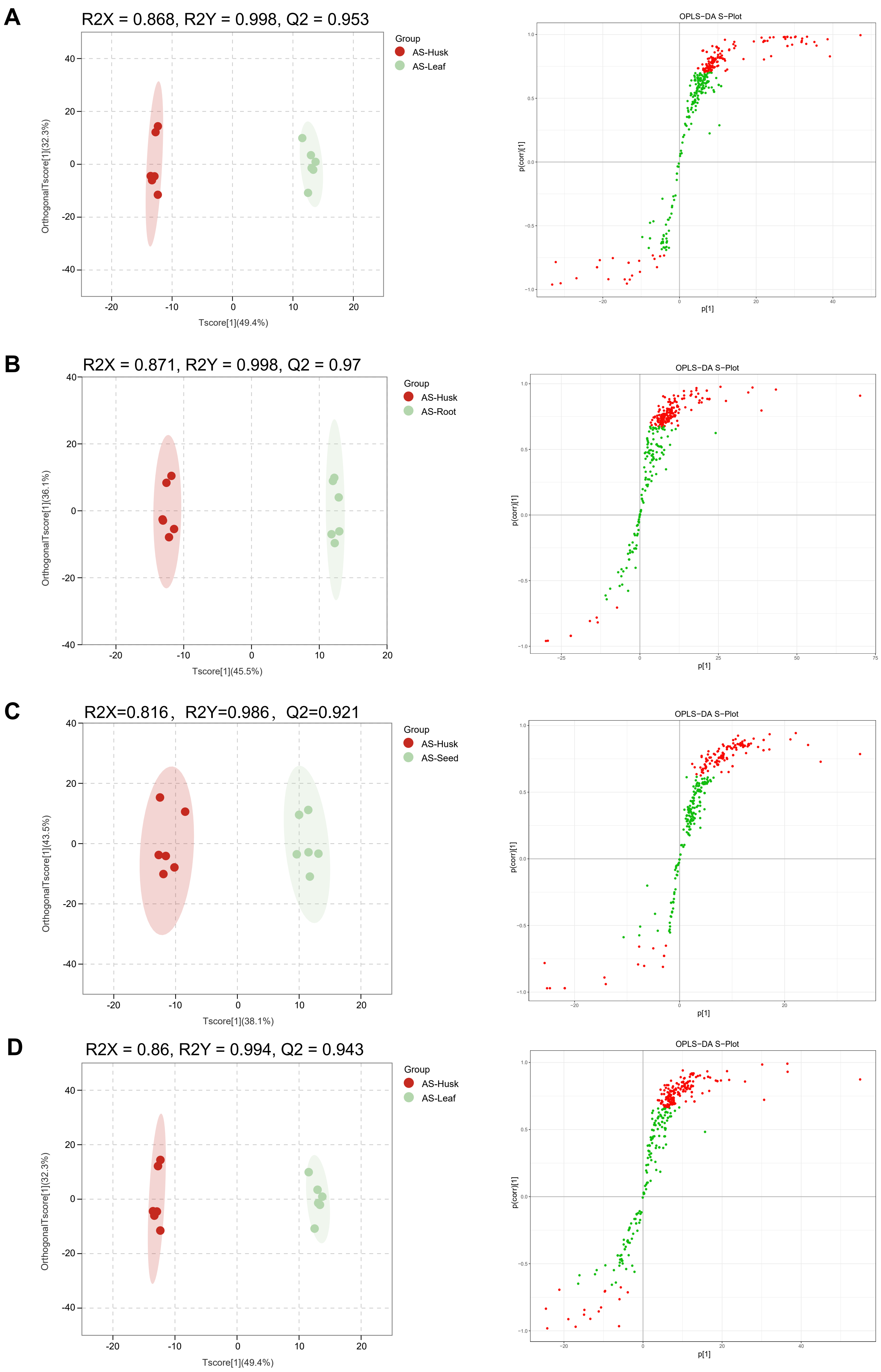

A
R2X = 0.868, R2Y = 0.998, Q2 = 0.953
Group
AS-Husk
AS-Leaf
OrthogonalTscore[1](32.3%)
Tscore[1](49.4%)
OPLS−DA S−Plot
p(corr)[1]
p[1]
B
R2X = 0.871, R2Y = 0.998, Q2 = 0.97
Group
AS-Husk
AS-Root
OrthogonalTscore[1](36.1%)
Tscore[1](45.5%)
OPLS−DA S−Plot
p(corr)[1]
p[1]
C
R2X=0.816，R2Y=0.986，Q2=0.921
Group
AS-Husk
AS-Seed
OrthogonalTscore[1](43.5%)
Tscore[1](38.1%)
OPLS−DA S−Plot
p(corr)[1]
p[1]
D
R2X = 0.86, R2Y = 0.994, Q2 = 0.943
Group
AS-Husk
AS-Leaf
OrthogonalTscore[1](32.3%)
Tscore[1](49.4%)
OPLS−DA S−Plot
p(corr)[1]
p[1]

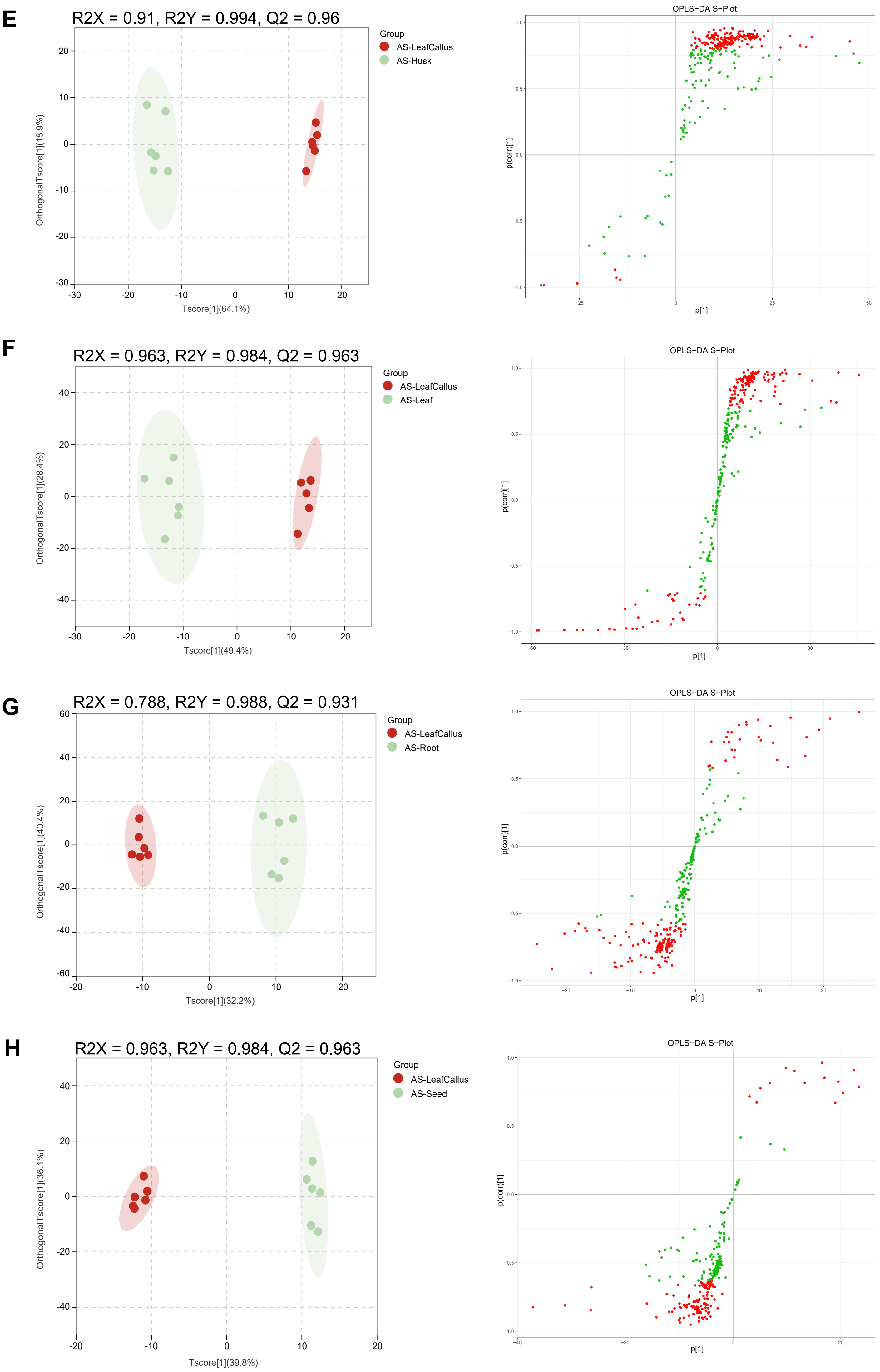
E
R2X = 0.91, R2Y = 0.994, Q2 = 0.96
Group
AS-LeafCallus
AS-Husk
OrthogonalTscore[1](18.9%)
Tscore[1](64.1%)
OPLS−DA S−Plot
p(corr)[1]
p[1]
F
R2X = 0.963, R2Y = 0.984, Q2 = 0.963
Group
AS-LeafCallus
AS-Leaf
OrthogonalTscore[1](28.4%)
Tscore[1](49.4%)
OPLS−DA S−Plot
p(corr)[1]
p[1]
G
R2X = 0.788, R2Y = 0.988, Q2 = 0.931
Group
AS-LeafCallus
AS-Root
OrthogonalTscore[1](40.4%)
Tscore[1](32.2%)
OPLS−DA S−Plot
p(corr)[1]
p[1]
H
R2X = 0.963, R2Y = 0.984, Q2 = 0.963
Group
AS-LeafCallus
AS-Seed
OrthogonalTscore[1](36.1%)
Tscore[1](39.8%)
OPLS−DA S−Plot
p(corr)[1]
p[1]

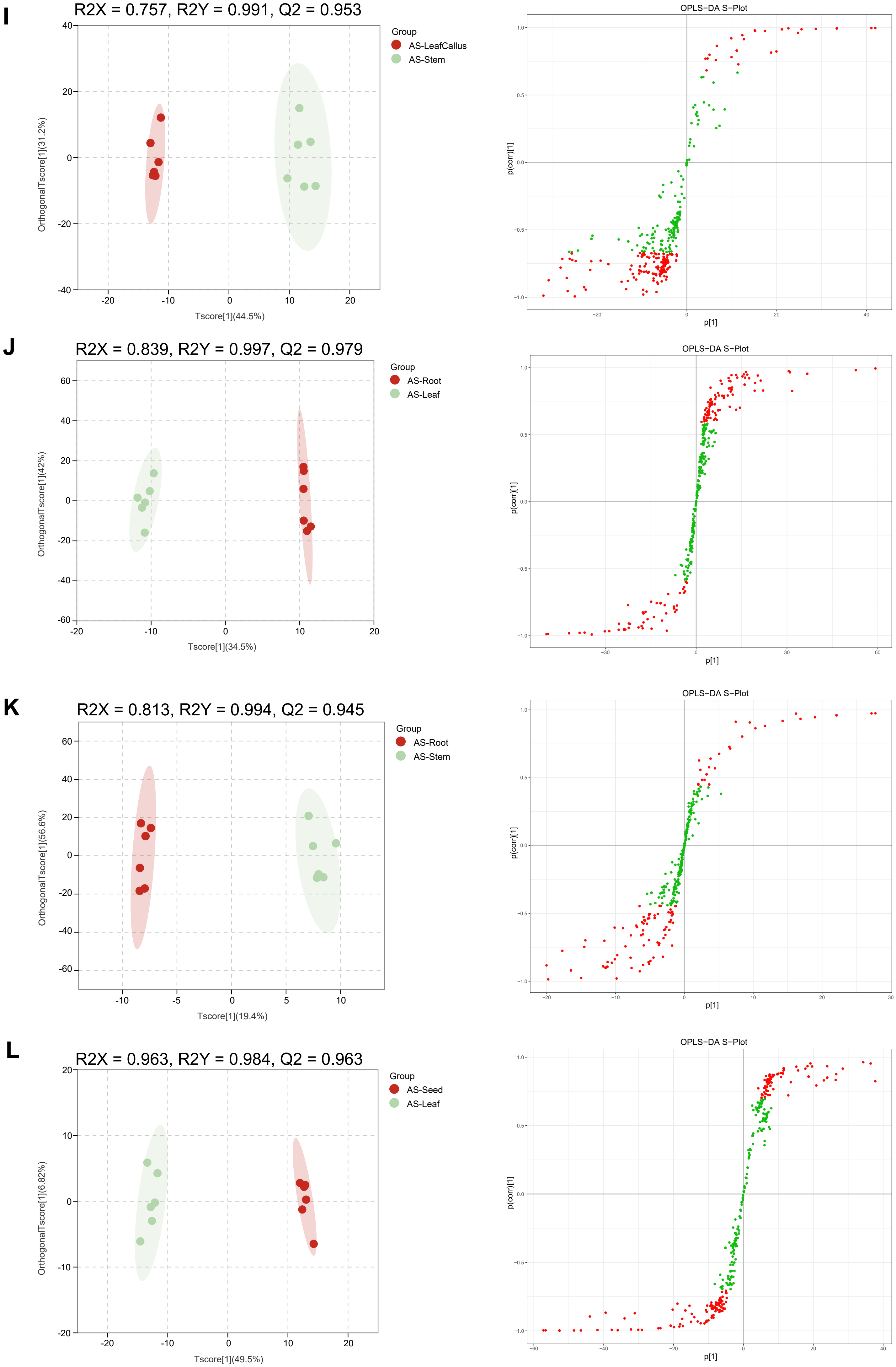

I
R2X = 0.757, R2Y = 0.991, Q2 = 0.953
Group
AS-LeafCallus
AS-Stem
OrthogonalTscore[1](31.2%)
Tscore[1](44.5%)
OPLS−DA S−Plot
p(corr)[1]
p[1]
J
R2X = 0.839, R2Y = 0.997, Q2 = 0.979
Group
AS-Root
AS-Leaf
OrthogonalTscore[1](42%)
Tscore[1](34.5%)
OPLS−DA S−Plot
p(corr)[1]
p[1]
K
R2X = 0.813, R2Y = 0.994, Q2 = 0.945
Group
AS-Root
AS-Stem
OrthogonalTscore[1](56.6%)
Tscore[1](19.4%)
OPLS−DA S−Plot
p(corr)[1]
p[1]
L
R2X = 0.963, R2Y = 0.984, Q2 = 0.963
Group
AS-Seed
AS-Leaf
OrthogonalTscore[1](6.82%)
Tscore[1](49.5%)
OPLS−DA S−Plot
p(corr)[1]
p[1]

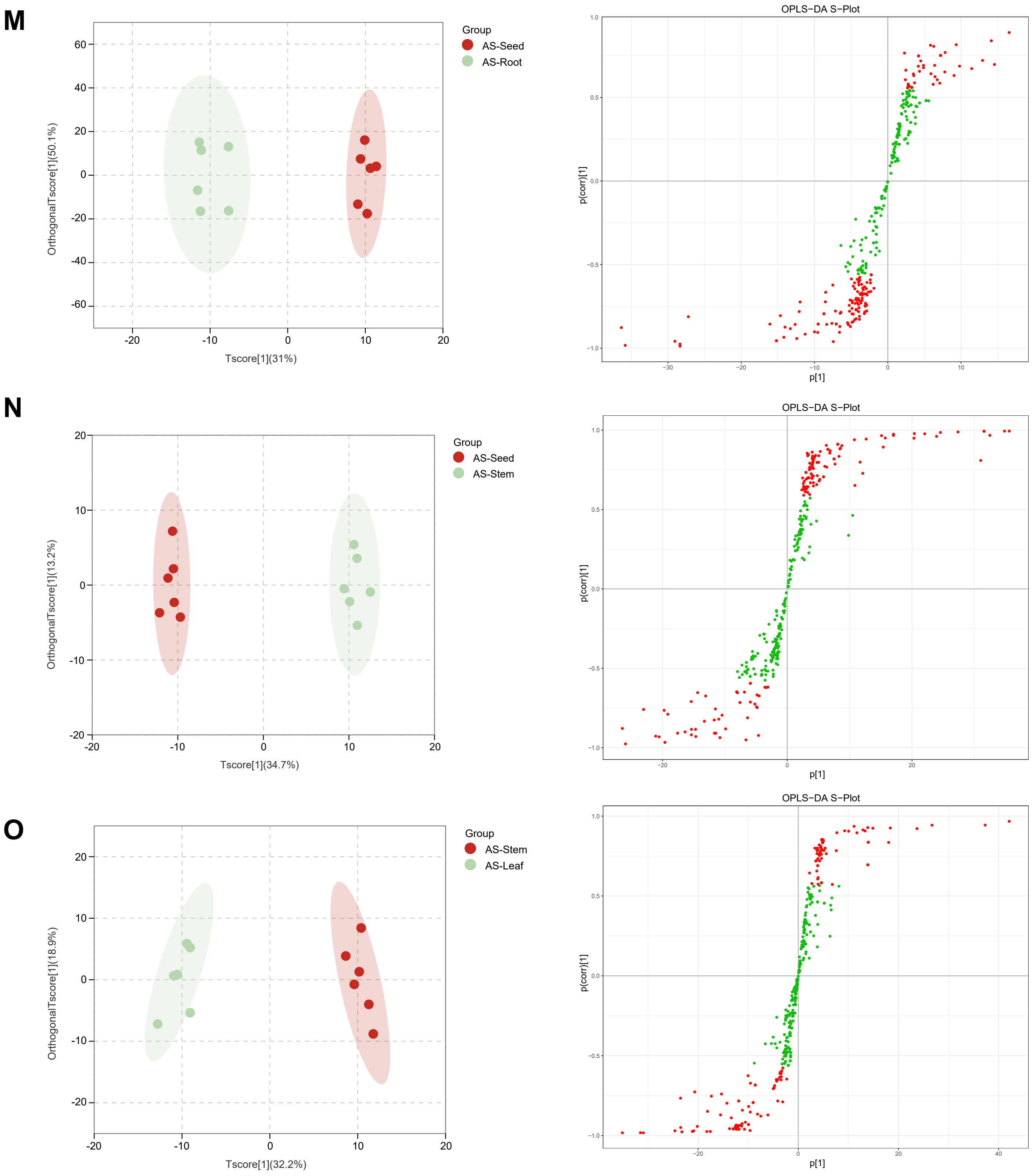

Supplementary Figure S2. Orthogonal partial least squares-discriminant analysis (OPLS-DA) scores and OPLS-DA S-plot models between six different tissue sites. (A) AS-Husk_vs_AS-Leaf, (B) AS-Husk_vs_AS-Root, (C) AS- Husk_vs_AS-Seed, (D) AS-Husk_vs_AS-Stem,(E) AS-LeafCallus_vs_AS-Husk,(F) AS-LeafCallus_vs_AS-Leaf,(G) AS-LeafCallus_vs_AS-Root, (H) AS-LeafCallus_vs_AS-Seed, (I) AS-LeafCallus_vs_AS-St--em,(G) AS-Root_vs_AS-Leaf, (K) AS-Root_vs_AS-Stem, (L) AS-Seed_vs_AS-Leaf, (M) AS-Seed_vs_AS-Root, (N) AS-Seed_vs_AS-Stem, (O) AS-Stem_vs_AS-Leaf.
