# Supplementary material for: Comparative Analysis of Metabolome and Transcriptome in Different Tissue Sites of Aquilaria sinensis (Lour.) Gilg
Source: Molecules. 2024 Feb 29;29(5):1075. doi: 10.3390/molecules29051075 (PMC10935121; doi:10.3390/molecules29051075)
Supplement: Supplementary file 1 [file molecules-29-01075-s001.zip › Supplementary Figure S3.pdf]

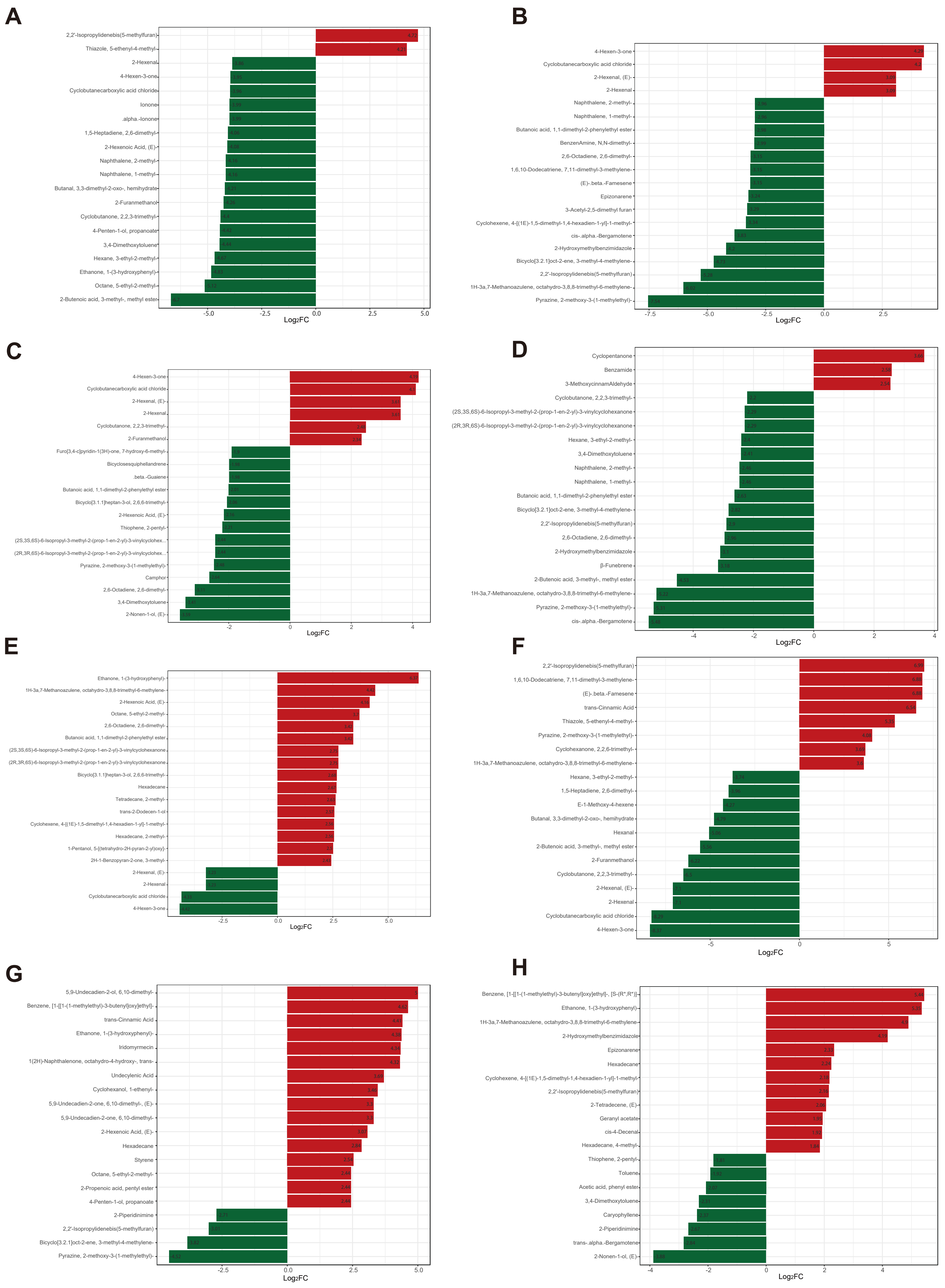
A
2,2'-Isopropylidenebis(5-methylfuran)
Thiazole, 5-ethenyl-4-methyl-
2-Hexenal
4-Hexen-3-one
Cyclobutanecarboxylic acid chloride
Ionone
.alpha.-Ionone
1,5-Heptadiene, 2,6-dimethyl-
2-Hexenoic Acid, (E)-
Naphthalene, 2-methyl-
Naphthalene, 1-methyl-
Butanal, 3,3-dimethyl-2-oxo-, hemihydrate
2-Furanmethanol
Cyclobutanone, 2,2,3-trimethyl-
4-Penten-1-ol, propanoate
3,4-Dimethoxytoluene
Hexane, 3-ethyl-2-methyl-
Ethanone, 1-(3-hydroxyphenyl)-
Octane, 5-ethyl-2-methyl-
2-Butenoic acid, 3-methyl-, methyl ester
-5.0
-2.5
0.0
2.5
5.0
Log2FC
B
4-Hexen-3-one
Cyclobutanecarboxylic acid chloride
2-Hexenal, (E)-
2-Hexenal
Naphthalene, 2-methyl-
Naphthalene, 1-methyl-
Butanoic acid, 1,1-dimethyl-2-phenylethyl ester
BenzenAmine, N,N-dimethyl-
2,6-Octadiene, 2,6-dimethyl-
1,6,10-Dodecatriene, 7,11-dimethyl-3-methylene-
(E)-.beta.-Famesene
Epizonarene
3-Acetyl-2,5-dimethyl furan
Cyclohexene, 4-[(1E)-1,5-dimethyl-1,4-hexadien-1-yl]-1-methyl-
cis-.alpha.-Bergamotene
2-Hydroxymethylbenzimidazole
Bicyclo[3.2.1]oct-2-ene, 3-methyl-4-methylene-
2,2'-Isopropylidenebis(5-methylfuran)
1H-3a,7-Methanoazulene, octahydro-3,8,8-trimethyl-6-methylene-
Pyrazine, 2-methoxy-3-(1-methylethyl)-
-7.5
-5.0
-2.5
0.0
2.5
Log2FC
C
4-Hexen-3-one
Cyclobutanecarboxylic acid chloride
2-Hexenal, (E)-
2-Hexenal
Cyclobutanone, 2,2,3-trimethyl-
2-Furanmethanol
Furo[3,4-c]pyridin-1(3H)-one, 7-hydroxy-6-methyl-
Bicyclosesquiphellandrene
.beta.-Guaiene
Butanoic acid, 1,1-dimethyl-2-phenylethyl ester
Bicyclo[3.1.1]heptan-3-ol, 2,6,6-trimethyl-
2-Hexenoic Acid, (E)-
Thiophene, 2-pentyl-
(2S,3S,6S)-6-Isopropyl-3-methyl-2-(prop-1-en-2-yl)-3-vinylcyclohex...
(2R,3R,6S)-6-Isopropyl-3-methyl-2-(prop-1-en-2-yl)-3-vinylcyclohex...
Pyrazine, 2-methoxy-3-(1-methylethyl)-
Camphor
2,6-Octadiene, 2,6-dimethyl-
3,4-Dimethoxytoluene
2-Nonen-1-ol, (E)-
-2
0
2
4
Log2FC
D
Cyclopentanone
Benzamide
3-MethoxycinnamAldehyde
Cyclobutanone, 2,2,3-trimethyl-
(2S,3S,6S)-6-Isopropyl-3-methyl-2-(prop-1-en-2-yl)-3-vinylcyclohexanone
(2R,3R,6S)-6-Isopropyl-3-methyl-2-(prop-1-en-2-yl)-3-vinylcyclohexanone
Hexane, 3-ethyl-2-methyl-
3,4-Dimethoxytoluene
Naphthalene, 2-methyl-
Naphthalene, 1-methyl-
Butanoic acid, 1,1-dimethyl-2-phenylethyl ester
Bicyclo[3.2.1]oct-2-ene, 3-methyl-4-methylene-
2,2'-Isopropylidenebis(5-methylfuran)
2,6-Octadiene, 2,6-dimethyl-
2-Hydroxymethylbenzimidazole
β-Funebrene
2-Butenoic acid, 3-methyl-, methyl ester
1H-3a,7-Methanoazulene, octahydro-3,8,8-trimethyl-6-methylene-
Pyrazine, 2-methoxy-3-(1-methylethyl)-
cis-.alpha.-Bergamotene
-4
-2
0
2
4
Log2FC
E
Ethanone, 1-(3-hydroxyphenyl)-
1H-3a,7-Methanoazulene, octahydro-3,8,8-trimethyl-6-methylene-
2-Hexenoic Acid, (E)-
Octane, 5-ethyl-2-methyl-
2,6-Octadiene, 2,6-dimethyl-
Butanoic acid, 1,1-dimethyl-2-phenylethyl ester
(2S,3S,6S)-6-Isopropyl-3-methyl-2-(prop-1-en-2-yl)-3-vinylcyclohexanone
(2R,3R,6S)-6-Isopropyl-3-methyl-2-(prop-1-en-2-yl)-3-vinylcyclohexanone
Bicyclo[3.1.1]heptan-3-ol, 2,6,6-trimethyl-
Hexadecane
Tetradecane, 2-methyl-
trans-2-Dodecen-1-ol
Cyclohexene, 4-[(1E)-1,5-dimethyl-1,4-hexadien-1-yl]-1-methyl-
Hexadecane, 2-methyl-
1-Pentanol, 5-[(tetrahydro-2H-pyran-2-yl)oxy]-
2H-1-Benzopyran-2-one, 3-methyl-
2-Hexenal, (E)-
2-Hexenal
Cyclobutanecarboxylic acid chloride
4-Hexen-3-one
-2.5
0.0
2.5
5.0
Log2FC
F
2,2'-Isopropylidenebis(5-methylfuran)
1,6,10-Dodecatriene, 7,11-dimethyl-3-methylene-
(E)-.beta.-Famesene
trans-Cinnamic Acid
Thiazole, 5-ethenyl-4-methyl-
Pyrazine, 2-methoxy-3-(1-methylethyl)-
Cyclohexanone, 2,2,6-trimethyl-
1H-3a,7-Methanoazulene, octahydro-3,8,8-trimethyl-6-methylene-
Hexane, 3-ethyl-2-methyl-
1,5-Heptadiene, 2,6-dimethyl-
E-1-Methoxy-4-hexene
Butanal, 3,3-dimethyl-2-oxo-, hemihydrate
Hexanal
2-Butenoic acid, 3-methyl-, methyl ester
2-Furanmethanol
Cyclobutanone, 2,2,3-trimethyl-
2-Hexenal, (E)-
2-Hexenal
Cyclobutanecarboxylic acid chloride
4-Hexen-3-one
-5
0
5
Log2FC
G
5,9-Undecadien-2-ol, 6,10-dimethyl-
Benzene, [1-[[1-(1-methylethyl)-3-butenyl]oxy]ethyl]-
trans-Cinnamic Acid
Ethanone, 1-(3-hydroxyphenyl)-
Iridomyrmecin
1(2H)-Naphthalenone, octahydro-4-hydroxy-, trans-
Undecylenic Acid
Cyclohexanol, 1-ethenyl-
5,9-Undecadien-2-one, 6,10-dimethyl-, (E)-
5,9-Undecadien-2-one, 6,10-dimethyl-
2-Hexenoic Acid, (E)-
Hexadecane
Styrene
Octane, 5-ethyl-2-methyl-
2-Propenoic acid, pentyl ester
4-Penten-1-ol, propanoate
2-Piperidinimine
2,2'-Isopropylidenebis(5-methylfuran)
Bicyclo[3.2.1]oct-2-ene, 3-methyl-4-methylene-
Pyrazine, 2-methoxy-3-(1-methylethyl)-
-2.5
0.0
2.5
5.0
Log2FC
H
Benzene, [1-[[1-(1-methylethyl)-3-butenyl]oxy]ethyl]-, [S-(R*,R*)]-
Ethanone, 1-(3-hydroxyphenyl)-
1H-3a,7-Methanoazulene, octahydro-3,8,8-trimethyl-6-methylene-
2-Hydroxymethylbenzimidazole
Epizonarene
Hexadecane
Cyclohexene, 4-[(1E)-1,5-dimethyl-1,4-hexadien-1-yl]-1-methyl-
2,2'-Isopropylidenebis(5-methylfuran)
2-Tetradecene, (E)-
Geranyl acetate
cis-4-Decenal
Hexadecane, 4-methyl-
Thiophene, 2-pentyl-
Toluene
Acetic acid, phenyl ester
3,4-Dimethoxytoluene
Caryophyllene
2-Piperidinimine
trans-.alpha.-Bergamotene
2-Nonen-1-ol, (E)-
-4
-2
0
2
4
Log2FC

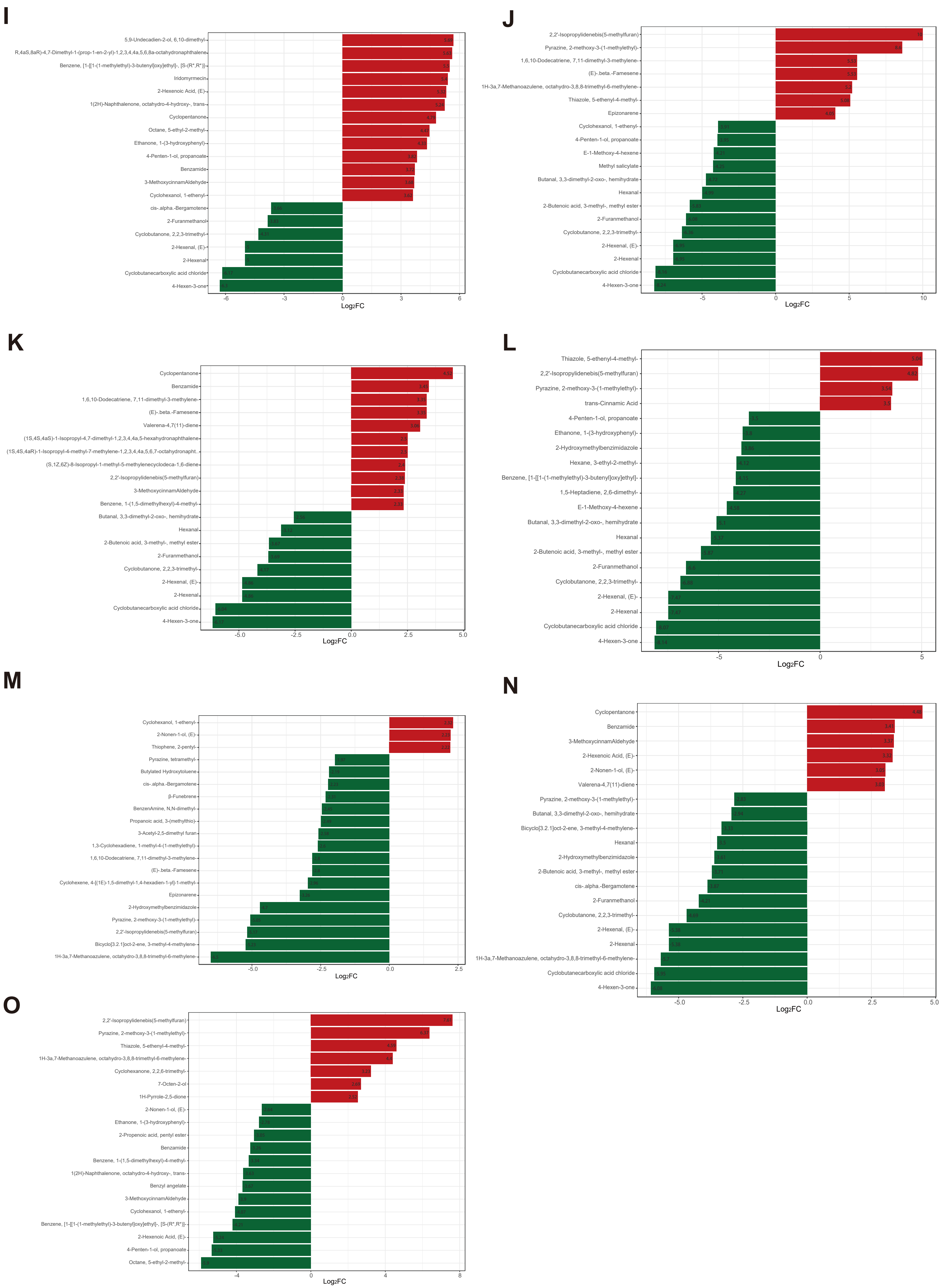

I
5,9-Undecadien-2-ol, 6,10-dimethyl-
R,4aS,8aR)-4,7-Dimethyl-1-(prop-1-en-2-yl)-1,2,3,4,4a,5,6,8a-octahydronaphthalene
Benzene, [1-[[1-(1-methylethyl)-3-butenyl]oxy]ethyl]-, [S-(R*,R*)]-
Iridomyrmecin
2-Hexenoic Acid, (E)-
1(2H)-Naphthalenone, octahydro-4-hydroxy-, trans-
Cyclopentanone
Octane, 5-ethyl-2-methyl-
Ethanone, 1-(3-hydroxyphenyl)-
4-Penten-1-ol, propanoate
Benzamide
3-MethoxycinnamAldehyde
Cyclohexanol, 1-ethenyl-
cis-.alpha.-Bergamotene
2-Furanmethanol
Cyclobutanone, 2,2,3-trimethyl-
2-Hexenal, (E)-
2-Hexenal
Cyclobutanecarboxylic acid chloride
4-Hexen-3-one
-6
-3
0
3
6
Log2FC
J
2,2'-Isopropylidenebis(5-methylfuran)
Pyrazine, 2-methoxy-3-(1-methylethyl)-
1,6,10-Dodecatriene, 7,11-dimethyl-3-methylene-
(E)-.beta.-Famesene
1H-3a,7-Methanoazulene, octahydro-3,8,8-trimethyl-6-methylene-
Thiazole, 5-ethenyl-4-methyl-
Epizonarene
Cyclohexanol, 1-ethenyl-
4-Penten-1-ol, propanoate
E-1-Methoxy-4-hexene
Methyl salicylate
Butanal, 3,3-dimethyl-2-oxo-, hemihydrate
Hexanal
2-Butenoic acid, 3-methyl-, methyl ester
2-Furanmethanol
Cyclobutanone, 2,2,3-trimethyl-
2-Hexenal, (E)-
2-Hexenal
Cyclobutanecarboxylic acid chloride
4-Hexen-3-one
-5
0
5
10
Log2FC
K
Cyclopentanone
Benzamide
1,6,10-Dodecatriene, 7,11-dimethyl-3-methylene-
(E)-.beta.-Famesene
Valerena-4,7(11)-diene
(1S,4S,4aS)-1-Isopropyl-4,7-dimethyl-1,2,3,4,4a,5-hexahydronaphthalene
(1S,4S,4aR)-1-Isopropyl-4-methyl-7-methylene-1,2,3,4,4a,5,6,7-octahydronapht..
(S,1Z,6Z)-8-Isopropyl-1-methyl-5-methylenecyclodeca-1,6-diene
2,2'-Isopropylidenebis(5-methylfuran)
3-MethoxycinnamAldehyde
Benzene, 1-(1,5-dimethylhexyl)-4-methyl-
Butanal, 3,3-dimethyl-2-oxo-, hemihydrate
Hexanal
2-Butenoic acid, 3-methyl-, methyl ester
2-Furanmethanol
Cyclobutanone, 2,2,3-trimethyl-
2-Hexenal, (E)-
2-Hexenal
Cyclobutanecarboxylic acid chloride
4-Hexen-3-one
-5.0
-2.5
0.0
2.5
5.0
Log2FC
L
Thiazole, 5-ethenyl-4-methyl-
2,2'-Isopropylidenebis(5-methylfuran)
Pyrazine, 2-methoxy-3-(1-methylethyl)-
trans-Cinnamic Acid
4-Penten-1-ol, propanoate
Ethanone, 1-(3-hydroxyphenyl)-
2-Hydroxymethylbenzimidazole
Hexane, 3-ethyl-2-methyl-
Benzene, [1-[[1-(1-methylethyl)-3-butenyl]oxy]ethyl]-
1,5-Heptadiene, 2,6-dimethyl-
E-1-Methoxy-4-hexene
Butanal, 3,3-dimethyl-2-oxo-, hemihydrate
Hexanal
2-Butenoic acid, 3-methyl-, methyl ester
2-Furanmethanol
Cyclobutanone, 2,2,3-trimethyl-
2-Hexenal, (E)-
2-Hexenal
Cyclobutanecarboxylic acid chloride
4-Hexen-3-one
-5
0
5
Log2FC
M
Cyclohexanol, 1-ethenyl-
2-Nonen-1-ol, (E)-
Thiophene, 2-pentyl-
Pyrazine, tetramethyl-
Butylated Hydroxytoluene
cis-.alpha.-Bergamotene
β-Funebrene
BenzenAmine, N,N-dimethyl-
Propanoic acid, 3-(methylthio)-
3-Acetyl-2,5-dimethyl furan
1,3-Cyclohexadiene, 1-methyl-4-(1-methylethyl)-
1,6,10-Dodecatriene, 7,11-dimethyl-3-methylene-
(E)-.beta.-Famesene
Cyclohexene, 4-[(1E)-1,5-dimethyl-1,4-hexadien-1-yl]-1-methyl-
Epizonarene
2-Hydroxymethylbenzimidazole
Pyrazine, 2-methoxy-3-(1-methylethyl)-
2,2'-Isopropylidenebis(5-methylfuran)
Bicyclo[3.2.1]oct-2-ene, 3-methyl-4-methylene-
1H-3a,7-Methanoazulene, octahydro-3,8,8-trimethyl-6-methylene-
-5.0
-2.5
0.0
2.5
Log2FC
N
Cyclopentanone
Benzamide
3-MethoxycinnamAldehyde
2-Hexenoic Acid, (E)-
2-Nonen-1-ol, (E)-
Valerena-4,7(11)-diene
Pyrazine, 2-methoxy-3-(1-methylethyl)-
Butanal, 3,3-dimethyl-2-oxo-, hemihydrate
Bicyclo[3.2.1]oct-2-ene, 3-methyl-4-methylene-
Hexanal
2-Hydroxymethylbenzimidazole
2-Butenoic acid, 3-methyl-, methyl ester
cis-.alpha.-Bergamotene
2-Furanmethanol
Cyclobutanone, 2,2,3-trimethyl-
2-Hexenal, (E)-
2-Hexenal
1H-3a,7-Methanoazulene, octahydro-3,8,8-trimethyl-6-methylene-
Cyclobutanecarboxylic acid chloride
4-Hexen-3-one
-5.0
-2.5
0.0
2.5
5.0
Log2FC
O
2,2'-Isopropylidenebis(5-methylfuran)
Pyrazine, 2-methoxy-3-(1-methylethyl)-
Thiazole, 5-ethenyl-4-methyl-
1H-3a,7-Methanoazulene, octahydro-3,8,8-trimethyl-6-methylene-
Cyclohexanone, 2,2,6-trimethyl-
7-Octen-2-ol
1H-Pyrrole-2,5-dione
2-Nonen-1-ol, (E)-
Ethanone, 1-(3-hydroxyphenyl)-
2-Propenoic acid, pentyl ester
Benzamide
Benzene, 1-(1,5-dimethylhexyl)-4-methyl-
1(2H)-Naphthalenone, octahydro-4-hydroxy-, trans-
Benzyl angelate
3-MethoxycinnamAldehyde
Cyclohexanol, 1-ethenyl-
Benzene, [1-[[1-(1-methylethyl)-3-butenyl]oxy]ethyl]-, [S-(R*,R*)]-
2-Hexenoic Acid, (E)-
4-Penten-1-ol, propanoate
Octane, 5-ethyl-2-methyl-
-4
0
4
8
Log2FC

Supplementary Figure S3. Bar Chart of DAMs between each comparison group (Top 20). (A) AS-Husk_vs_AS-Leaf, (B) AS-Husk_vs_AS-Root, (C) AS-Husk_vs_AS-Seed, (D) AS-Husk_vs_AS-Stem,(E) AS-LeafCallus_vs_AS-Husk,(F) AS-LeafCallus_vs_AS-Leaf,(G) AS-LeafCallus_vs_AS-Root,(H) AS-LeafCallus_vs_AS-Seed, (I) AS-LeafCallus_vs_AS-Stem, (G) AS-Root_vs_AS-Leaf, (K) AS-Root_vs_AS-Stem, (L) AS-Seed_vs_AS-Leaf, (M) AS-Seed_vs_AS-Root, (N) AS-Seed_vs_AS-Stem, (O) AS-Stem_vs_AS-Leaf.
