# Supplementary material for: Comparative Analysis of Metabolome and Transcriptome in Different Tissue Sites of Aquilaria sinensis (Lour.) Gilg
Source: Molecules. 2024 Feb 29;29(5):1075. doi: 10.3390/molecules29051075 (PMC10935121; doi:10.3390/molecules29051075)
Supplement: Supplementary file 1 [file molecules-29-01075-s001.zip › Supplementary Figure S4.pdf]

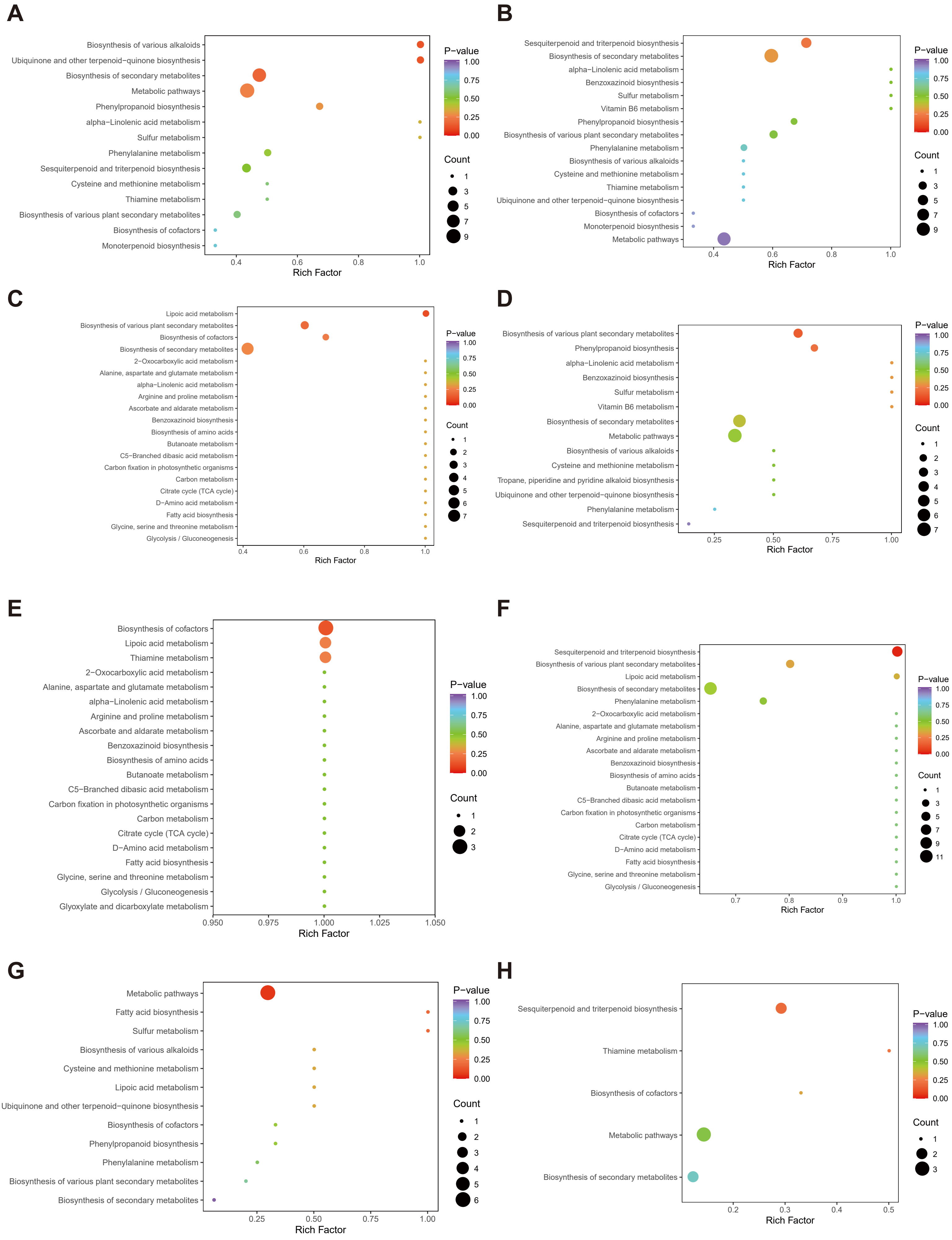
A
Biosynthesis of various alkaloids
Ubiquinone and other terpenoid−quinone biosynthesis
Biosynthesis of secondary metabolites
Metabolic pathways
Phenylpropanoid biosynthesis
alpha−Linolenic acid metabolism
Sulfur metabolism
Phenylalanine metabolism
Sesquiterpenoid and triterpenoid biosynthesis
Cysteine and methionine metabolism
Thiamine metabolism
Biosynthesis of various plant secondary metabolites
Biosynthesis of cofactors
Monoterpenoid biosynthesis
0.4 0.6 0.8 1.0
Rich Factor
P−value
1.00 0.75 0.50 0.25 0.00
Count
1 3 5 7 9
B
Sesquiterpenoid and triterpenoid biosynthesis
Biosynthesis of secondary metabolites
alpha−Linolenic acid metabolism
Benzoxazinoid biosynthesis
Sulfur metabolism
Vitamin B6 metabolism
Phenylpropanoid biosynthesis
Biosynthesis of various plant secondary metabolites
Phenylalanine metabolism
Biosynthesis of various alkaloids
Cysteine and methionine metabolism
Thiamine metabolism
Ubiquinone and other terpenoid−quinone biosynthesis
Biosynthesis of cofactors
Monoterpenoid biosynthesis
Metabolic pathways
0.4 0.6 0.8 1.0
Rich Factor
P−value
1.00 0.75 0.50 0.25 0.00
Count
1 3 5 7 9
C
Lipoic acid metabolism
Biosynthesis of various plant secondary metabolites
Biosynthesis of cofactors
Biosynthesis of secondary metabolites
2−Oxocarboxylic acid metabolism
Alanine, aspartate and glutamate metabolism
alpha−Linolenic acid metabolism
Arginine and proline metabolism
Ascorbate and aldarate metabolism
Benzoxazinoid biosynthesis
Biosynthesis of amino acids
Butanoate metabolism
C5−Branched dibasic acid metabolism
Carbon fixation in photosynthetic organisms
Carbon metabolism
Citrate cycle (TCA cycle)
D−Amino acid metabolism
Fatty acid biosynthesis
Glycine, serine and threonine metabolism
Glycolysis / Gluconeogenesis
0.4 0.6 0.8 1.0
Rich Factor
P−value
1.00 0.75 0.50 0.25 0.00
Count
1 2 3 4 5 6 7
D
Biosynthesis of various plant secondary metabolites
Phenylpropanoid biosynthesis
alpha−Linolenic acid metabolism
Benzoxazinoid biosynthesis
Sulfur metabolism
Vitamin B6 metabolism
Biosynthesis of secondary metabolites
Metabolic pathways
Biosynthesis of various alkaloids
Cysteine and methionine metabolism
Tropane, piperidine and pyridine alkaloid biosynthesis
Ubiquinone and other terpenoid−quinone biosynthesis
Phenylalanine metabolism
Sesquiterpenoid and triterpenoid biosynthesis
0.25 0.50 0.75 1.00
Rich Factor
P−value
1.00 0.75 0.50 0.25 0.00
Count
1 2 3 4 5 6 7
E
Biosynthesis of cofactors
Lipoic acid metabolism
Thiamine metabolism
2−Oxocarboxylic acid metabolism
Alanine, aspartate and glutamate metabolism
alpha−Linolenic acid metabolism
Arginine and proline metabolism
Ascorbate and aldarate metabolism
Benzoxazinoid biosynthesis
Biosynthesis of amino acids
Butanoate metabolism
C5−Branched dibasic acid metabolism
Carbon fixation in photosynthetic organisms
Carbon metabolism
Citrate cycle (TCA cycle)
D−Amino acid metabolism
Fatty acid biosynthesis
Glycine, serine and threonine metabolism
Glycolysis / Gluconeogenesis
Glyoxylate and dicarboxylate metabolism
0.950 0.975 1.000 1.025 1.050
Rich Factor
P−value
1.00 0.75 0.50 0.25 0.00
Count
1 2 3
F
Sesquiterpenoid and triterpenoid biosynthesis
Biosynthesis of various plant secondary metabolites
Lipoic acid metabolism
Biosynthesis of secondary metabolites
Phenylalanine metabolism
2−Oxocarboxylic acid metabolism
Alanine, aspartate and glutamate metabolism
Arginine and proline metabolism
Ascorbate and aldarate metabolism
Benzoxazinoid biosynthesis
Biosynthesis of amino acids
Butanoate metabolism
C5−Branched dibasic acid metabolism
Carbon fixation in photosynthetic organisms
Carbon metabolism
Citrate cycle (TCA cycle)
D−Amino acid metabolism
Fatty acid biosynthesis
Glycine, serine and threonine metabolism
Glycolysis / Gluconeogenesis
0.7 0.8 0.9 1.0
Rich Factor
P−value
1.00 0.75 0.50 0.25 0.00
Count
1 3 5 7 9 11
G
Metabolic pathways
Fatty acid biosynthesis
Sulfur metabolism
Biosynthesis of various alkaloids
Cysteine and methionine metabolism
Lipoic acid metabolism
Ubiquinone and other terpenoid−quinone biosynthesis
Biosynthesis of cofactors
Phenylpropanoid biosynthesis
Phenylalanine metabolism
Biosynthesis of various plant secondary metabolites
Biosynthesis of secondary metabolites
0.25 0.50 0.75 1.00
Rich Factor
P−value
1.00 0.75 0.50 0.25 0.00
Count
1 2 3 4 5 6
H
Sesquiterpenoid and triterpenoid biosynthesis
Thiamine metabolism
Biosynthesis of cofactors
Metabolic pathways
Biosynthesis of secondary metabolites
0.2 0.3 0.4 0.5
Rich Factor
P−value
1.00 0.75 0.50 0.25 0.00
Count
1 2 3

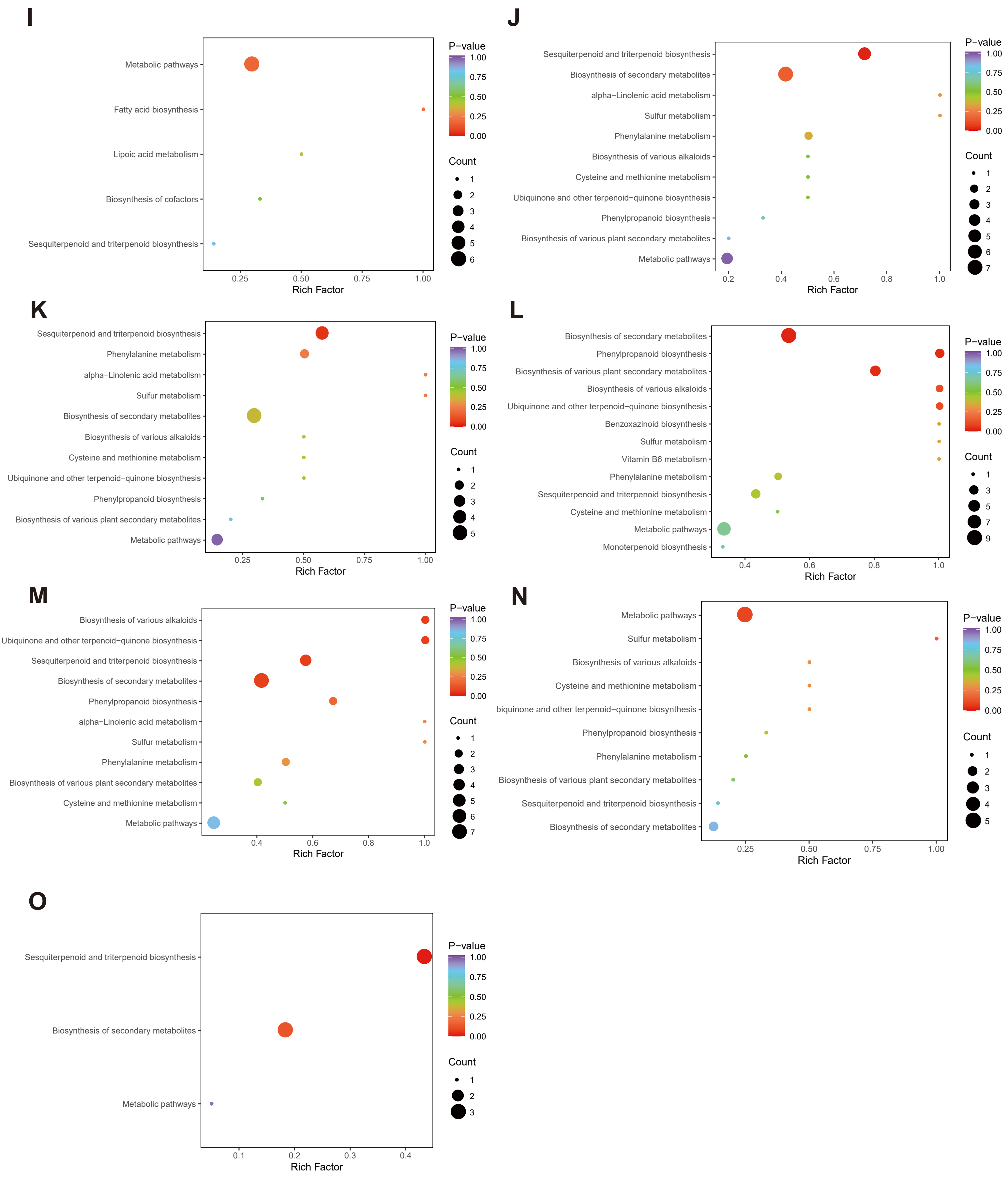


Supplementary Figure S4. KEGG enrichment of DAMs between each comparison group. (A) AS- Husk_vs_AS-Leaf, (B) AS-Husk_vs_AS-Root, (C) AS-Husk_vs_AS-Seed, (D) AS-Husk_vs_AS-Stem,(E) AS-LeafCallus_vs_AS-Husk,(F) AS-LeafCallus_vs_AS-Leaf,(G) AS-LeafCallus_vs_AS-Root,(H) AS-LeafCallus_vs_AS-Seed, (I) AS-LeafCallus_vs_AS-Stem, (J) AS-Root_vs_AS-Leaf, (K) AS-Root_vs_AS-Stem, (L) AS-Seed_vs_AS-Leaf, (M) AS-Seed_vs_AS-Root, (N) AS-Seed_vs_AS-Stem, (O) AS-Stem_vs_AS-Leaf.
