# Supplementary material for: Comparative Analysis of Metabolome and Transcriptome in Different Tissue Sites of Aquilaria sinensis (Lour.) Gilg
Source: Molecules. 2024 Feb 29;29(5):1075. doi: 10.3390/molecules29051075 (PMC10935121; doi:10.3390/molecules29051075)
Supplement: Supplementary file 1 [file molecules-29-01075-s001.zip › Supplementary Figure S5.pdf]

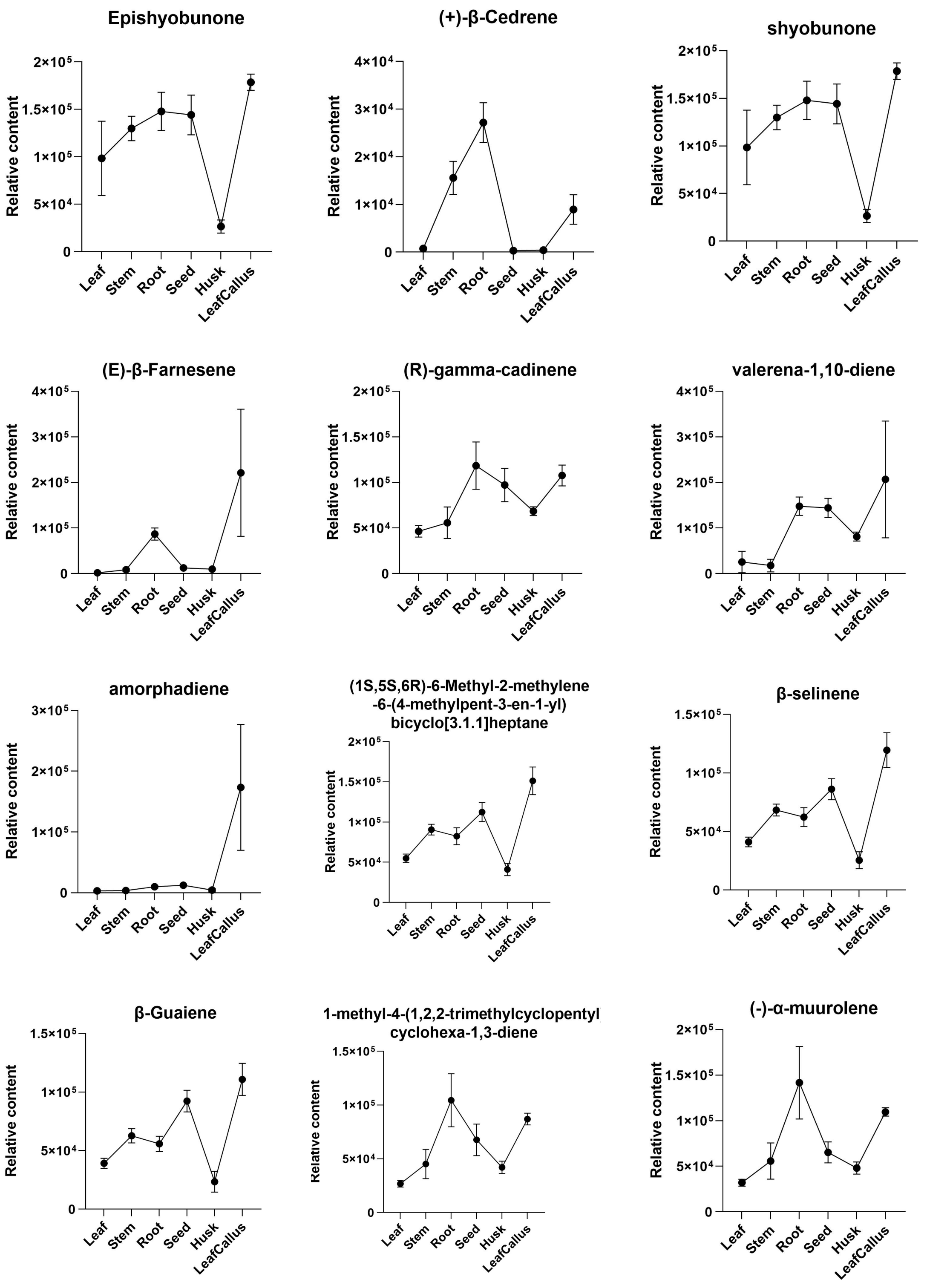
Epishyobunone
(+)-β-Cedrene
shyobunone
(E)-β-Farnesene
(R)-gamma-cadinene
valerena-1,10-diene
amorphadiene
(1S,5S,6R)-6-Methyl-2-methylene
-6-(4-methylpent-3-en-1-yl)
bicyclo[3.1.1]heptane
β-selinene
β-Guaiene
1-methyl-4-(1,2,2-trimethylcyclopentyl
cyclohexa-1,3-diene
(-)-α-muurolene
Relative content
Leaf
Stem
Root
Seed
Husk
LeafCallus

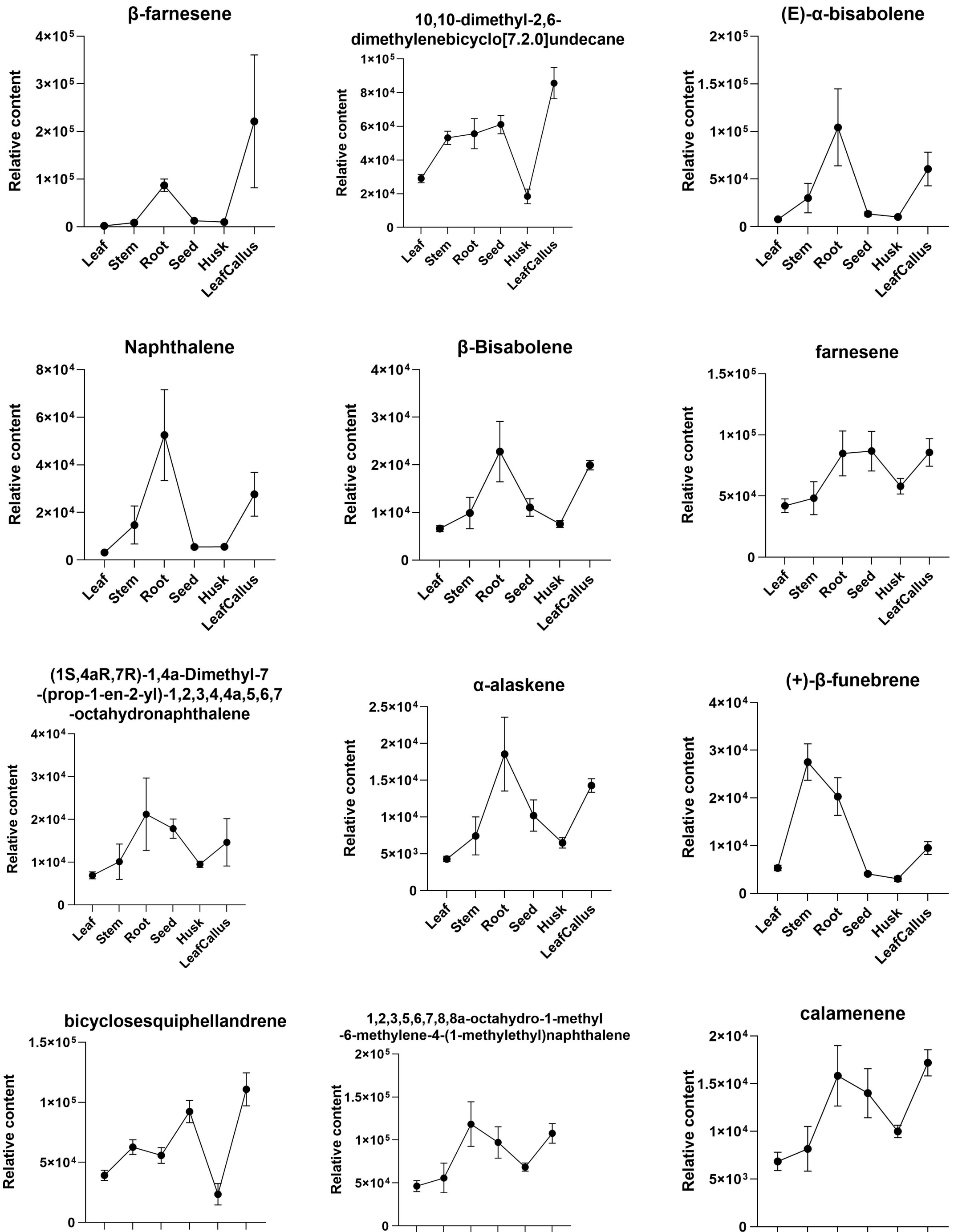
β-farnesene
10,10-dimethyl-2,6-dimethylenebicyclo[7.2.0]undecane
(E)-α-bisabolene
Naphthalene
β-Bisabolene
farnesene
(1S,4aR,7R)-1,4a-Dimethyl-7-(prop-1-en-2-yl)-1,2,3,4,4a,5,6,7-octahydronaphthalene
α-alaskene
(+)-β-funebrene
bicyclosesquiphellandrene
1,2,3,5,6,7,8,8a-octahydro-1-methyl-6-methylene-4-(1-methylethyl)naphthalene
calamenene
Relative content
Leaf
Stem
Root
Seed
Husk
LeafCallus

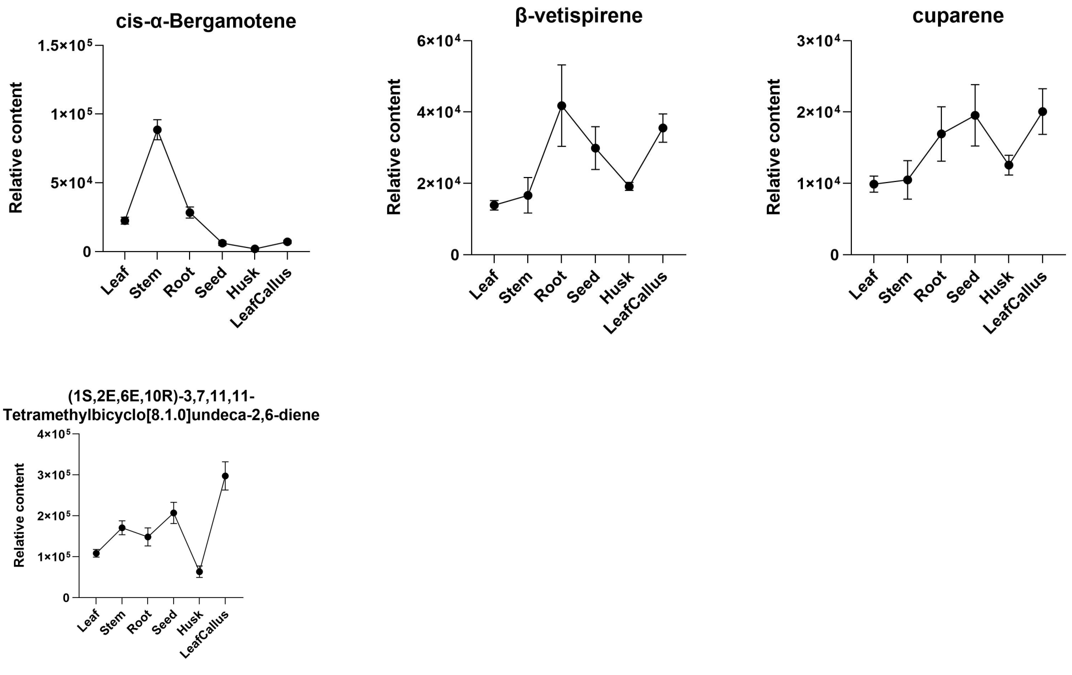

Supplementary Figure S5. Relative content of sesquiterpenes in six different tissue sites except for sesquiterpenes shown in Figure 5C. Data are expressed as mean ± standard error of the mean (SEM), n=6.
