# Supplementary material for: Comparative Analysis of Metabolome and Transcriptome in Different Tissue Sites of Aquilaria sinensis (Lour.) Gilg
Source: Molecules. 2024 Feb 29;29(5):1075. doi: 10.3390/molecules29051075 (PMC10935121; doi:10.3390/molecules29051075)
Supplement: Supplementary file 1 [file molecules-29-01075-s001.zip › Supplementary Figure S6.pdf]

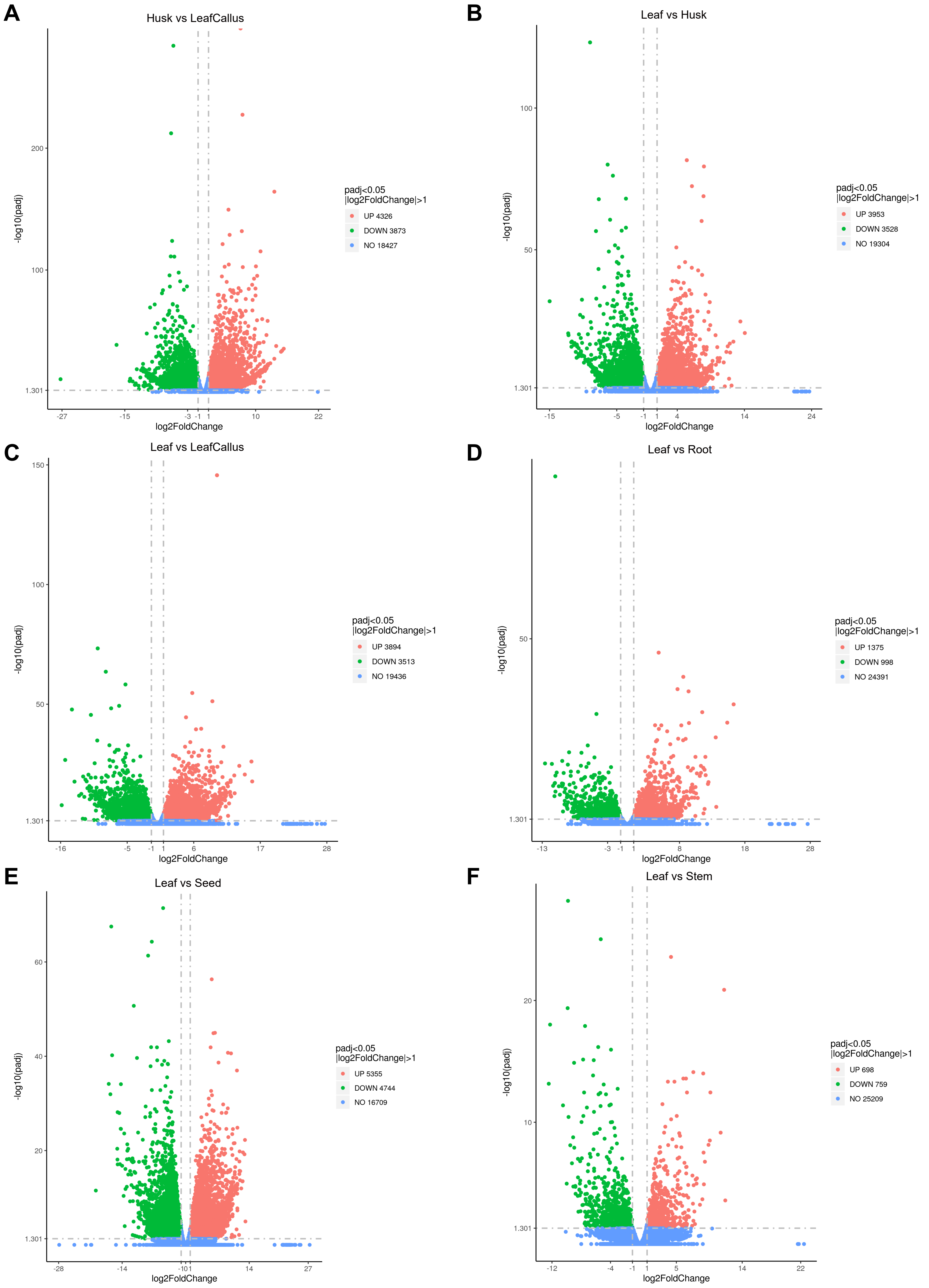

A
Husk vs LeafCallus
-log10(padj)
200
100
1.301
-27
-15
-3
-1
1
10
22
log2FoldChange
padj<0.05
|log2FoldChange|>1
UP 4326
DOWN 3873
NO 18427
B
Leaf vs Husk
-log10(padj)
100
50
1.301
-15
-5
-1
1
4
14
24
log2FoldChange
padj<0.05
|log2FoldChange|>1
UP 3953
DOWN 3528
NO 19304
C
Leaf vs LeafCallus
-log10(padj)
150
100
50
1.301
-16
-5
-1
1
6
17
28
log2FoldChange
padj<0.05
|log2FoldChange|>1
UP 3894
DOWN 3513
NO 19436
D
Leaf vs Root
-log10(padj)
50
1.301
-13
-3
-1
1
8
18
28
log2FoldChange
padj<0.05
|log2FoldChange|>1
UP 1375
DOWN 998
NO 24391
E
Leaf vs Seed
-log10(padj)
60
40
20
1.301
-28
-14
-101
14
27
log2FoldChange
padj<0.05
|log2FoldChange|>1
UP 5355
DOWN 4744
NO 16709
F
Leaf vs Stem
-log10(padj)
20
10
1.301
-12
-4
-1
1
5
14
22
log2FoldChange
padj<0.05
|log2FoldChange|>1
UP 698
DOWN 759
NO 25209

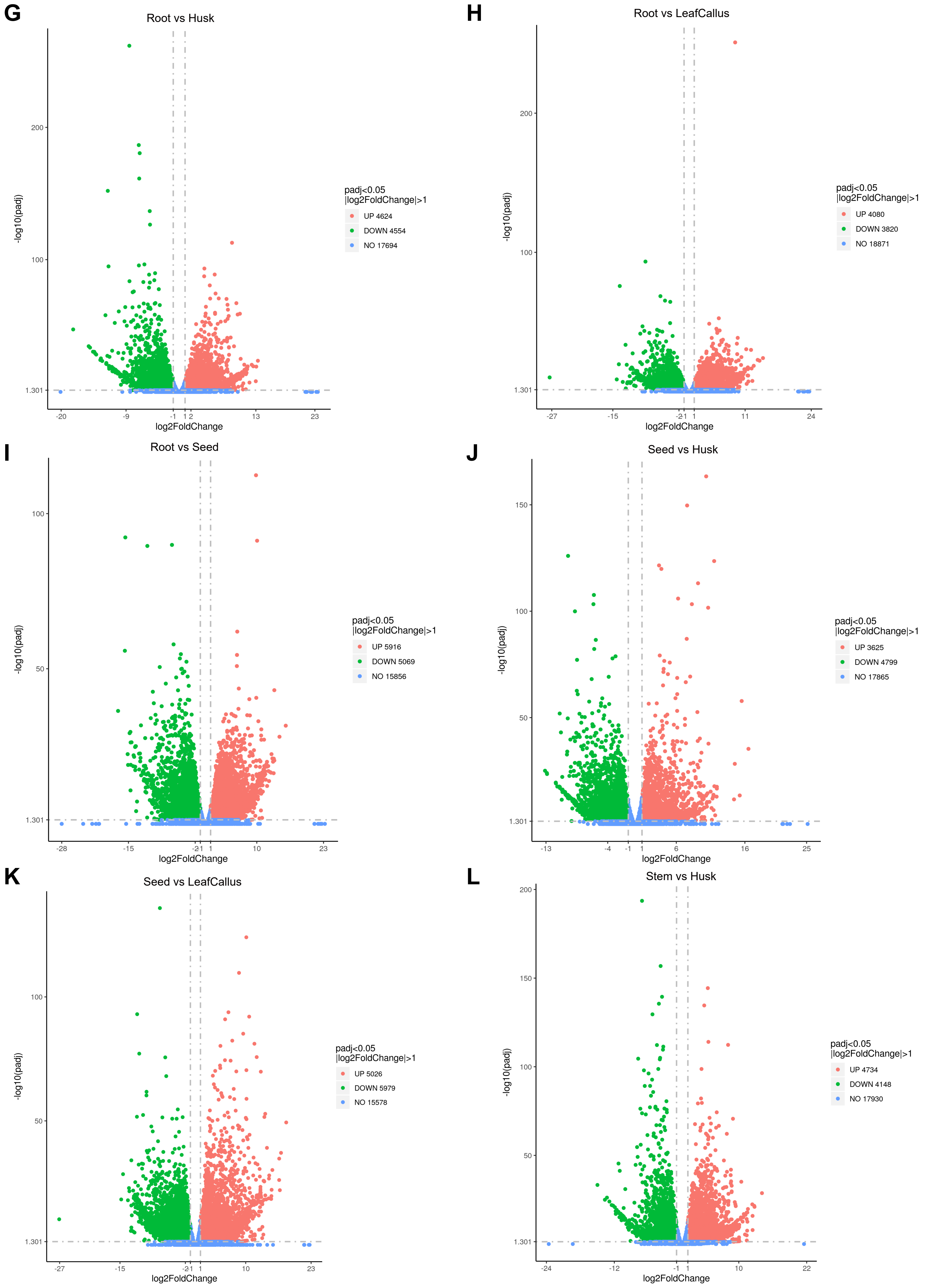
G
Root vs Husk
-log10(padj)
200
100
1.301
-20
-9
-1 1 2
13
23
log2FoldChange
padj<0.05
|log2FoldChange|>1
UP 4624
DOWN 4554
NO 17694
H
Root vs LeafCallus
-log10(padj)
200
100
1.301
-27
-15
-2 -1 1
11
24
log2FoldChange
padj<0.05
|log2FoldChange|>1
UP 4080
DOWN 3820
NO 18871
I
Root vs Seed
-log10(padj)
100
50
1.301
-28
-15
-2 -1 1
10
23
log2FoldChange
padj<0.05
|log2FoldChange|>1
UP 5916
DOWN 5069
NO 15856
J
Seed vs Husk
-log10(padj)
150
100
50
1.301
-13
-4
-1
1
6
16
25
log2FoldChange
padj<0.05
|log2FoldChange|>1
UP 3625
DOWN 4799
NO 17865
K
Seed vs LeafCallus
-log10(padj)
100
50
1.301
-27
-15
-2 -1 1
10
23
log2FoldChange
padj<0.05
|log2FoldChange|>1
UP 5026
DOWN 5979
NO 15578
L
Stem vs Husk
-log10(padj)
200
150
100
50
1.301
-24
-12
-1 1
10
22
log2FoldChange
padj<0.05
|log2FoldChange|>1
UP 4734
DOWN 4148
NO 17930

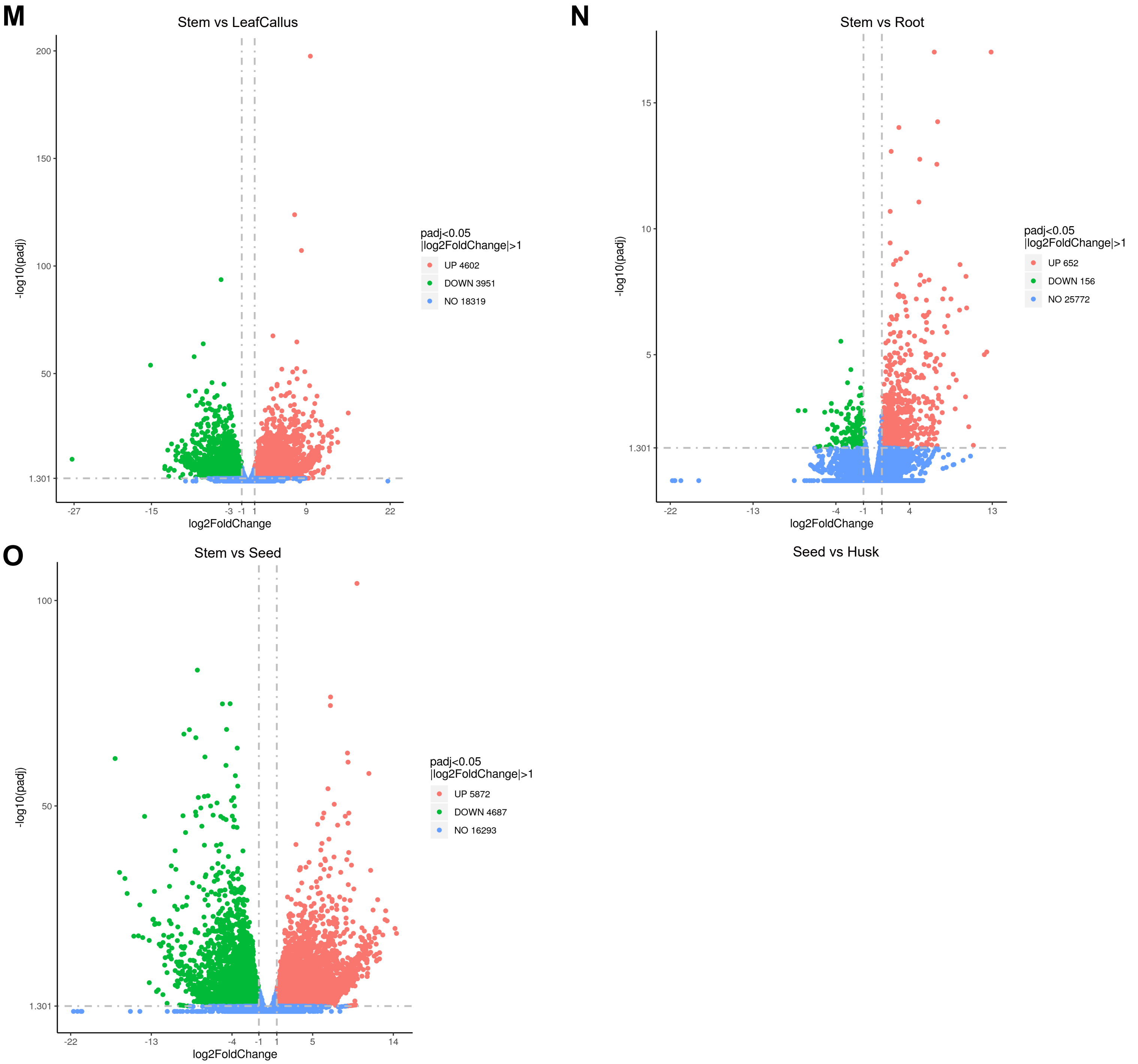

Supplementary Figure S6. Volcano plot of differentially expressed genes among six different tissue sites.(A) HuskvsLeafCallus, (B) LeafvsHusk, (C) LeafvsLeafCallus, (D)LeafvsRoot, (E) LeafvsSeed,(F) Leafvs Stem , (G) Root vsHusk, (H) RootvsLeafCallus, (I)RootvsSeed, (J) SeedvsHusk, (K)Seed vs LeafCallus,(L) Stem vsHusk, (M) StemvsLeafCallus, (N)StemvsRoot, (O) StemvsSeed. The representation of each dot corresponds to a specific gene, with red dots indicating up-regulated differentially expressed genes , green dots representing down-regulated differentially expressed genes, and blue dots denoting genes that were detected but did not exhibit significant differences.
