# Supplementary material for: Comparative Analysis of Metabolome and Transcriptome in Different Tissue Sites of Aquilaria sinensis (Lour.) Gilg
Source: Molecules. 2024 Feb 29;29(5):1075. doi: 10.3390/molecules29051075 (PMC10935121; doi:10.3390/molecules29051075)
Supplement: Supplementary file 1 [file molecules-29-01075-s001.zip › Supplementary Figure S7.pdf]

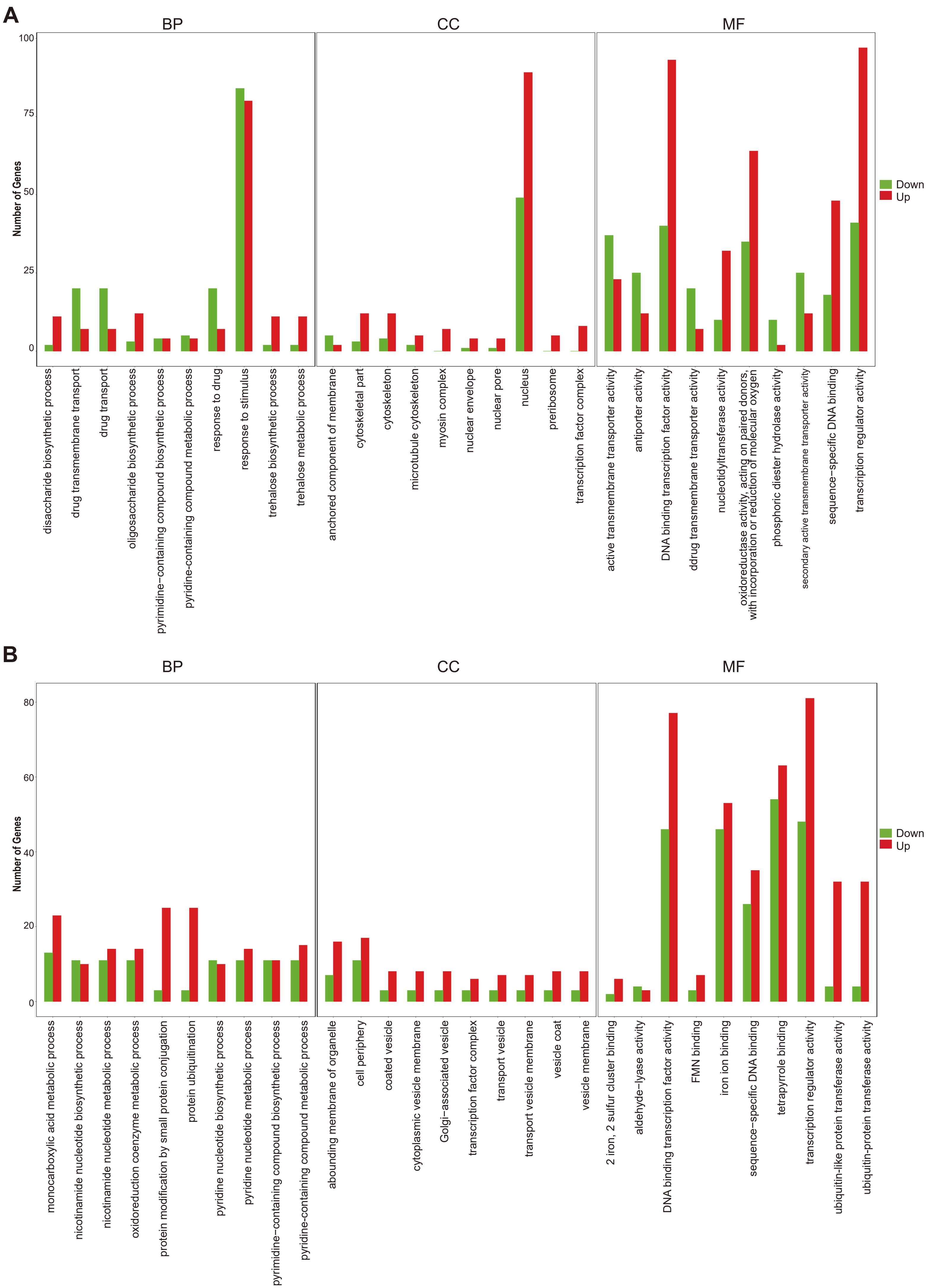

A
BP
CC
MF
Number of Genes
0
25
50
75
100
Down
Up
disaccharide biosynthetic process
drug transmembrane transport
drug transport
oligosaccharide biosynthetic process
pyrimidine−containing compound biosynthetic process
pyridine-containing compound metabolic process
response to drug
response to stimulus
trehalose biosynthetic process
trehalose metabolic process
anchored component of membrane
cytoskeletal part
cytoskeleton
microtubule cytoskeleton
myosin complex
nuclear envelope
nuclear pore
nucleus
preribosome
transcription factor complex
active transmembrane transporter activity
antiporter activity
DNA binding transcription factor activity
ddrug transmembrane transporter activity
nucleotidyltransferase activity
oxidoreductase activity, acting on paired donors, with incorporation or reduction of molecular oxygen
phosphoric diester hydrolase activity
secondary active transmembrane transporter activity
sequence−specific DNA binding
transcription regulator activity
B
BP
CC
MF
Number of Genes
0
20
40
60
80
Down
Up
monocarboxylic acid metabolic process
nicotinamide nucleotide biosynthetic process
nicotinamide nucleotide metabolic process
oxidoreduction coenzyme metabolic process
protein modification by small protein conjugation
protein ubiquitination
pyridine nucleotide biosynthetic process
pyridine nucleotide metabolic process
pyrimidine−containing compound biosynthetic process
pyridine-containing compound metabolic process
abounding membrane of organelle
cell periphery
coated vesicle
cytoplasmic vesicle membrane
Golgi−associated vesicle
transcription factor complex
transport vesicle
transport vesicle membrane
vesicle coat
vesicle membrane
2 iron, 2 sulfur cluster binding
aldehyde−lyase activity
DNA binding transcription factor activity
FMN binding
iron ion binding
sequence−specific DNA binding
tetrapyrrole binding
transcription regulator activity
ubiquitin-like protein transferase activity
ubiquitin-protein transferase activity

C

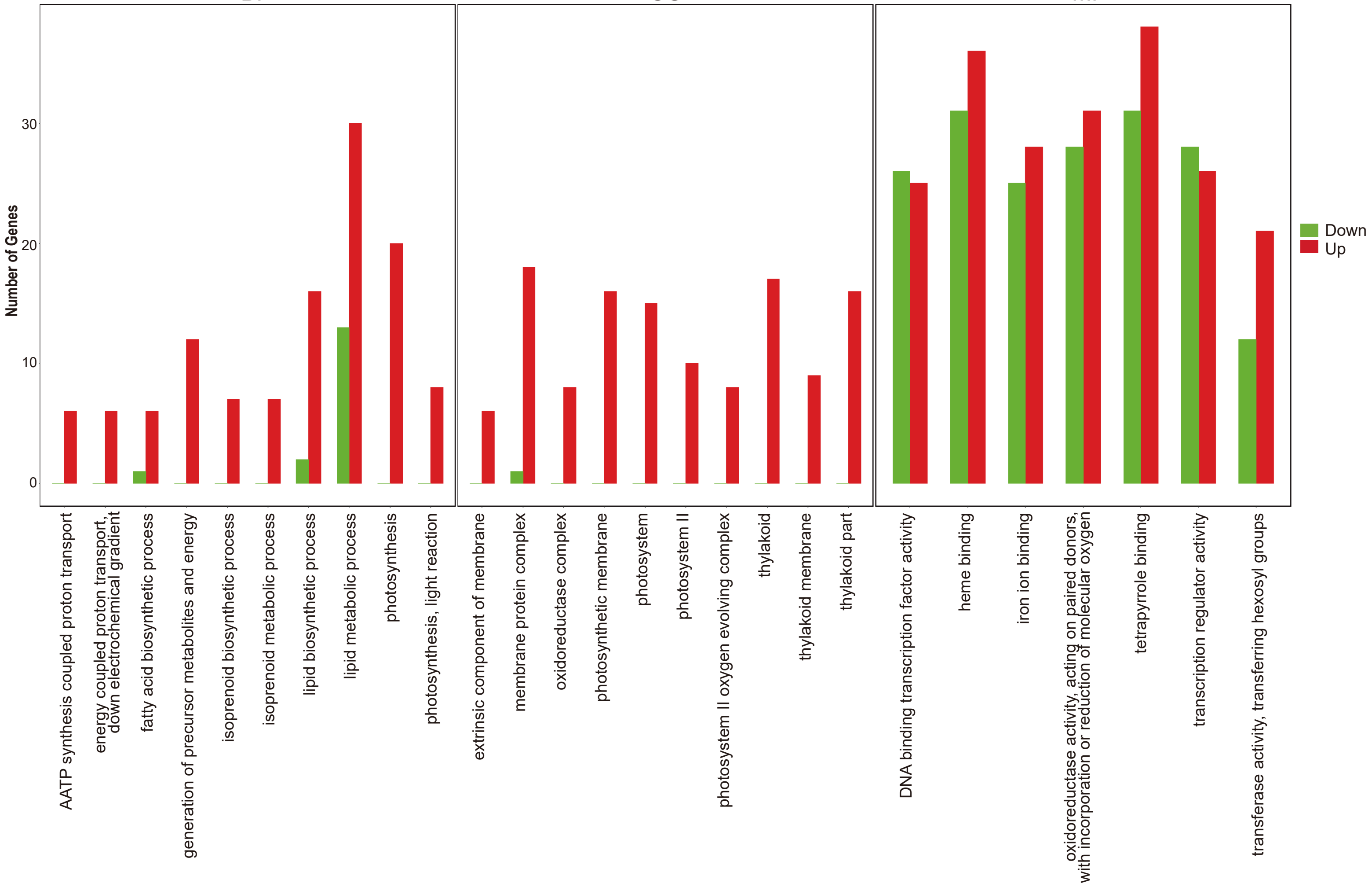
BP
CC
MF
Number of Genes
0
10
20
30
Down
Up
AATP synthesis coupled proton transport
energy coupled proton transport, down electrochemical gradient
fatty acid biosynthetic process
generation of precursor metabolites and energy
isoprenoid biosynthetic process
isoprenoid metabolic process
lipid biosynthetic process
lipid metabolic process
photosynthesis
photosynthesis, light reaction
extrinsic component of membrane
membrane protein complex
oxidoreductase complex
photosynthetic membrane
photosystem
photosystem II
photosystem II oxygen evolving complex
thylakoid
thylakoid membrane
thylakoid part
DNA binding transcription factor activity
heme binding
iron ion binding
oxidoreductase activity, acting on paired donors, with incorporation or reduction of molecular oxygen
tetrapyrrole binding
transcription regulator activity
transferase activity, transferring hexosyl groups

D

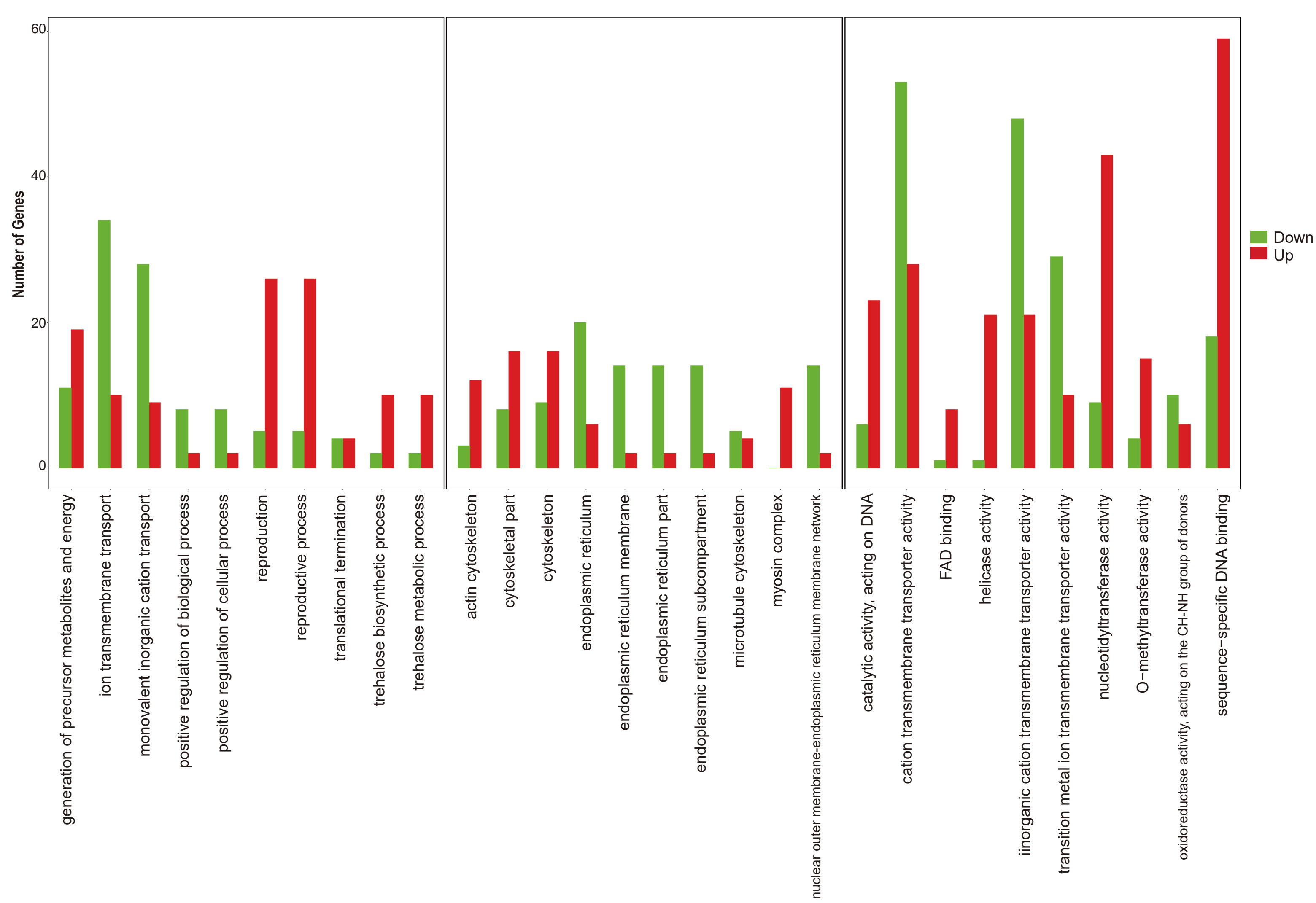
BP
CC
MF
Number of Genes
0
20
40
60
Down
Up
generation of precursor metabolites and energy
ion transmembrane transport
monovalent inorganic cation transport
positive regulation of biological process
positive regulation of cellular process
reproduction
reproductive process
translational termination
trehalose biosynthetic process
trehalose metabolic process
actin cytoskeleton
cytoskeletal part
cytoskeleton
endoplasmic reticulum
endoplasmic reticulum membrane
endoplasmic reticulum part
endoplasmic reticulum subcompartment
microtubule cytoskeleton
myosin complex
nuclear outer membrane-endoplasmic reticulum membrane network
catalytic activity, acting on DNA
cation transmembrane transporter activity
FAD binding
helicase activity
inorganic cation transmembrane transporter activity
transition metal ion transmembrane transporter activity
nucleotidyltransferase activity
O−methyltransferase activity
oxidoreductase activity, acting on the CH-NH group of donors
sequence−specific DNA binding

E

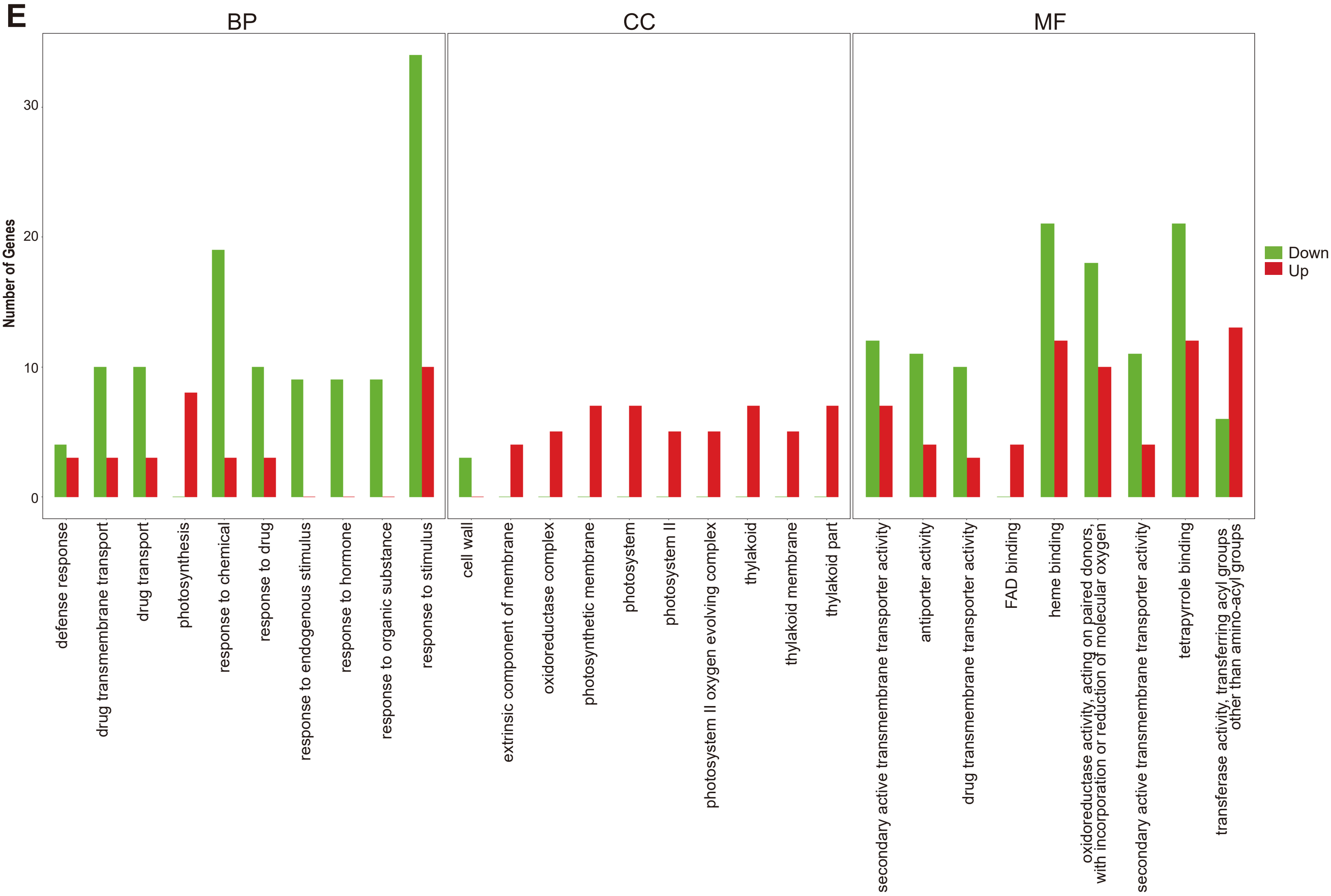
BP
CC
MF
Number of Genes
0
10
20
30
Down
Up
defense response
drug transmembrane transport
drug transport
photosynthesis
response to chemical
response to drug
response to endogenous stimulus
response to hormone
response to organic substance
response to stimulus
cell wall
extrinsic component of membrane
oxidoreductase complex
photosynthetic membrane
photosystem
photosystem II
photosystem II oxygen evolving complex
thylakoid
thylakoid membrane
thylakoid part
secondary active transmembrane transporter activity
antiporter activity
drug transmembrane transporter activity
FAD binding
heme binding
oxidoreductase activity, acting on paired donors, with incorporation or reduction of molecular oxygen
secondary active transmembrane transporter activity
tetrapyrrole binding
transferase activity, transferring acyl groups other than amino-acyl groups

F

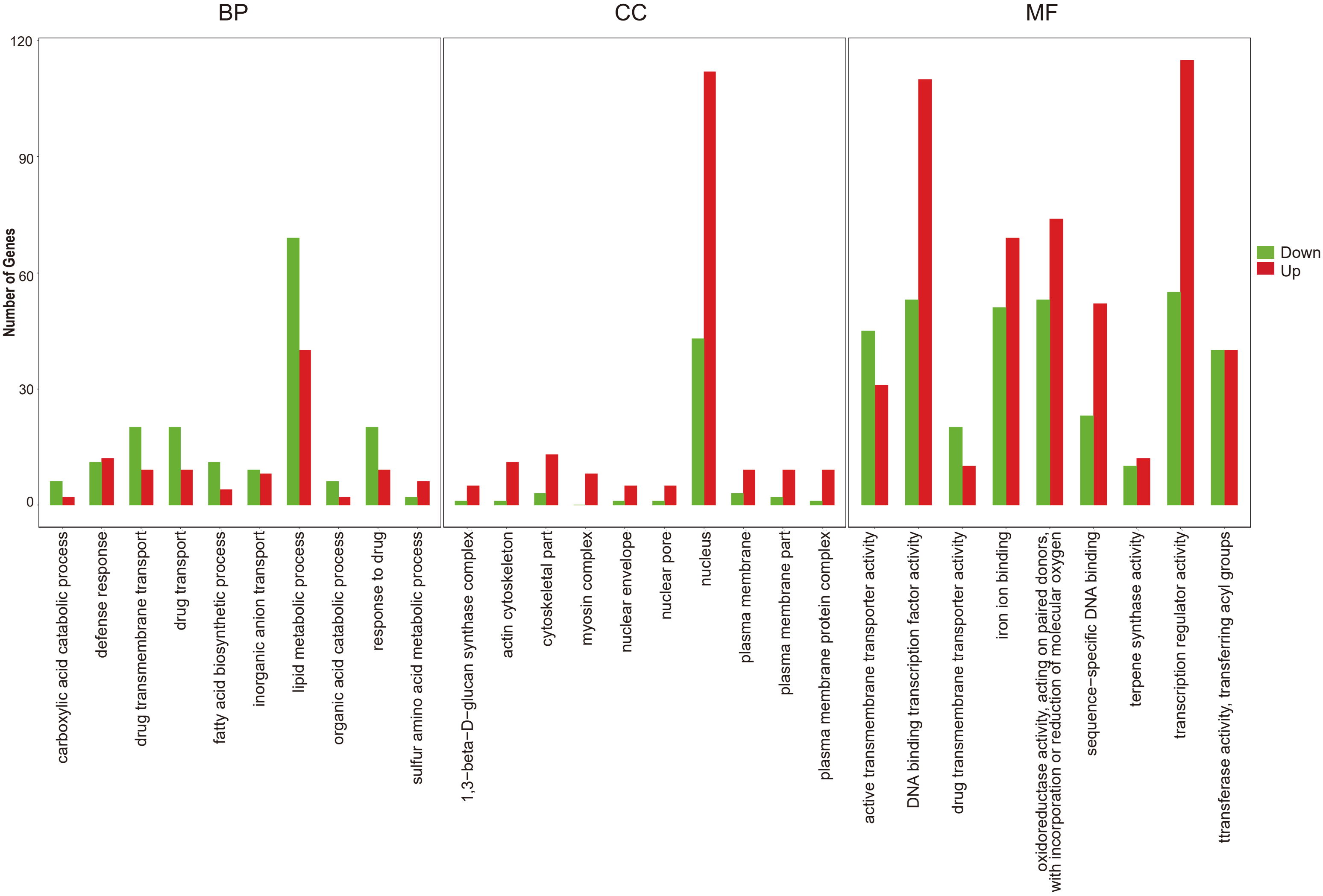
BP
CC
MF
Number of Genes
0
30
60
90
120
Down
Up
carboxylic acid catabolic process
defense response
drug transmembrane transport
drug transport
fatty acid biosynthetic process
inorganic anion transport
lipid metabolic process
organic acid catabolic process
response to drug
sulfur amino acid metabolic process
1,3−beta−D−glucan synthase complex
actin cytoskeleton
cytoskeletal part
myosin complex
nuclear envelope
nuclear pore
nucleus
plasma membrane
plasma membrane part
plasma membrane protein complex
active transmembrane transporter activity
DNA binding transcription factor activity
drug transmembrane transporter activity
iron ion binding
oxidoreductase activity, acting on paired donors, with incorporation or reduction of molecular oxygen
sequence−specific DNA binding
terpene synthase activity
transcription regulator activity
ttransferase activity, transferring acyl groups

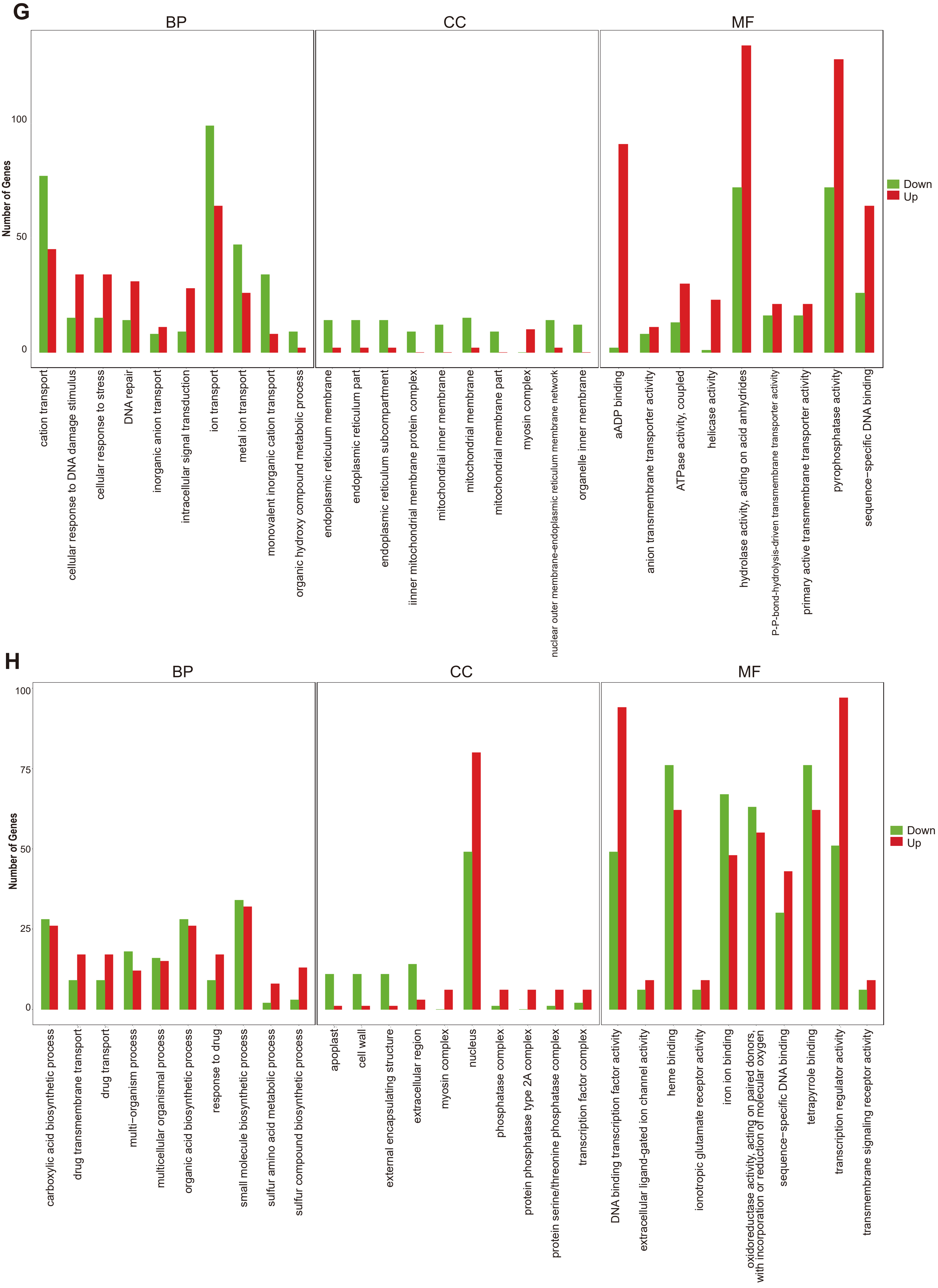
G
BP
CC
MF
Number of Genes
0
50
100
Down
Up
cation transport
cellular response to DNA damage stimulus
cellular response to stress
DNA repair
inorganic anion transport
intracellular signal transduction
ion transport
metal ion transport
monovalent inorganic cation transport
organic hydroxy compound metabolic process
endoplasmic reticulum membrane
endoplasmic reticulum part
endoplasmic reticulum subcompartment
iinner mitochondrial membrane protein complex
mitochondrial inner membrane
mitochondrial membrane
mitochondrial membrane part
myosin complex
nuclear outer membrane-endoplasmic reticulum membrane network
organelle inner membrane
aADP binding
anion transmembrane transporter activity
ATPase activity, coupled
helicase activity
hydrolase activity, acting on acid anhydrides
P-P-bond-hydrolysis-driven transmembrane transporter activity
primary active transmembrane transporter activity
pyrophosphatase activity
sequence−specific DNA binding
H
BP
CC
MF
Number of Genes
0
25
50
75
100
Down
Up
carboxylic acid biosynthetic process
drug transmembrane transport
drug transport
multi−organism process
multicellular organismal process
organic acid biosynthetic process
response to drug
small molecule biosynthetic process
sulfur amino acid metabolic process
sulfur compound biosynthetic process
apoplast
cell wall
external encapsulating structure
extracellular region
myosin complex
nucleus
phosphatase complex
protein phosphatase type 2A complex
protein serine/threonine phosphatase complex
transcription factor complex
DNA binding transcription factor activity
extracellular ligand-gated ion channel activity
heme binding
ionotropic glutamate receptor activity
iron ion binding
oxidoreductase activity, acting on paired donors,
with incorporation or reduction of molecular oxygen
sequence−specific DNA binding
tetrapyrrole binding
transcription regulator activity
transmembrane signaling receptor activity

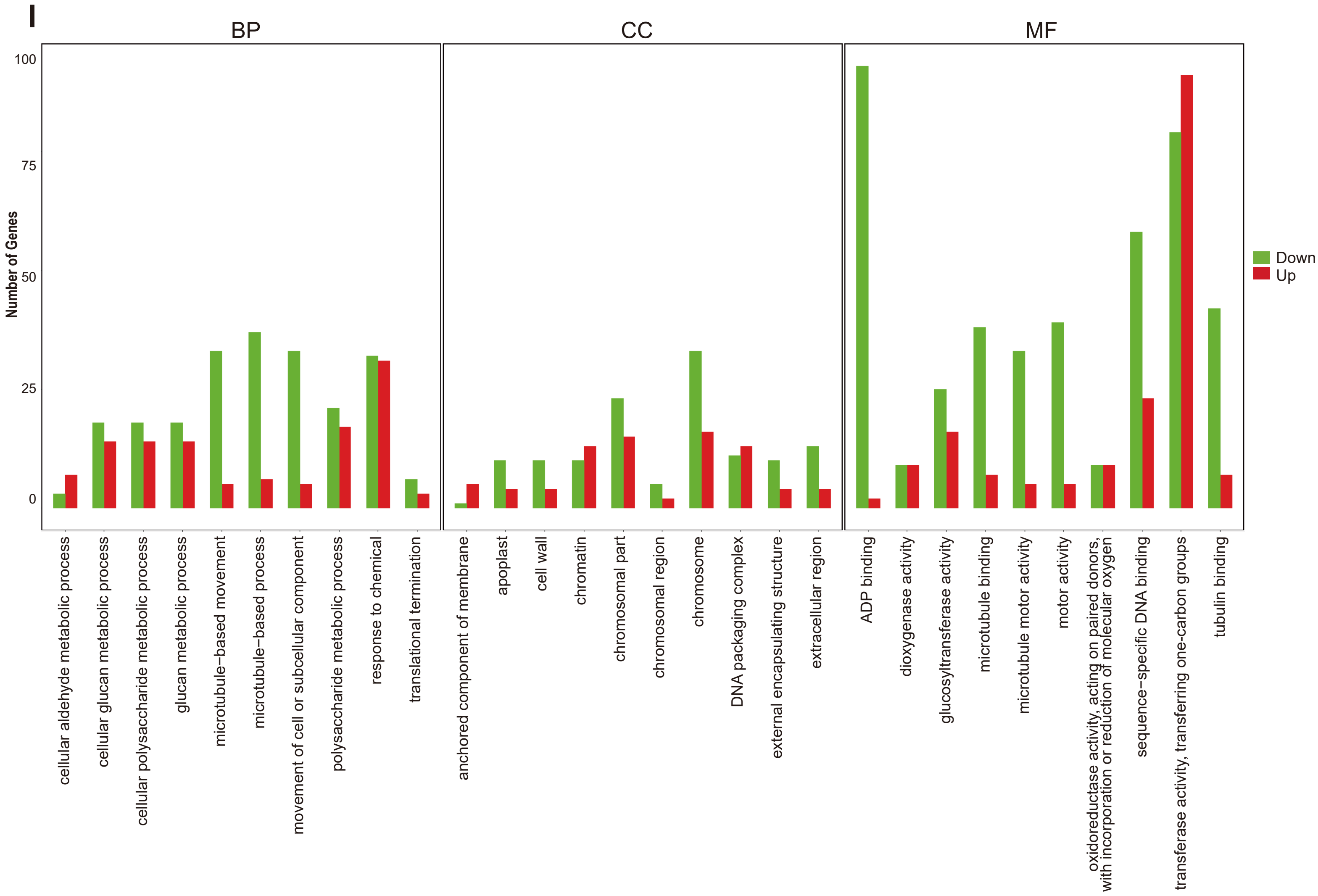
I
BP
CC
MF
Number of Genes
100
75
50
25
0
Down
Up
cellular aldehyde metabolic process
cellular glucan metabolic process
cellular polysaccharide metabolic process
glucan metabolic process
microtubule−based movement
microtubule−based process
movement of cell or subcellular component
polysaccharide metabolic process
response to chemical
translational termination
anchored component of membrane
apoplast
cell wall
chromatin
chromosomal part
chromosomal region
chromosome
DNA packaging complex
external encapsulating structure
extracellular region
ADP binding
dioxygenase activity
glucosyltransferase activity
microtubule binding
microtubule motor activity
motor activity
oxidoreductase activity, acting on paired donors, with incorporation or reduction of molecular oxygen
sequence−specific DNA binding
transferase activity, transferring one-carbon groups
tubulin binding

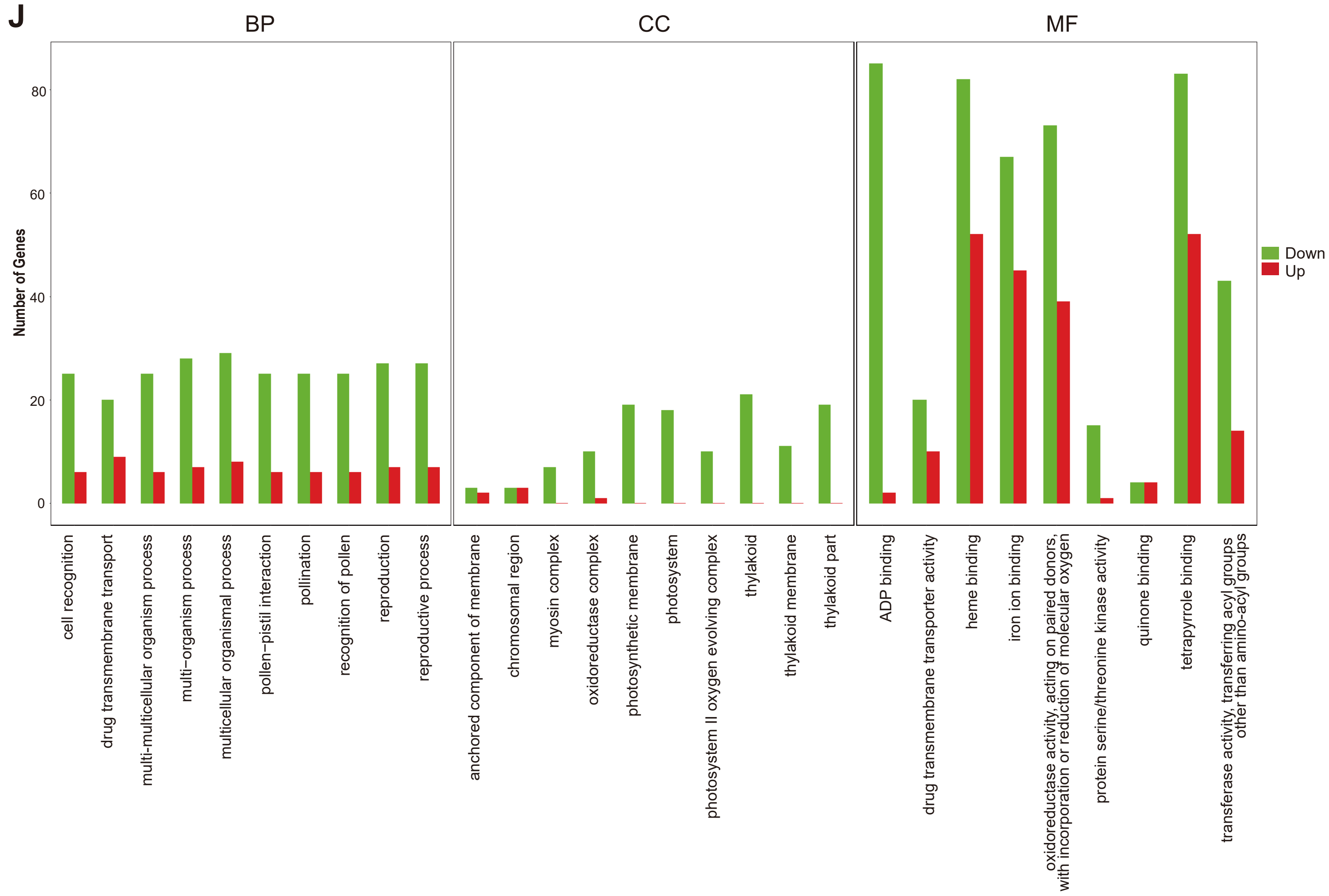
J
BP
CC
MF
Number of Genes
80
60
40
20
0
Down
Up
cell recognition
drug transmembrane transport
multi-multicellular organism process
multi−organism process
multicellular organismal process
pollen−pistil interaction
pollination
recognition of pollen
reproduction
reproductive process
anchored component of membrane
chromosomal region
myosin complex
oxidoreductase complex
photosynthetic membrane
photosystem
photosystem II oxygen evolving complex
thylakoid
thylakoid membrane
thylakoid part
ADP binding
drug transmembrane transporter activity
heme binding
iron ion binding
oxidoreductase activity, acting on paired donors, with incorporation or reduction of molecular oxygen
protein serine/threonine kinase activity
quinone binding
tetrapyrrole binding
transferase activity, transferring acyl groups other than amino-acyl groups

K

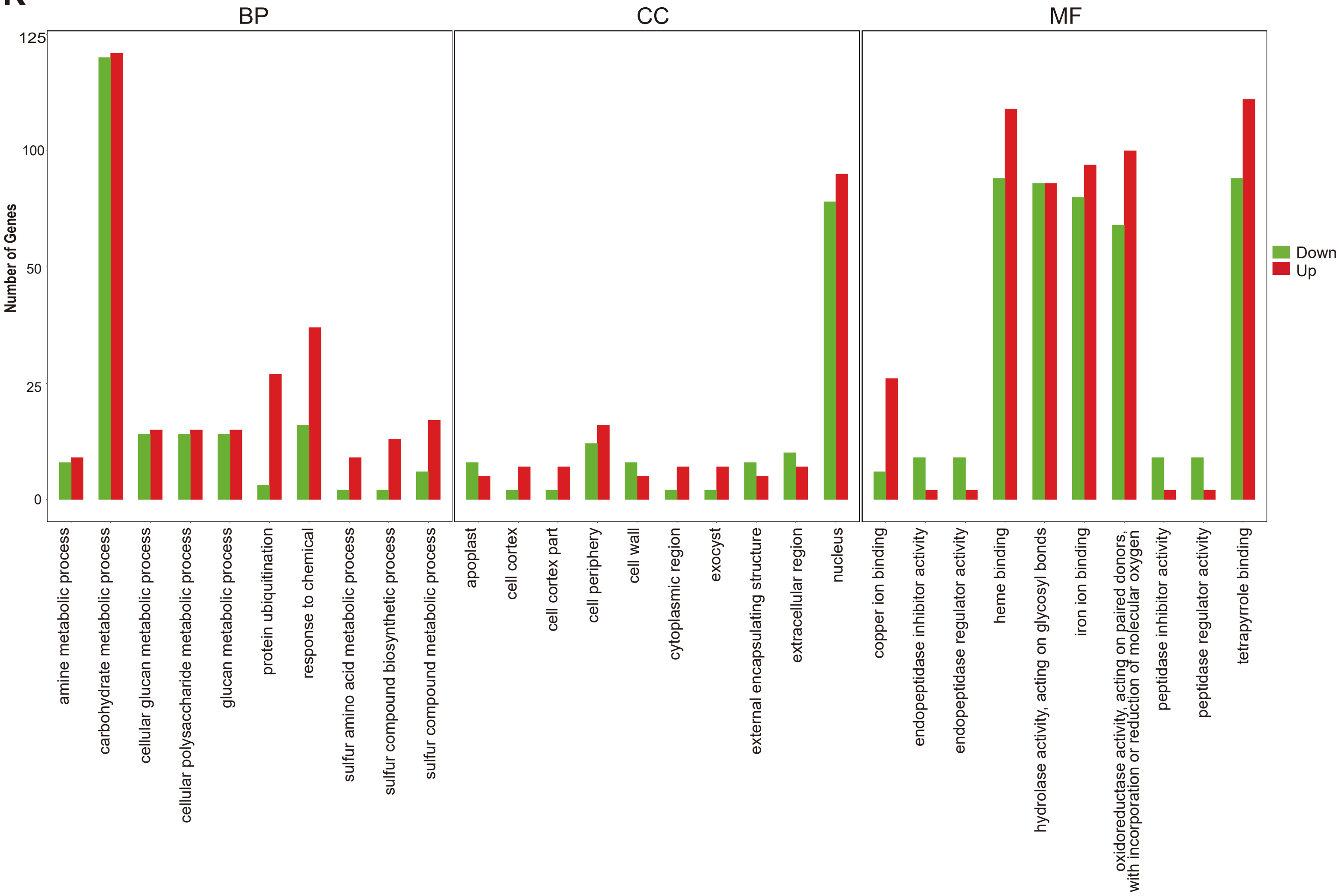
BP
CC
MF
Number of Genes
0
25
50
100
125
Down
Up
amine metabolic process
carbohydrate metabolic process
cellular glucan metabolic process
cellular polysaccharide metabolic process
glucan metabolic process
protein ubiquitination
response to chemical
sulfur amino acid metabolic process
sulfur compound biosynthetic process
sulfur compound metabolic process
apoplast
cell cortex
cell cortex part
cell periphery
cell wall
cytoplasmic region
exocyst
external encapsulating structure
extracellular region
nucleus
copper ion binding
endopeptidase inhibitor activity
endopeptidase regulator activity
heme binding
hydrolase activity, acting on glycosyl bonds
iron ion binding
oxidoreductase activity, acting on paired donors, with incorporation or reduction of molecular oxygen
peptidase inhibitor activity
peptidase regulator activity
tetrapyrrole binding

L

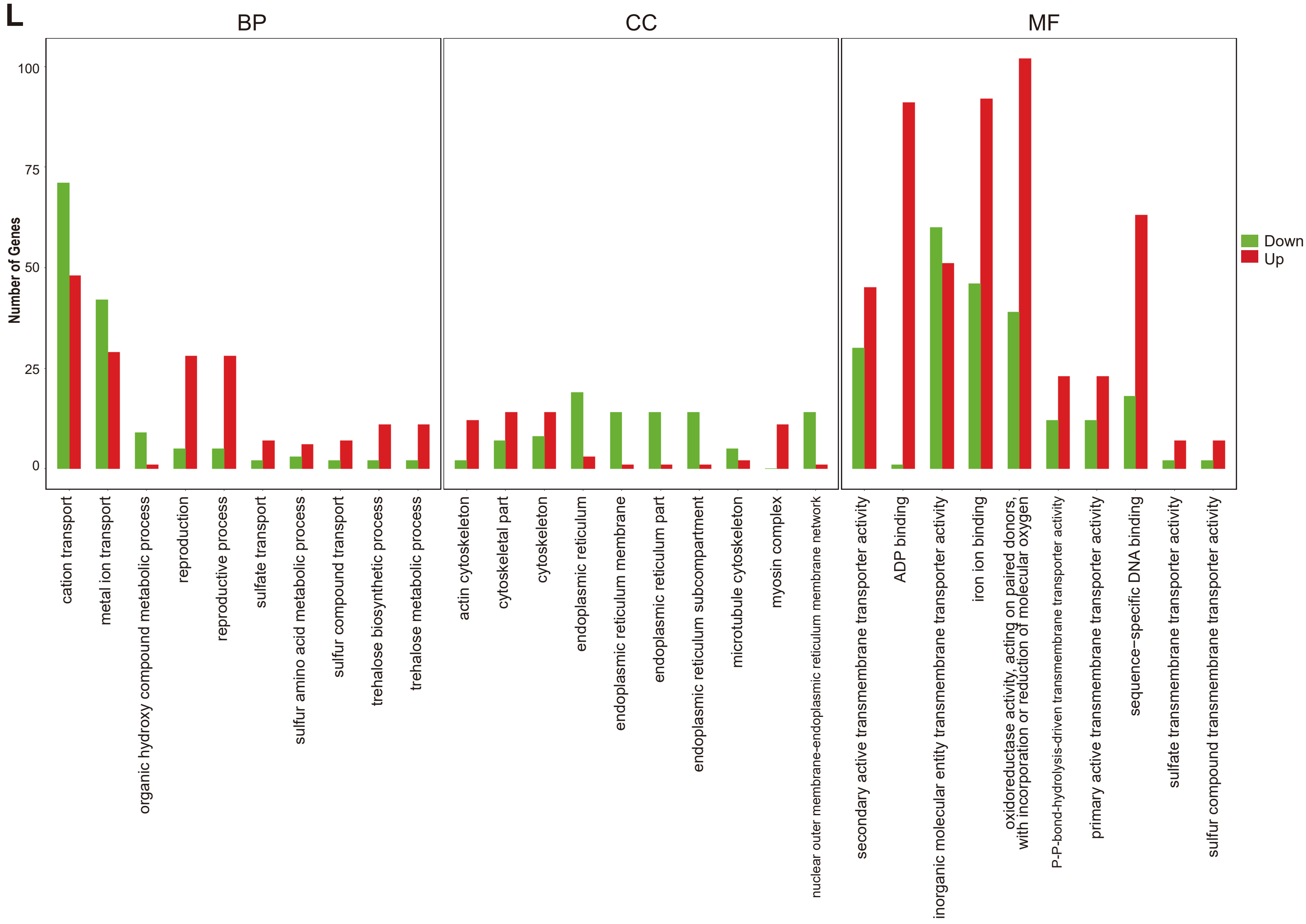
BP
CC
MF
Number of Genes
0
25
50
75
100
Down
Up
cation transport
metal ion transport
organic hydroxy compound metabolic process
reproduction
reproductive process
sulfate transport
sulfur amino acid metabolic process
sulfur compound transport
trehalose biosynthetic process
trehalose metabolic process
actin cytoskeleton
cytoskeletal part
cytoskeleton
endoplasmic reticulum
endoplasmic reticulum membrane
endoplasmic reticulum part
endoplasmic reticulum subcompartment
microtubule cytoskeleton
myosin complex
nuclear outer membrane-endoplasmic reticulum membrane network
secondary active transmembrane transporter activity
ADP binding
inorganic molecular entity transmembrane transporter activity
iron ion binding
oxidoreductase activity, acting on paired donors, with incorporation or reduction of molecular oxygen
P-P-bond-hydrolysis-driven transmembrane transporter activity
primary active transmembrane transporter activity
sequence−specific DNA binding
sulfate transmembrane transporter activity
sulfur compound transmembrane transporter activity

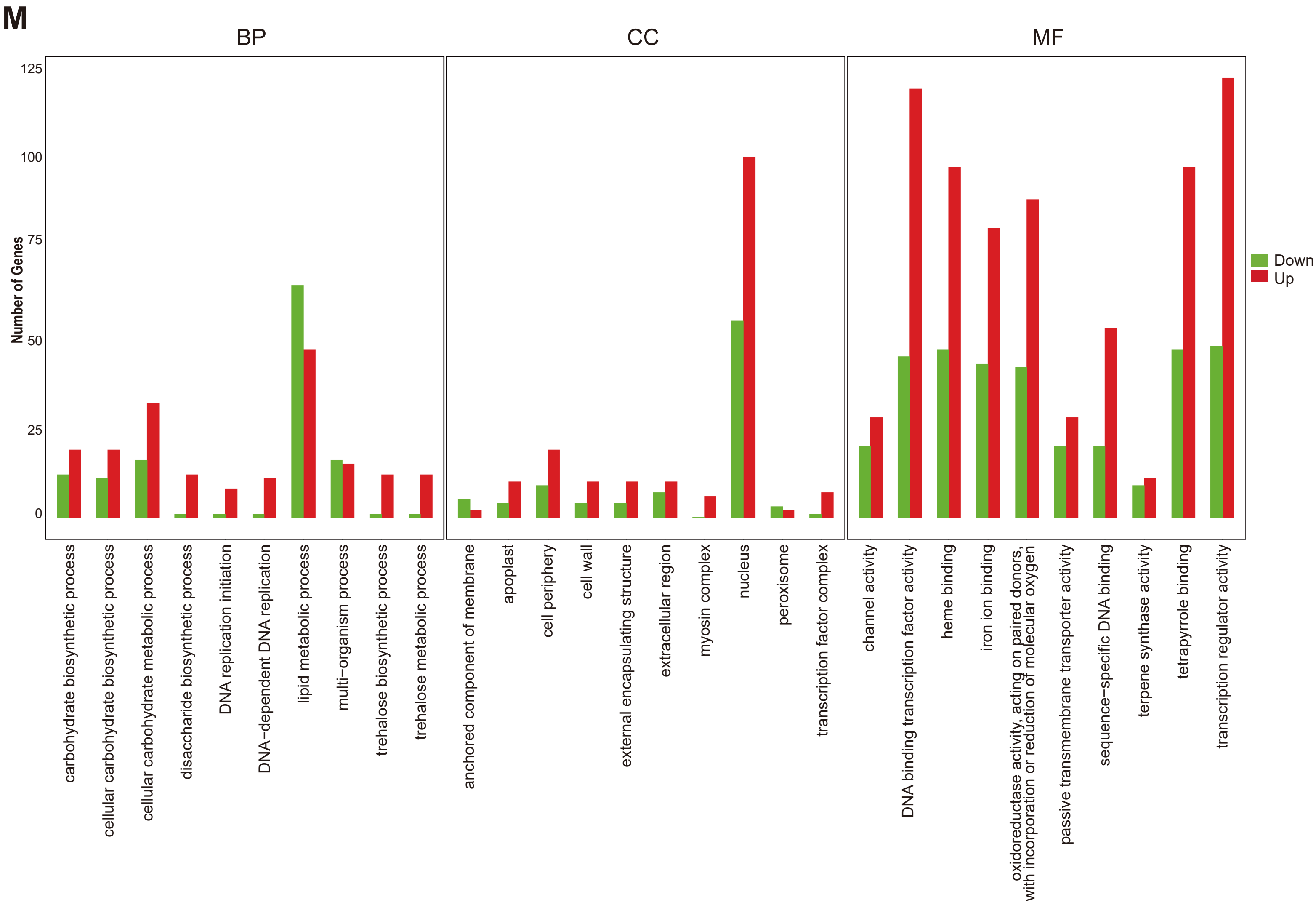

Supplementary Figure S7. Enriched GO terms of DEGs between different control groups. (A)LeafvsHusk, (B) LeafvsLeafCallus, (C) LeafvsRoot,(D) Leafvsseed ,(E)LeafvsStem,(F)RootvsHusk, (G) Rootvsseed,(H) RootvsLeafCallus,(I) SeedvsLeafCallus , (J)SeedvsHusk,(K) StemvsLeafCallus, (L) StemvsSeed, (M) Stem vsHusk.
