# Supplementary material for: Comparative Analysis of Metabolome and Transcriptome in Different Tissue Sites of Aquilaria sinensis (Lour.) Gilg
Source: Molecules. 2024 Feb 29;29(5):1075. doi: 10.3390/molecules29051075 (PMC10935121; doi:10.3390/molecules29051075)
Supplement: Supplementary file 1 [file molecules-29-01075-s001.zip › Supplementary Figure S8.pdf]

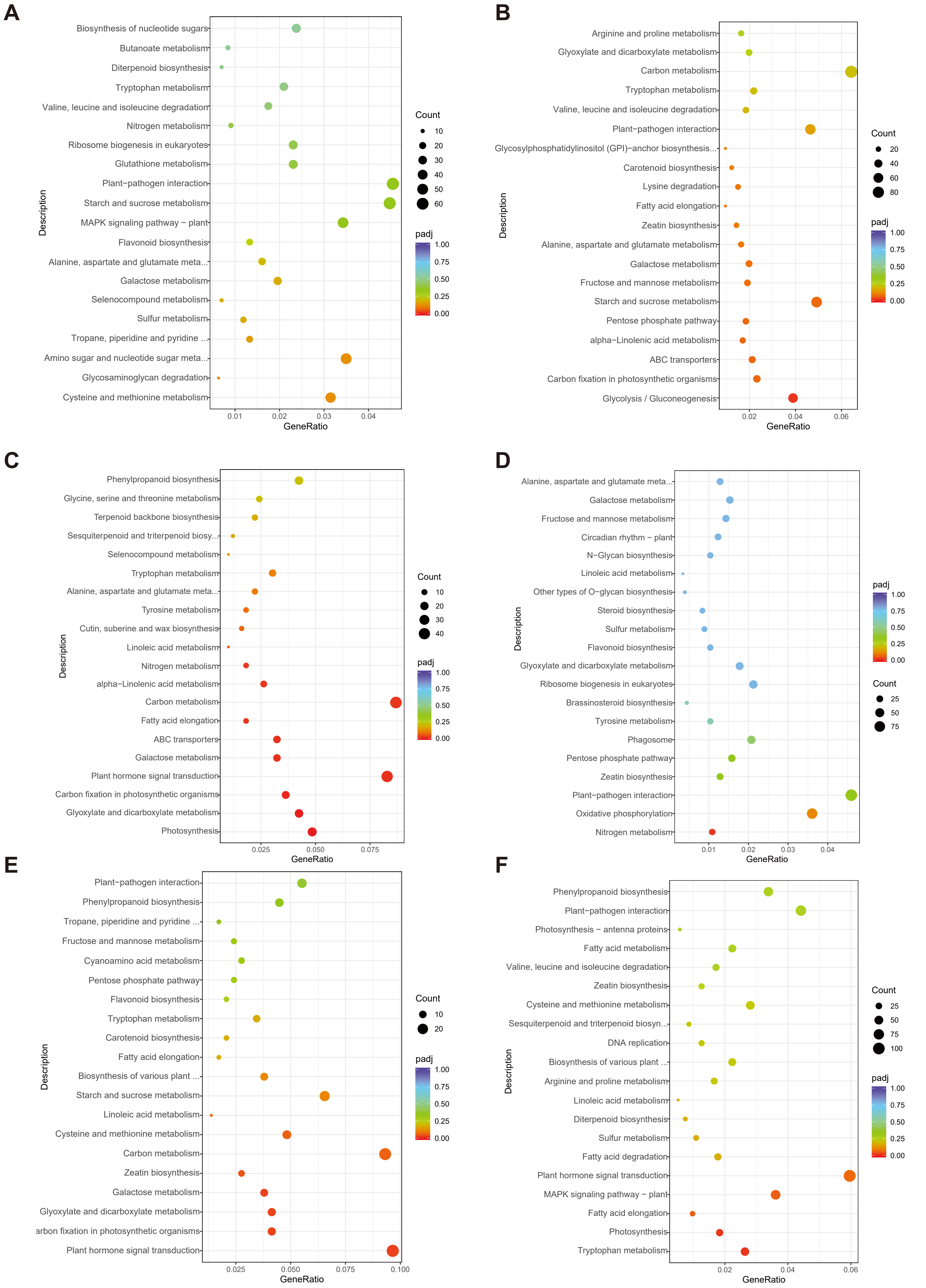
A
Biosynthesis of nucleotide sugars
Butanoate metabolism
Diterpenoid biosynthesis
Tryptophan metabolism
Valine, leucine and isoleucine degradation
Nitrogen metabolism
Ribosome biogenesis in eukaryotes
Glutathione metabolism
Plant−pathogen interaction
Starch and sucrose metabolism
MAPK signaling pathway − plant
Flavonoid biosynthesis
Alanine, aspartate and glutamate meta...
Galactose metabolism
Selenocompound metabolism
Sulfur metabolism
Tropane, piperidine and pyridine ...
Amino sugar and nucleotide sugar meta...
Glycosaminoglycan degradation
Cysteine and methionine metabolism
Description
GeneRatio
0.01 0.02 0.03 0.04
Count
10 20 30 40 50 60
padj
1.00 0.75 0.50 0.25 0.00
B
Arginine and proline metabolism
Glyoxylate and dicarboxylate metabolism
Carbon metabolism
Tryptophan metabolism
Valine, leucine and isoleucine degradation
Plant−pathogen interaction
Glycosylphosphatidylinositol (GPI)−anchor biosynthesis...
Carotenoid biosynthesis
Lysine degradation
Fatty acid elongation
Zeatin biosynthesis
Alanine, aspartate and glutamate metabolism
Galactose metabolism
Fructose and mannose metabolism
Starch and sucrose metabolism
Pentose phosphate pathway
alpha−Linolenic acid metabolism
ABC transporters
Carbon fixation in photosynthetic organisms
Glycolysis / Gluconeogenesis
Description
GeneRatio
0.02 0.04 0.06
Count
20 40 60 80
padj
1.00 0.75 0.50 0.25 0.00
C
Phenylpropanoid biosynthesis
Glycine, serine and threonine metabolism
Terpenoid backbone biosynthesis
Sesquiterpenoid and triterpenoid biosy...
Selenocompound metabolism
Tryptophan metabolism
Alanine, aspartate and glutamate meta...
Tyrosine metabolism
Cutin, suberine and wax biosynthesis
Linoleic acid metabolism
Nitrogen metabolism
alpha−Linolenic acid metabolism
Carbon metabolism
Fatty acid elongation
ABC transporters
Galactose metabolism
Plant hormone signal transduction
Carbon fixation in photosynthetic organisms
Glyoxylate and dicarboxylate metabolism
Photosynthesis
Description
GeneRatio
0.025 0.050 0.075
Count
10 20 30 40
padj
1.00 0.75 0.50 0.25 0.00
D
Alanine, aspartate and glutamate meta...
Galactose metabolism
Fructose and mannose metabolism
Circadian rhythm − plant
N−Glycan biosynthesis
Linoleic acid metabolism
Other types of O−glycan biosynthesis
Steroid biosynthesis
Sulfur metabolism
Flavonoid biosynthesis
Glyoxylate and dicarboxylate metabolism
Ribosome biogenesis in eukaryotes
Brassinosteroid biosynthesis
Tyrosine metabolism
Phagosome
Pentose phosphate pathway
Zeatin biosynthesis
Plant−pathogen interaction
Oxidative phosphorylation
Nitrogen metabolism
Description
GeneRatio
0.01 0.02 0.03 0.04
padj
1.00 0.75 0.50 0.25 0.00
Count
25 50 75
E
Plant−pathogen interaction
Phenylpropanoid biosynthesis
Tropane, piperidine and pyridine ...
Fructose and mannose metabolism
Cyanoamino acid metabolism
Pentose phosphate pathway
Flavonoid biosynthesis
Tryptophan metabolism
Carotenoid biosynthesis
Fatty acid elongation
Biosynthesis of various plant ...
Starch and sucrose metabolism
Linoleic acid metabolism
Cysteine and methionine metabolism
Carbon metabolism
Zeatin biosynthesis
Galactose metabolism
Glyoxylate and dicarboxylate metabolism
arbon fixation in photosynthetic organisms
Plant hormone signal transduction
Description
GeneRatio
0.025 0.050 0.075 0.100
Count
10 20
padj
1.00 0.75 0.50 0.25 0.00
F
Phenylpropanoid biosynthesis
Plant−pathogen interaction
Photosynthesis − antenna proteins
Fatty acid metabolism
Valine, leucine and isoleucine degradation
Zeatin biosynthesis
Cysteine and methionine metabolism
Sesquiterpenoid and triterpenoid biosyn...
DNA replication
Biosynthesis of various plant ...
Arginine and proline metabolism
Linoleic acid metabolism
Diterpenoid biosynthesis
Sulfur metabolism
Fatty acid degradation
Plant hormone signal transduction
MAPK signaling pathway − plant
Fatty acid elongation
Photosynthesis
Tryptophan metabolism
Description
GeneRatio
0.02 0.04 0.06
Count
25 50 75 100
padj
1.00 0.75 0.50 0.25 0.00

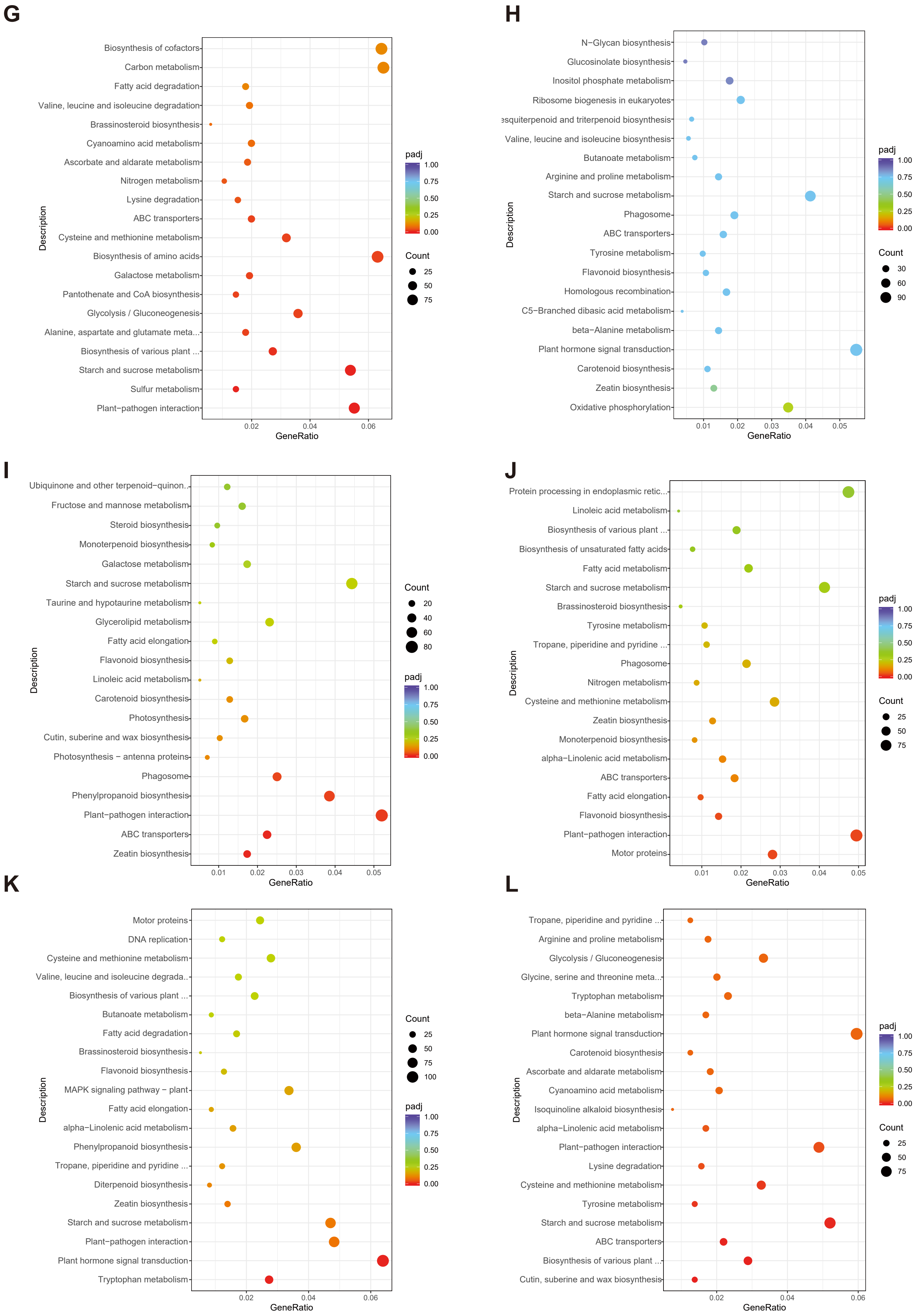
G
Biosynthesis of cofactors
Carbon metabolism
Fatty acid degradation
Valine, leucine and isoleucine degradation
Brassinosteroid biosynthesis
Cyanoamino acid metabolism
Ascorbate and aldarate metabolism
Nitrogen metabolism
Lysine degradation
ABC transporters
Cysteine and methionine metabolism
Biosynthesis of amino acids
Galactose metabolism
Pantothenate and CoA biosynthesis
Glycolysis / Gluconeogenesis
Alanine, aspartate and glutamate meta...
Biosynthesis of various plant ...
Starch and sucrose metabolism
Sulfur metabolism
Plant−pathogen interaction
Description
GeneRatio
0.02
0.04
0.06
padj
1.00
0.75
0.50
0.25
0.00
Count
25
50
75
H
N−Glycan biosynthesis
Glucosinolate biosynthesis
Inositol phosphate metabolism
Ribosome biogenesis in eukaryotes
esquiterpenoid and triterpenoid biosynthesis
Valine, leucine and isoleucine biosynthesis
Butanoate metabolism
Arginine and proline metabolism
Starch and sucrose metabolism
Phagosome
ABC transporters
Tyrosine metabolism
Flavonoid biosynthesis
Homologous recombination
C5−Branched dibasic acid metabolism
beta−Alanine metabolism
Plant hormone signal transduction
Carotenoid biosynthesis
Zeatin biosynthesis
Oxidative phosphorylation
Description
GeneRatio
0.01
0.02
0.03
0.04
0.05
padj
1.00
0.75
0.50
0.25
0.00
Count
30
60
90
I
Ubiquinone and other terpenoid−quinon...
Fructose and mannose metabolism
Steroid biosynthesis
Monoterpenoid biosynthesis
Galactose metabolism
Starch and sucrose metabolism
Taurine and hypotaurine metabolism
Glycerolipid metabolism
Fatty acid elongation
Flavonoid biosynthesis
Linoleic acid metabolism
Carotenoid biosynthesis
Photosynthesis
Cutin, suberine and wax biosynthesis
Photosynthesis − antenna proteins
Phagosome
Phenylpropanoid biosynthesis
Plant−pathogen interaction
ABC transporters
Zeatin biosynthesis
Description
GeneRatio
0.01
0.02
0.03
0.04
0.05
Count
20
40
60
80
padj
1.00
0.75
0.50
0.25
0.00
J
Protein processing in endoplasmic retic...
Linoleic acid metabolism
Biosynthesis of various plant ...
Biosynthesis of unsaturated fatty acids
Fatty acid metabolism
Starch and sucrose metabolism
Brassinosteroid biosynthesis
Tyrosine metabolism
Tropane, piperidine and pyridine ...
Phagosome
Nitrogen metabolism
Cysteine and methionine metabolism
Zeatin biosynthesis
Monoterpenoid biosynthesis
alpha−Linolenic acid metabolism
ABC transporters
Fatty acid elongation
Flavonoid biosynthesis
Plant−pathogen interaction
Motor proteins
Description
GeneRatio
0.01
0.02
0.03
0.04
0.05
padj
1.00
0.75
0.50
0.25
0.00
Count
25
50
75
K
Motor proteins
DNA replication
Cysteine and methionine metabolism
Valine, leucine and isoleucine degrada...
Biosynthesis of various plant ...
Butanoate metabolism
Fatty acid degradation
Brassinosteroid biosynthesis
Flavonoid biosynthesis
MAPK signaling pathway − plant
Fatty acid elongation
alpha−Linolenic acid metabolism
Phenylpropanoid biosynthesis
Tropane, piperidine and pyridine ...
Diterpenoid biosynthesis
Zeatin biosynthesis
Starch and sucrose metabolism
Plant−pathogen interaction
Plant hormone signal transduction
Tryptophan metabolism
Description
GeneRatio
0.02
0.04
0.06
Count
25
50
75
100
padj
1.00
0.75
0.50
0.25
0.00
L
Tropane, piperidine and pyridine ...
Arginine and proline metabolism
Glycolysis / Gluconeogenesis
Glycine, serine and threonine meta...
Tryptophan metabolism
beta−Alanine metabolism
Plant hormone signal transduction
Carotenoid biosynthesis
Ascorbate and aldarate metabolism
Cyanoamino acid metabolism
Isoquinoline alkaloid biosynthesis
alpha−Linolenic acid metabolism
Plant−pathogen interaction
Lysine degradation
Cysteine and methionine metabolism
Tyrosine metabolism
Starch and sucrose metabolism
ABC transporters
Biosynthesis of various plant ...
Cutin, suberine and wax biosynthesis
Description
GeneRatio
0.02
0.04
0.06
padj
1.00
0.75
0.50
0.25
0.00
Count
25
50
75

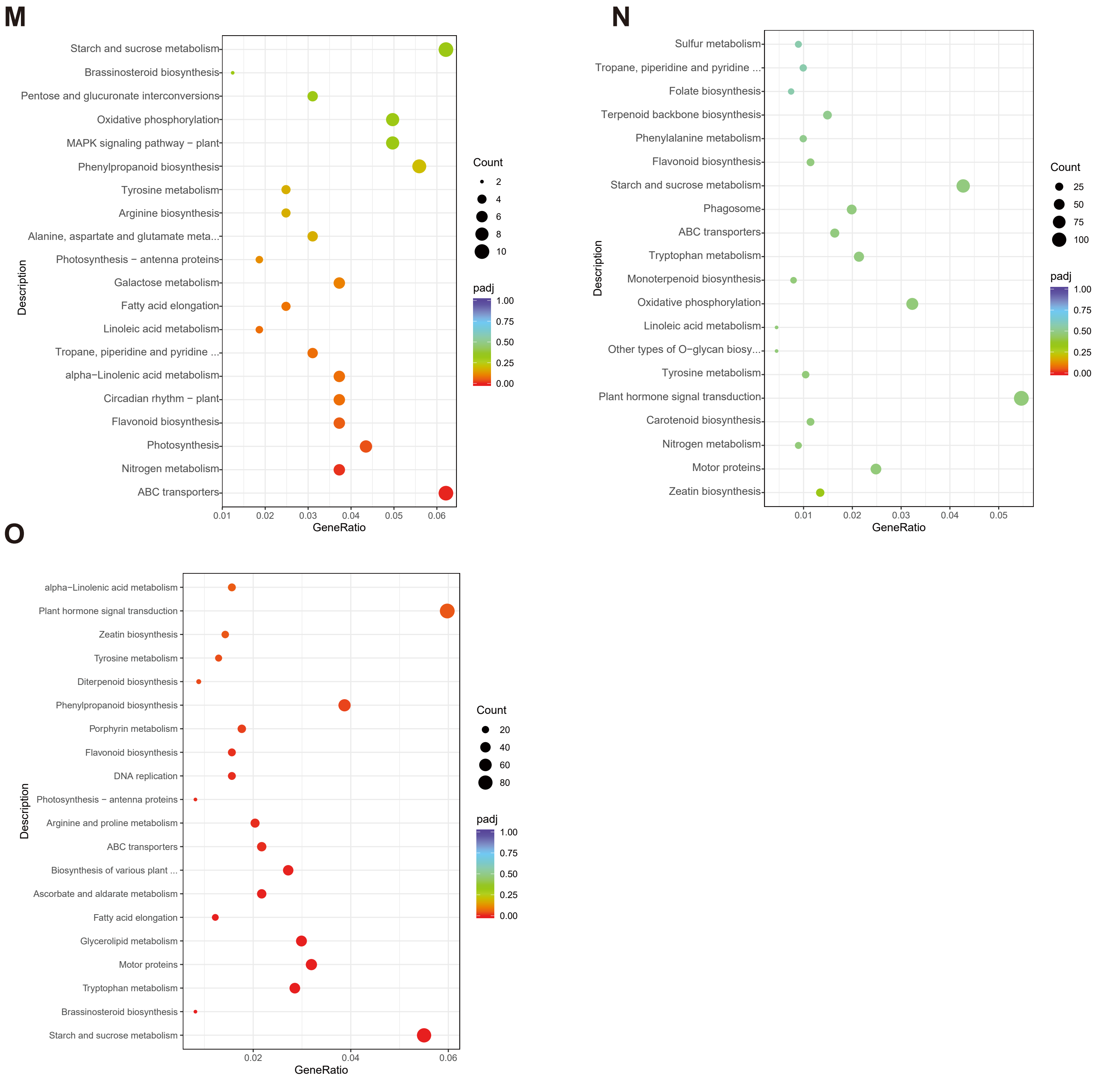

Supplementary Figure S8. Enriched KEGG pathway of DEGs between different control groups. (A) LeafvsHusk, (B) LeafvsLeafCallus, (C)LeafvsRoot,(D) LeafvsSeed,(E) LeafvsStem , (F) Root vsHusk,(G) RootvsLeafCallus, (H)RootvsSeed,(I) SeedvsHusk,(J) SeedvsLeafCallus, (K) Stem vsHusk, (L) StemvsLeafCallus, (M)StemvsRoot, (N) StemvsSeed, (O) HuskvsLeafCallus.
