# Supplementary material for: Comparative Analysis of Metabolome and Transcriptome in Different Tissue Sites of Aquilaria sinensis (Lour.) Gilg
Source: Molecules. 2024 Feb 29;29(5):1075. doi: 10.3390/molecules29051075 (PMC10935121; doi:10.3390/molecules29051075)
Supplement: Supplementary file 1 [file molecules-29-01075-s001.zip › Supplementary Figure S9.pdf]

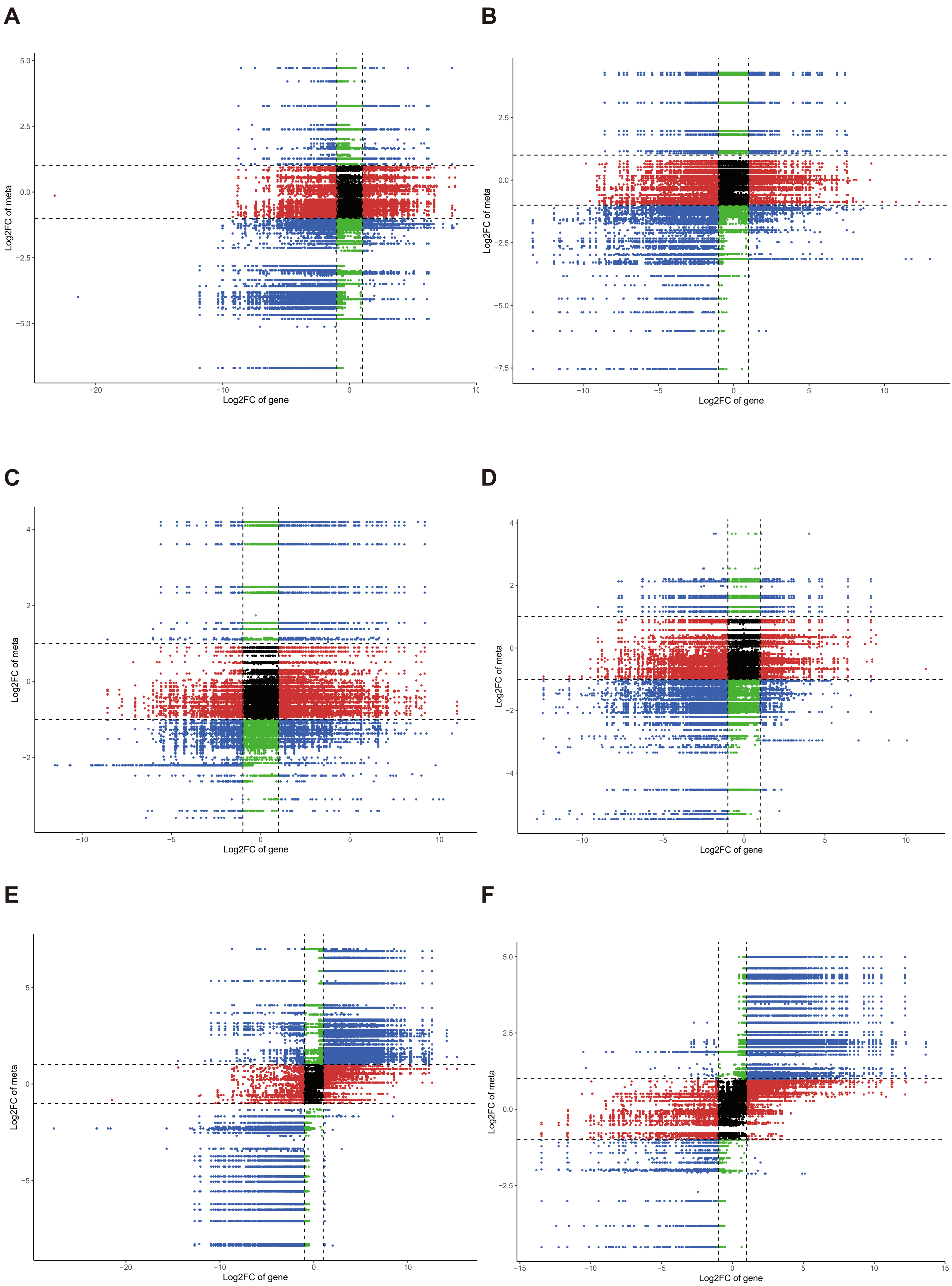
A
B
C
D
E
F
Log2FC of meta
Log2FC of gene

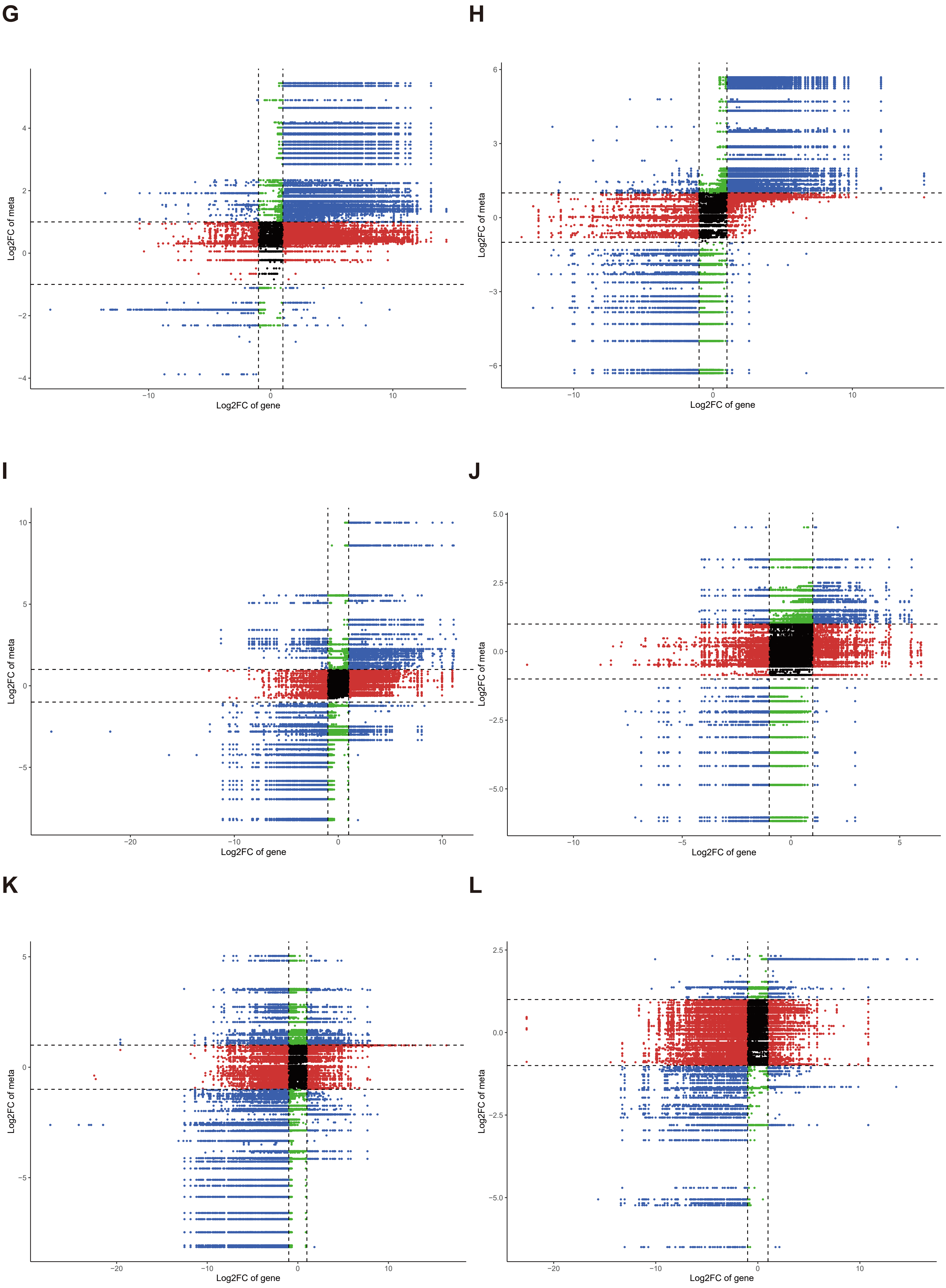
G
H
I
J
K
L
Log2FC of meta
Log2FC of gene

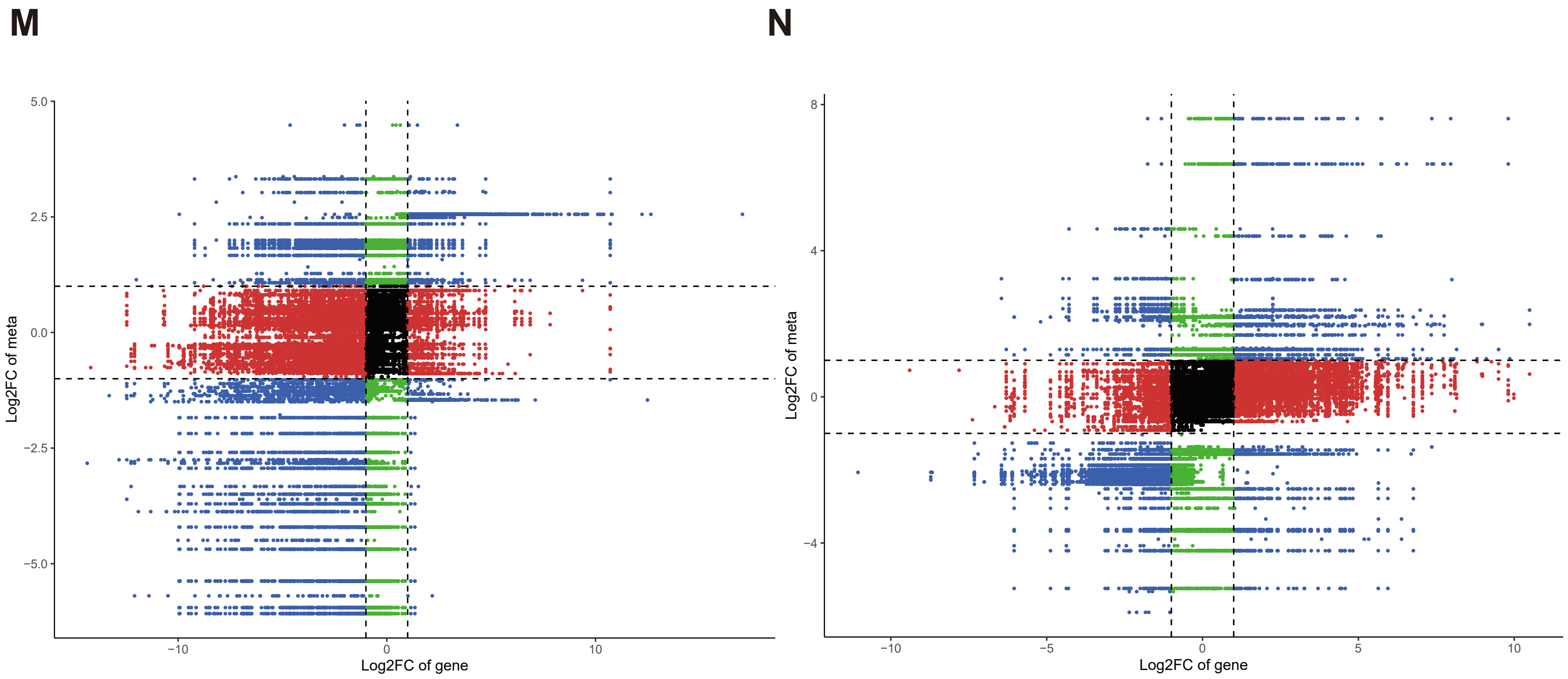

Supplementary Figure S9. Nine quadrant diagram showing the correlation of differentially expressed genes and differentially accumulated metabolites.(A)HuskvsLeaf,(B) HuskvsRoot,(C) HuskvsSeed,(D)HuskvsStem,(E)LeafCallusvsLeaf,(F)LeafCallusvsRoot,(GLeafCallusvsSeed,(H) LeafCallusvsStem,(I)RootvsLeaf,(J)RootvsStem,(K)SeedvsLeaf,(L)SeedvsRoot,(M)SeedvsSeed,(N) StemvsLeaf.
